# Supplementary material for: Establishment of a Stable BK Polyomavirus-Secreting Cell Line: Characterization of Viral Genome Integration and Replication Dynamics Through Comprehensive Analysis
Source: Int J Mol Sci. 2025 Jun 15;26(12):5745. doi: 10.3390/ijms26125745 (PMC12193623; doi:10.3390/ijms26125745)
Supplement: Supplementary file 1 [file ijms-26-05745-s001.zip › supplementary figures.pdf]

Supplementary figures

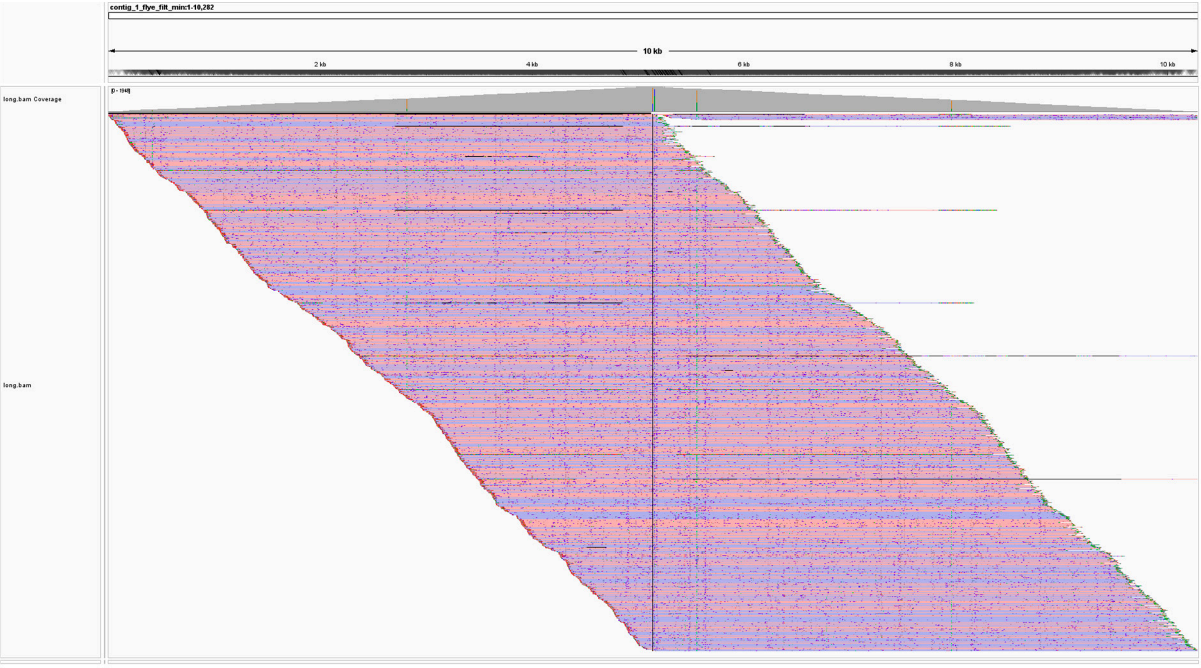

Supplementary Figure S1. IGV pile-up visualization of the read assembly for circular BKPyV used for infecting COS-WT cell line.

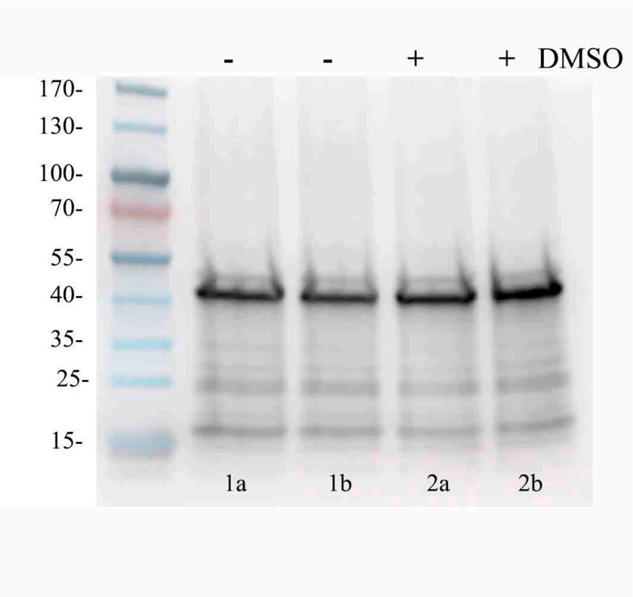

Supplementary Figure S2. VP1 immunoblot. The analysis was performed on protein extracts from COSSA cells cultured under standard conditions and following treatment with DMSO for 48 hours. The protein extract from $2x10^4$ COSSA cells was loaded on SDS-PAGE and the blot was incubated with polyclonal rabbit anti-VP1 and finally visualised by chemiluminescence. Gel loading was performed in duplicate for each control (1a and 1b) and DMSO (diluted 1:1000, 2a and 2b) to correct for technical errors. DMSO diluted at 1:1000 in culture media had no influence on VP1 expression.

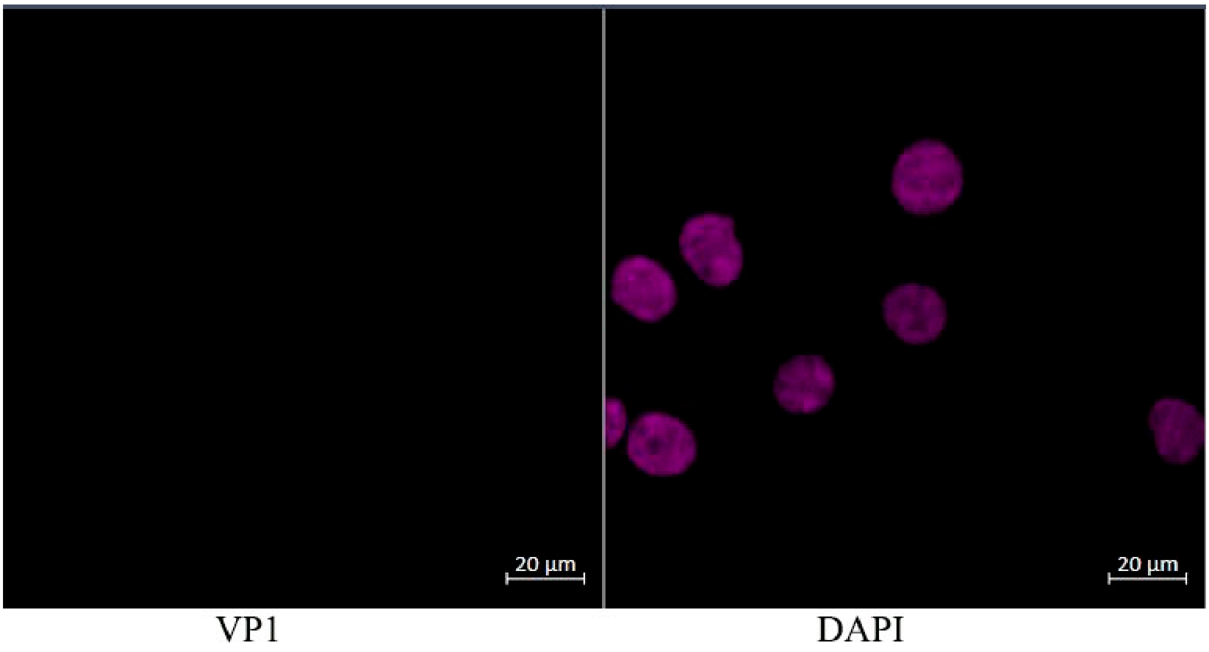

Supplementary Figure S3 Confocal immunofluorescence analysis of renal tubular epithelial progenitor cell line. Confocal microscopy was performed before infection to detect BKPyV VP1 expression. Cytopreparations were fixed with acetone and stained using the BKPyV VP1-specific monoclonal antibody mAb 4942. Nuclear counterstaining was performed with DAPI.

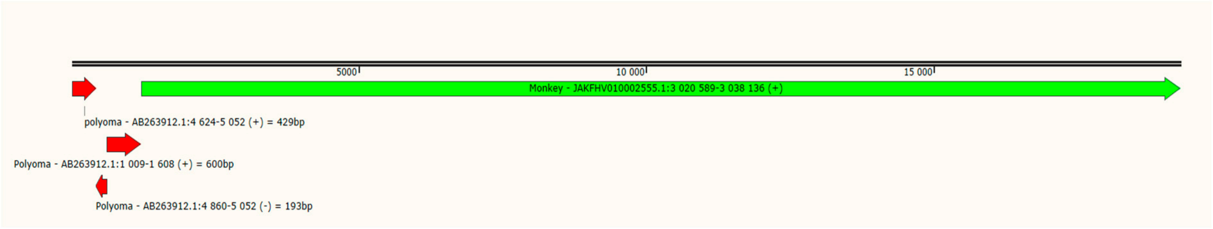

Supplementary Figure S4. Schematic depiction of contig 1. Integration of a 1200 bp fragment of the BKPyV genome (red). Flanking sequences corresponding to the monkey host genome (green) were also identified, confirming the integration event.

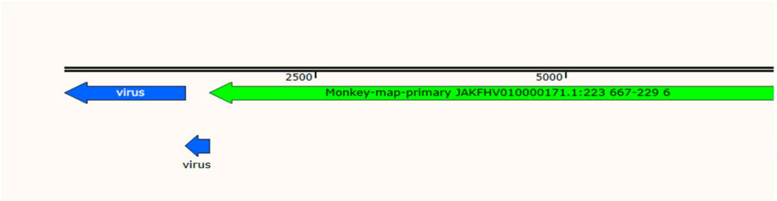

Supplementary Figure S5. Schematic depiction of contig 2. Integration of a 2944 bp fragment of the BKPyV genome (blue). Flanking sequences corresponding to the monkey host genome (green) were also identified, confirming the integration event.

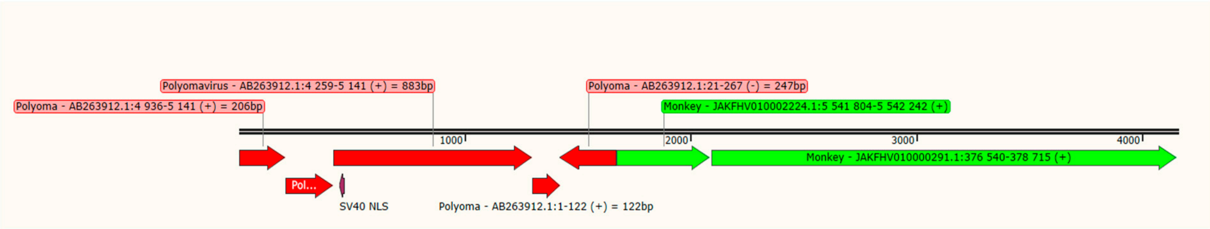

Supplementary Figure S6. Schematic depiction of contig 3. Integration of a 1668 bp fragment of the BKPyV genome (red). Flanking sequences corresponding to the monkey host genome (green) were also identified, confirming the integration event.

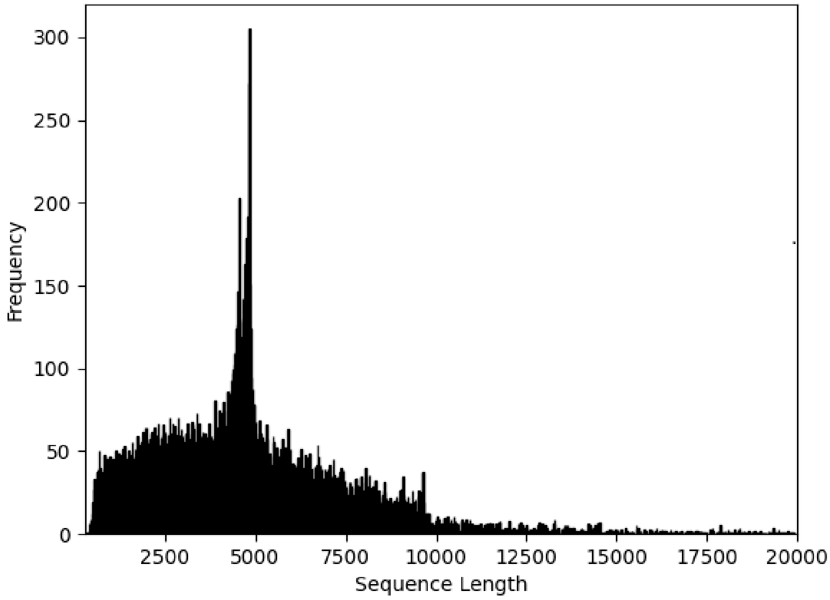

Supplementary Figure S7. Histogram of the sequence length distribution of BKPyV-specific reads. The reads unmapped to the monkey genome revealed a biphasic pattern, with a predominant peak com-prising over 300 reads at 4820 bp along with an additional subvariant of 4815 bp and a secondary peak consisting of approximately 200 reads at 4540 bp.

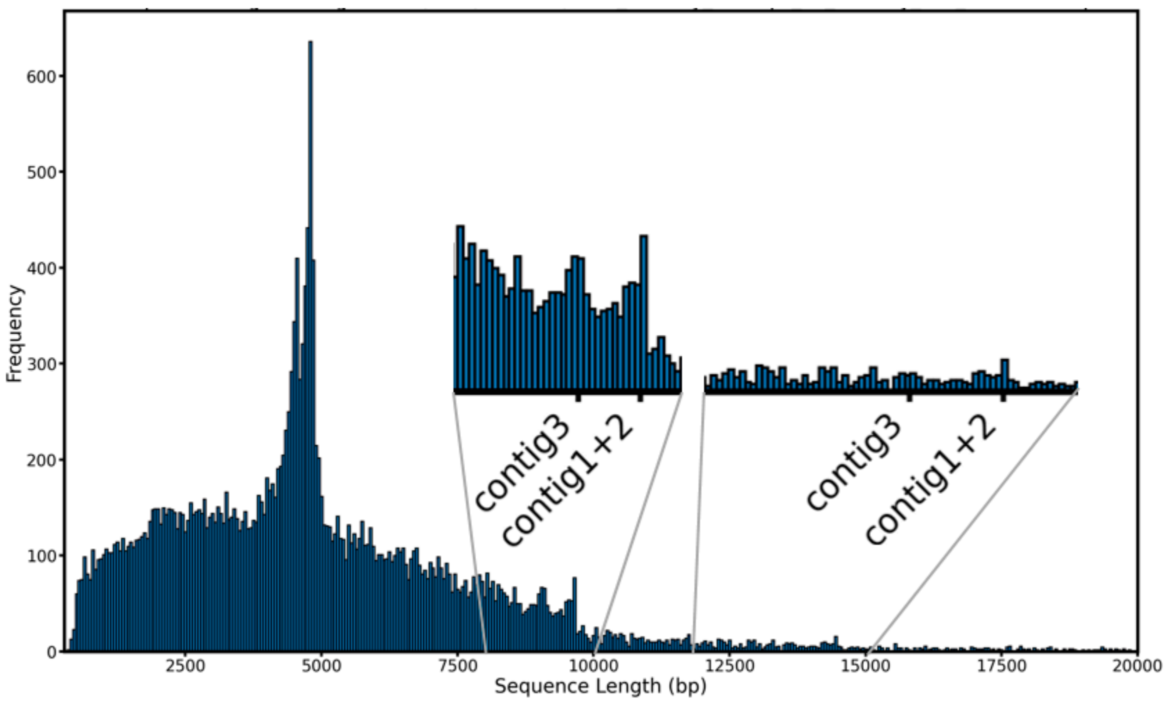

Supplementary Figure S8. Histogram of the sequence length distribution of BKPyV-specific reads. The reads unmapped to the monkey genome revealed a biphasic pattern, with a predominant peak comprising over 600 reads at 4820 bp along with an additional subvariant of 4815 bp and a secondary peak consisting of approximately 400 reads at 4540 bp. Notably, reads of increased sequence length (beyond 8000bp) appear to correspond to tandem repeats of the dominant sequence peaks, as illustrated in the insert.

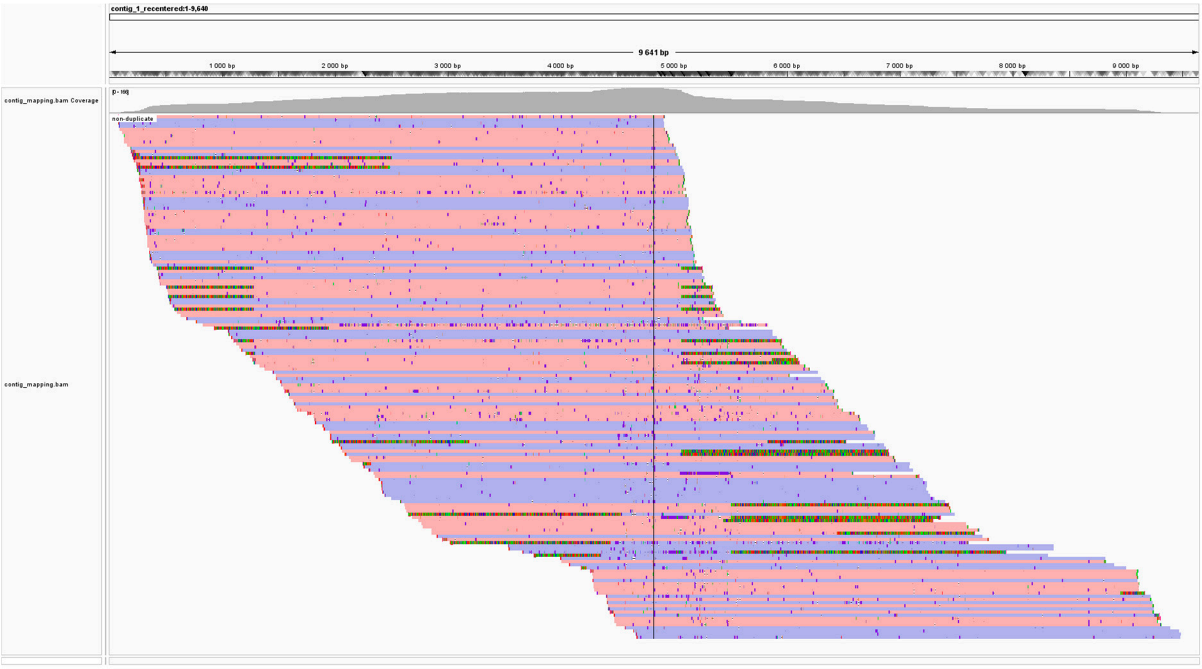

Supplementary Figure S9. IGV pile-up visualization of the read alignment for circular contig 1. The alignment of reads in IGV reveals overlapping sequences at the 5' and 3' ends of the contigs, indicating a circular genomic structure of BKPyV. Notably, the most frequent breakpoint within this circular configuration occurs at the NCCR.

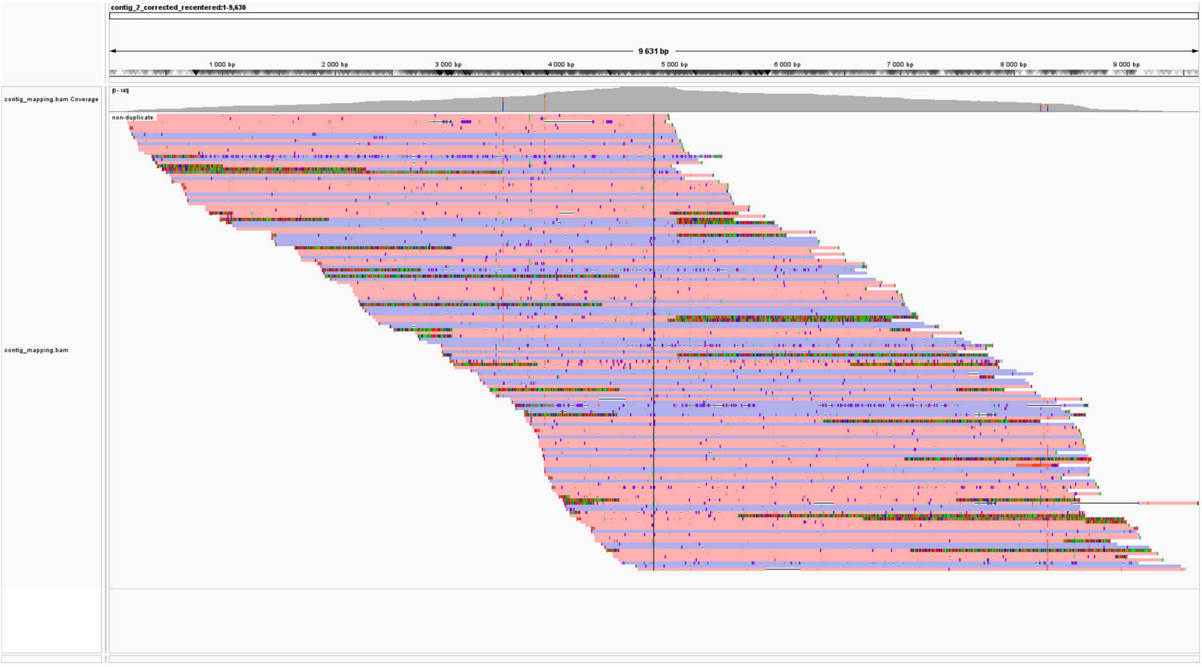

Supplementary Figure S10. IGV pile-up visualization of the read assembly for circular contig 2.

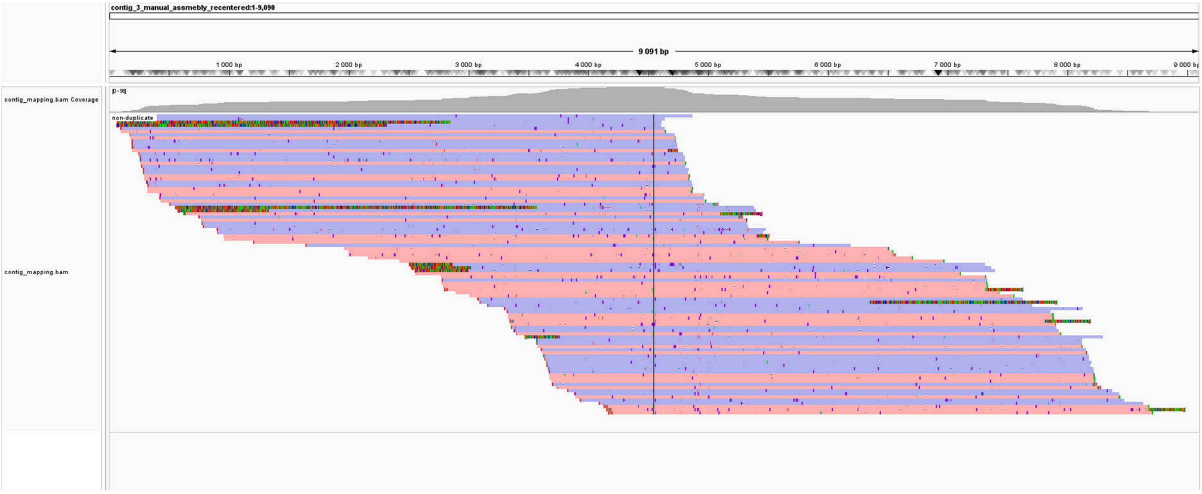

Supplementary Figure S11. IGV pile-up visualization of the read assembly for circular contig 3.
