# Supplementary material for: Establishment of a Stable BK Polyomavirus-Secreting Cell Line: Characterization of Viral Genome Integration and Replication Dynamics Through Comprehensive Analysis
Source: Int J Mol Sci. 2025 Jun 15;26(12):5745. doi: 10.3390/ijms26125745 (PMC12193623; doi:10.3390/ijms26125745)
Supplement: Supplementary file 1 [file ijms-26-05745-s001.zip › Supplementary file Clinical Isolate.pdf]

Supplementary Clinical Isolate.

Supplementary Table S1) ***Nuleotide substitutions in clinical isolate used for COSSA infection as identified by ONT sequening:*** NT-Position= nucleotide position within the canonical BKPyV genome, REF= reference nucleotide, ALT=substitution by alternate nucleotide.

NT-POSITION	REF	ALT
2	G	C
20	A	C
420	G	A
2826	G	A

DNA sequence ***in clinical isolate used for COSSA infection as identified by ONT sequening:***

*Nucleotide substitutions are highlighted in yellow, with the corresponding alternate nucleotides listed in Supplementary Table 1.1.*TGAGAGAAAGGGTGGAGGCAGAGGCGGCCTCGGCCTCTTATATATTATAAAAAAAAAGGCCACAGGGA
GGAGCTGCTAACCCATGGAATGTAGCCAAACCATGACCTCAGGAAGGAAAGTGCATGACTGGGCAGCCAGCC
AGTGGCAGTTAATAGTGAAACCCCGCCCCTAAAATTCTCAAATAAACACAAGAGGAAGTGGAAACTGGCCAAA
GGAGTGGAAAGCAGCCAGACAGACATGTTTTGCGAGCCTAGGAATCTTGGCCTTGTCCCCAGTTAAACTGGAC
AAAGGCCATGGTTCTGCGCCAGCTGTCACGACAAGCTTCTGTGAAAGTTAGTAAAACCTGGACTGGAACAAAA
AAAAGAGCTCAGAGGATTTTTATTTTTATTTTAGAGCTTTTGCTGGAATTTTGTAGAGGTGAAGACAGTGTAGA
CGGGAAAAACAAAAGTACCACTGCTTTACCTGCTGTAAAAGACTCTGTAAAAGACTCCTAGGTAAGTAATCCCT
TTTTTTTTGTATTTCCAGGTTGATGGGTGCTGCTCTAGCACTTTTGGGGGACCTAGTTGCCAGTGTATCTGAGGC
TGCTGCTGCCACAGGATTTTCAGTGGCTGAAATTGCTGCTGGGGAGGCTGCTGCTGCTATAGAAGTTCAAATT
GCATCCCTTGCTACTGTAGAGGGCATAACAAGTACCTCAGAGGCTATAGCTGCTATAGGCCTAACTCCTCAAAC
ATATGCTGTAATTGCTGGTGCTCCTGGGGCTATTGCTGGGTTTGCTGCTTTAATTCAAACTGTTACTGGTATTAG
TTCCTTGGCTCAAGTAGGGTATAGGTTTTTTAGTGATTGGGATCACAAAGTTTCCACTGTAGGCCTCTATCAGC
AATCAGGCATGGCTTTGGAATTGTTTAACCCAGATGAGTACTATGATATTTTGTTTCCTGGTGTAAATACTTTTG
TAAATAATATTCAATACCTTGATCCTAGGCATTGGGGTCCTTCCTTGTTTGCTACTATTTCCCAGGCTTTGTGGC
ATGTTATTAGGGATGATATACCTGCTATAACCTCACAAGAATTGCAAAGAAGAACAGAAAGATTTTTTAGAGA
CTCCTTGGCTAGATTTTTGGAGGAAACTACCTGGACAATTGTAAATGCCCCTATAAACTTTTATAATTATATTCA
AGAATATTATTCTGATCTTTCCCCTATTAGGCCCTCAATGGTTAGACAAGTGGCTGAAAGGGAAGGTACCCGTG
TACATTTTGGCCATACTTATAGTATAGATGATGCTGACAGTATAGAAGAAGTTACACAAAGAATGGACTTAAG
AAATCAACAAACTGTACATTCAGGAGAGTTTATAGAAAAAACTATTGCCCCAGGAGGTGCTAATCAAAGAACT
GCTCCTCAATGGATGTTGCCTTTACTTCTAGGCCTGTACGGGACTGTAACACCTGCTCTTGAAGCATATGAAGA
TGGCCCCAACCAAAAGAAAAGGAGAGTGTCCAGGGGCAGCTCCCAAAAAGCCAAAGGAACCCGTGCAAGTG
CCAAAACTACTAATAAAAGGAGGAGTAGAAGTTCTAGAAGTTAAAACTGGGGTAGATGCTATAACAGAGGTA
GAATGCTTCCTAAACCCAGAAATGGGGGATCCAGATGAAAACCTTAGGGGCTTTAGTCTAAAGCTAAGTGCTG
AAAATGACTTTAGCAGTGATAGCCCAGAAAGAAAAATGCTTCCCTGTTACAGCACAGCAAGAATTCCCCTCCCC
AATTTAAATGAGGACCTAACCTGTGGAAATCTACTGATGTGGGAGGCTGTAACTGTACAAACAGAGGTCATTG
GAATAACTAGCATGCTTAACCTTCATGCAGGGTCACAAAAAGTGCATGAGCATGGTGGAGGTAAACCTATTCA
AGGCAGTAATTTCCACTTCTTTGCTGTTGGTGGAGACCCCTTGGAAATGCAGGGAGTGCTAATGAATTACAGG
ACCAAGTACCCAGATGGTACTATAACCCCAAAAAACCCAACAGCCCAGTCCCAGGTAATGAATACTGACCATA
AGGCCTATTTGGACAAAAACAATGCTTATCCAGTTGAGTGCTGGGTTCCTGATCCCAGTAGAAATGAAAATACT
AGGTATTTTGGGACTTTCACAGGAGGGGAAAATGTTCCCCCAGTACTTCATGTGACCAACACAGCTACCACAG
TGTTGCTAGATGAACAGGGTGTGGGGCCTCTTTGTAAAGCTGATAGCCTGTATGTTTCAGCTGCTGATATTTGT

GGCCTGTTTACTAACAGCTCTGGAACACAACAGTGGAGAGGCCTTGCAAGATATTTTAAGATTCGCCTGAGAA
AAAGATCTGTAAAAAATCCTTACCCAATTTCCTTTTTGCTGAGTGACCTTATAAACAGGAGAACCCAGAGAGTG
GATGGGCAGCCTATGTATGGTATGGAATCCCAGGTAGAAGAGGTTAGGGTGTTTGATGGCACAGAAAGACTT
CCAGGGGACCCAGATATGATAAGATATATTGATAAACAAGGACAATTGCAAACCAAAATGCTTTAAACAGGTG
CTTTTATTGTACATATACATTTAATAAATGCTGCTTTTGTATAAGCCACTTTTAAGCTTGTGTTATTTTGGGGGTG
GTGTTTTAGGCCTTTTAAAACATTGAAAGCCTTTACACAAATGCAACTCTTGACTATGGGGGTCTGACCTTTGG
GAATCTTCAGCAGGGGCTGAAGTATCTGAGACTTGGGAAGAGCATTGTGATTGGGATTCAGTGCTTGATCCAT
GTCCAGAGTCTTCAGTTTCTGAATCTTCTTCTCTTGTGATATCAAGAATACATTTCCCCATGCATATATTATATTT
CATCCTTGAAAAAGTATACATACTTATCTCAGAATCCAGCCTTTCCTTCCATTCAACAATTCTAGATTGTATATCT
GTTGCAAAATCAGCTACAGGCCTAAACCAAATTAGCAGTAGCAACAAGGTCATTCCACTTTGTAAAATTCTTTTT
TCAAGTAAGAACTCTGAGTTTTGTAAGGATTTTCTTAAATATATTTTGGGTCTAAAATCTATCTGTCTTACAAAT
CTAGCCTGCAGGGTTTTAGGAACAGGATACTCATTCATTGTAACCAGGCCTGGTGGAAATATTTGGGTTCTTTT
GTTTAAATGTTTCTTTTCTAAATTAACCTTAACACTTCCATCTAAATAATCTCTCAAACTGTCTAAATTGTTTATTC
CATGTCCTGAAGGCAGATCCTTTGATTCAGCCCCAGTTCCTTTTACATCTTCAAAAACAACCATGTACTGATCTA
TAGCTACACCTAGTTCAAAGGTTAGCCTTTCCATGGGTAGGTTTACATTTAAGGCTTTACCTCCACACAAATCTA
ATAACCCTGCAGCTAGTGTTGTTTTTCCACTATCAATGGGACCTTTAAATAACCAGTATCTTCTTTTAGGTACATT
AAAAACAATACAGTGCAAAAAATCAAATATAACAGAATCCATTTTAGGTAGCAAACAGTGCAGCCAAGCAACA
CCTGCCATATATTGTTCCAGTACAGCATTTCCATGAGCTCCAAATATTAAATCCATTTTATCTAATATATGATTAA
ATCTTTCTGTTAGCATTTCTTCTCTGGTCATATGAAGGGTATCTACTCTTTTTTTAGCTAAAACTGTATCTACTGC
TTGCTGACAAATACTTTTTTGATTTTTACTTTCTGCAAAAATAGTAGCATTTGCAAAATGCTTTTCATGATACTTA
AAGTGATAAGGTTGGTCTTTTTTCTGACACTTTTTACACTCTTCTACATTGTATTGAAATTCTAAATACATACCCA
ATAATAAGAACACATCCTCACACTTTGTCTCTACTGCATACTCAGTAATTAATTTCCAAGACACCTGCTTTGTTTC
TTCAGGCTCTTCTGGGTTAAAATCATGCTCCTTTAAGCCCCCTTGAATGCTTTCTTCTATTGTATGGTATGGATCT
CTAGTTAAGGCACTATATAGTAAGTATTCCTTATTAACACCCTTACAAATTAAAAAACTAAAGGTACACAGCTTT
TGACAGAAATTATTAATTGCAGAAACTCTATGTCTATGTGGAGTTAAAAAGAATATAATATTATGCCCAGCACA
CATGTGTCTACTAATAAAAGTTACAGAATATTTTTCCATAAGTTTTTTATACAGAATTTGAGCTTTTTCTTTAGTA
GTATACACAGCAAAGCAGGCAAGGGTTCTATTACTAAATACAGCTTGACTAAGAAACTGGTGTAGATCAGAAG
GAAAGTCTTTAGGGTCTTCTACCTTTCTCTTTTTCTTGGGTGGTGTGGAGTGTTGAGAATCTGCTGTTGCTTCTT
CATCACTGGCAAACATATCTTCATGGCAAAATAAATCTTCATCCCATTTTTCATTAAAGGAGCTCCACCAGGACT
CCCACTCTTCTGTTCCATAGGTTGGCACCTATAAAAAAAATAATTACTTAGGGCCTTTAAATATTTTCTTATTTAT
CTAAATATAAGTTAGTTACCTTAAAGCTTTAGATCTCTGAAGGGAGTTTCTCCAATTATTTGGACCCACCATTGC
AGAGTTTCTTCAGTTAGGTCTAAGCCAAACCACTGTGTGAAGCAGTCAATGCAGTAGCAATCTATCCAAACCAA
GGGCTCTTTTCTTAAAAATTTTCTATTTAAATGCCTTAATCTAAGCTGACATAGCATGCAAGGGCAGTGCACAG
AAGGCTTTTTGGAACAAATAGGCCAATCCTTGCAGTACAGGGTATCTGGGCAAAGAGGAAAATCAGCACAAA
CCTCTGAGCTACTCCAGGTTCCAAAATCAGGCTGATGAGCTACCTTTACATCCTGCTCCATTTTTTTATATAAAG
TATTCATTCTCTTCATTTTATCCTCGTCGCCCCCTTTGTCAGGGTGAAATTCCTTACACTTCCTTAAATAGGCTTTT
CTCATTAAGGGAAGGTTTCCCCAGGCAGCTCTTTCAAGGCCTAAAAGGTCCATGAGCTCCATGGATTCTTCCCT
GTTAAGCACTTTATCCATTTTTGCAAAAATTGCAAAAGAATAGGGATTTCCCCAAATATTTTTGCTAGGCCTCAG
AAAAAGCCTCCACACCCTTACTACT
