# Supplementary material for: Establishment of a Stable BK Polyomavirus-Secreting Cell Line: Characterization of Viral Genome Integration and Replication Dynamics Through Comprehensive Analysis
Source: Int J Mol Sci. 2025 Jun 15;26(12):5745. doi: 10.3390/ijms26125745 (PMC12193623; doi:10.3390/ijms26125745)
Supplement: Supplementary file 1 [file ijms-26-05745-s001.zip › Supplementary File.pdf]

Supplementary File

Contig 4 as shown in figure 3A)

TATAATTATAAAAGTTTATAGGGGCATTTACAATTGTCCAGGTAGTTTCCTCCAAAAATCTAGCCAAGGAGTCTCTAAAAAATCTTTCTGTTCTTCTTTGCAAT
TCTTGTGAGGTTATAGCAGGTATATCATCCCTAATAACATGCCACAAAGCCTGGGAAATAGTAGCAAACAAGGAAGGACCCCAATGCCTAGGATCAAGGT
ATTGAATATTATTTACAAAAGTATTTACACCAGGAAACAAAATATCATAGTACTCATCTGGGTTAAACAATTCCAAAGCCATGCCTGATTGCTGATAGAGGC
CTACAGTGGAAACTTTGTGATCCCAATCACTAAAAAACCTATACCCTACTTGAGCCAAGGAACTAATACCAGTAACAGTTTGAATTAAAGCAGCAAACCCAG
CAATAGCCCCAGGAGCACCAGCAATTACAGCATATGTTTGAGGAGTTAGGCCTATAGCAGCTATAGCCTCTGAGGTACTTGTTATGCCCTCTACAGTAGCA
AGGGATGCAATTTGAACTTCTATAGCAGCAGCAGCCTCCCCAGCAGCAATTTCAGCCACTGAAAATCCTGTGGCAGCAGCAGCCTCAGATACACTGGCAAC
TAGGTCCCCCAAAAGTGCTAGAGCAGCACCCATCAACCTGGAAATACAAAAAAAAAGGGATTACTTACCTAGGAGTCTTTTACAGAGTCTTTTACAGCAGG
TAAAGCAGTGGTACTTTTGTTTTTCCCGTCTACACTGTCTTCACCTCTACAAAATTCCAGCAAAAGCTCTAAAATAAAAATAAAAATCCTCTGAGCTCTTTTTT
TTGTTCCAGTCCAGGTTTTACTAACTTTCACAGAAGCTTGTCGTGACAGCTGGCGCAGAACCATGGCCTTTGTCCAGTTTAACTGGGGACAAGGCCAAGATT
CCTAGGCTCGCAAAACATGTCTGTCTGGCTGCTTTCCACTCCTTTGGCCAGTTTCCACTTCCTCTTGTGTTTATTTGAGAATTTTAGGGGCGGGGTTTCACTAT
TAACTGCCACTGGCTGGCTGCCCAGTCATGCACTTTCCTTCCTGAGGTCATGGTTTGGCTACATTCCATGGGTTAGCAGCTCCTCCCTGTGGCCTTTTTTTTTA
TAATATATAAGAGGCCGAGGCCGCCTCGGCCTCCACCCTTTCTCAGAAGTAGTAAGGGTGTGGAGGCTTTTTCTGAGGCCTAGCAAAAATATTTGGGGAAA
TCCCTATTCTTTTGCAATTTTTGCAAAAATGGATAAAGTGCTTAACAGGGAAGAATCCATGGAGCTCATGGACCTTTTAGGCCTTGAAAGAGCTGCCTGGG
GAAACCTTCCCTTAATGAGAAAAGCCTATTTAAGGAAGTGTAAGGAATTTCACCCTGACAAAGGGGGCGACGAGGATAAAATGAAGAGAATGAATACTTT
ATATAAAAAAATGGAGCAGGATGTAAAGGTAGCTCATCAGCCTGATTTTGGAACCTGGAGTAGCTCAGAGGTTTGTGCTGATTTTCCTCTTTGCCCAGATA
CCCTGTACTGCAAGGATTGGCCTATTTGTTCCAAAAAGCCTTCTGTGCACTGCCCTTGCATGCTATGTCAGCTTAGATTAAGGCATTTAAATAGAAAATTTTT
AAGAAAAGAGCCCTTGGTTTGGATAGATTGCTACTGCATTGACTGCTTCACACAGTGGTTTGGCTTAGACCTAACTGAAGAAACTCTGCAATGGTGGGTCC
AAATAATTGGAGAAACTCCCTTCAGAGATCTAAAGCTTTAAGGTAACTAACTTATATTTAGATAAATAAGAAAATATTTAAAGGCCCTAAGTAATTATTTTTT
TTATAGGTGCCAACCTATGGAACAGAAGAGTGGGAGTCCTGGTGGAGCTCCTTTAATGAAAAATGGGATGAAGATTTATTTTGCCATGAAGATATGTTTGC
CAGTGATGAAGAAGCAACAGCAGATTCTCAACACTCCACACCACCCAAGAAAAAGAGAAAGGTAGAAGACCCTAAAGACTTTCCTTCTGATCTCTGTCTGG
CTGCTTTCCACTCCTTTGGCCAGTTTCCACTTCCTCTTGTGTTTATTTGAGAATTTTAGGGGCGGGGTTTCACTATTAACTGCCACTGGCTGGCTGCCCAGTCA
TGCACTTTCCTTCCTGAGGTCATGGTTTGGCTACATTCCATGGGTTAGCAGCTCCTCCCTGTGGCCTTTTTTTTTATAATATATAAGAGGCCGAGGCCGCCTC
GGCCTCCACCCTTTCTCAGAAGTAGTAAGGGTGTGGAGGCTTTTTCTGAGGCCTAGCAAAAATATTTGGGGAAATCCCTATTCTTTTGCAATTTTTGCAAAA
ATGGATAAAGTGCTTAACAGGGAAGAATCCATGGAGCTCATGGACCTTTTAGGCCTTGAAAGAGCTGCCTGGGGAAACCTTCCCTTAATGAGAAAAGCCT
ATTTAAGGAAGTGTAAGGAATTTCACCCTGACAAAGGGGGCGACGAGGATAAAATGAAGAGAATGAATACTTTATATAAAAAAATGAAGCAGGATGTAA
AGGTAGCTCATCAGCCTGATTTTGGAACCTGGAGTAGCTCAGAGGTTTGTGCTGATTTTCCTCTTTGCCCAGATACCCTGTACTGCAAGGATTGGCCTATTT
GTTCCAAAAAGCCTTCTGTGCACTGCCCTTGCATGCTATGTCAGCTTAGATTAAGGCATTTAAATAGAAAATTTTTAAGAAAAGAGCCCTTGGTTTGGATAG
ATTGCTACTGCATTGACTGCTTCACACAGTGGTTTGGCTTAGACCTAACTGAAGAAACTCTGCAATGGTGGGTCCAAATAATTGGAGAAACTCCCTTCAGAG
ATCTAAAGCTTTAAGGTAACTAACTTATATTTAGATAAATAAGAAAATATTTAAAGGCCCTAAGTAATTATTTTTAAATATATTTAAGAAAATCCTTACAAAA
CTCAGAGTTCTTACTTGAAAAAAGAATTTTACAAAGTGGAATGACCTTGTTGCTACTGCTAATTTGGTTTAGGCCTGTAGCTGATTTTGCAACAGATATACA
ATCTAGAATTGTTGAATGGAAGGAAAGGCTGGATTCTGAGATAAGTATGTATACTTTTTCAAGGATGAAATATAATATATGCATGGGGAAATGTATTCTTG
ATATCACAAGAGAAGAAGATTCAGAAACTGAAGACTCTGGACATGGATCAAGCACTGAATCCCAATCACAATGCTCTTCCCAAGTCTGAGATACTTCAGCC
CCTGCTGAAGATTCCCAAAGGTCAGACCCCCATAGTCAAGAGTTGCATTTGTGTAAAGGCTTTCAATGTTTTAAAAGGCCTAAAACACCACCCCCAAAATAA
CACAAGCTTAAAAGTGGCTTATACAAAAGCAGCATTTATTAAATGTATATGTACAATAAAAGCACCTGTTTAAAGCATTTTGGTTTGCAATTGTCCTTGTTTA
TCAATATATCTTATCATATCTGGGTCCCCTGGAAGTCTTTCTGTGCCATCAAACACCCTAACCTCTTCTACCTGGGATTCCATACCATACATAGGCTGCCCATC
CACTCTCTGGGTTCTCCTGTTTATAAGGTCACTCAGCAAAAAGGAAATTGGGTAAGGATTTTTTACAGATCTTTTTCTCAGGCGAATCTTAAAATATCTTGCA
AGGCCTCTCCACTGTTGTGTTCCAGAGCTGTTAGTAAACAGGCCACAAATATCAGCAGCTGAAACATACAGGCTATCAGCTTTACAAAGAGGCCCCACACC
CTGTTCATCTAGCAACACTGTGGTAGCTGTGTTGGTCACATGAAGTACTGGGGGAACATTTTCCCCTCCTGTGAAAGTCCCAAAATACCTAGTATTTTCATTT
CTACTGGGATCAGGAACCCAGCACTCAACTGGATAAGCATTGTTTTTGTCCAAATAGGCCTTATGGTCAGTATTCATTACCTGGGACTGGGCTGTTGGGTTT
TTTGGGGTTATAGTACCATCTGGGTACTTGGTCCTGTAATTCATTAGCACTCCCTGCATTTCCAAGGGGTCTCCACCAACAGCAAAGAAGTGGAAATTACTG
CCTTGAATAGGTTTACCTCCACCATGCTCATGCACTTTTTGTGACCCTGCATGAAGGTTAAGCATGCTAGTTATTCCAATGACCTCTGTTTGTACAGTTACAG
CCTCCCACATCAGTAGATTTCCACAGGTTAGGTCCTCATTTAAATTGGGGAGGGGAATTCTTGCTGTGCTGTAACAGGGAAGCATTTTTCTTTCTGGGCTAT
CACTGCTAAAGTCATTTTCAGCACTTAGCTTTAGACTAAAGCCCCTAAGGTTTTCATCTGGATCCCCCATTTCTGGGTTTAGGAAGCATTCTACCTCTGTTATA
GCATCTACCCCAGTTTTAACTTCTAGAACTTCTACTCCTCCTTTTATTAGTAGTTTTGGCACTTGCACGGGTTCCTTTGGCTTTTTGGGAGCTGCCCCTGGACA
CTCTCCTTTTCTTTTGGTTGGGGCCATCTTCATATGCTTCAAGAGCAGGTGTTACAGTCCCGTACAGGCCTAGAAGTAAAGGCAACATCCATTGAGGAGCAG
TTCTTTGATTAGCACCTCCTGGGGCAATAGTTTTTTCTATAAACTCTCCTGAATGTACAGTTTGTTGATTTCTTAAGTCCATTCTTTGTGTAACTTCTTCTATAC
TGTCAGCATCATCTATACTATAAGTATGGCCAAAATGTACACGGGTACCTTCCCTTTCAGCCACTTGTCTAACCATTGAGGGCCTAATAGGGGAAAGATCAG
AATAATATTCTTGAATATAATTATAAAAGTTTATAGGGGCATTTACAATTGTCCAGGTAGTTTCCTCCAAAAATCTAGCCAAGGAGTCTCTAAAAAATCTTTC
TGTTCTTCTTTGCAATTCTTGTGAGGTTATAGCAGGTATATCATCCCTAATAACATGCCACAAAGCCTGGGAAATAGTAGCAAACAAGGAAGGACCCCAATG
CCTAGGATCAAGGTATTGAATATTATTTACAAAAGTATTTACACCAGGAAACAAAATATCATAGTACTCATCTGGGTTAAACAATTCCAAAGCCATGCCTGA
TTGCTGATAGAGGCCTACAGTGGAAACTTTGTGATCCCAATCACTAAAAAACCTATACCCTACTTGAGCCAAGGAACTAATACCAGTAACAGTTTGAATTAA
AGCAGCAAACCCAGCAATAGCCCCAGGAGCACCAGCAATTACAGCATATGTTTGAGGAGTTAGGCCTATAGCAGCTATAGCCTCTGAGGTACTTGTTATGC
CCTCTACAGTAGCAAGGGATGCAATTTGAACTTCTATAGCAGCAGCAGCCTCCCCAGCAGCAATTTCAGCCACTGAAAATCCTGTGGCAGCAGCAGCCTCA
GATACACTGGCAACTAGGTCCCCCAAAAGTGCTAGAGCAGCACCCATCAACCTGGAAATACAAAAAAAAAGGGATTACTTACCTAGGAGTCTTTTACAGAG
TCTTTTACAGCAGGTAAAGCAGTGGTACTTTTGTTTTTCCCGTCTACACTGTCTTCACCTCTACAAAATTCCAGCAAAAGCTCTAAAATAAAAATAAAAATCCT
CTGAGCTCTTTTTTTTGTTCCAGTCCAGGTTTTACTAACTTTCACAGAAGCTTGTCGTGACAGCTGGCGCAGAACCATGGCCTTTGTCCAGTTTAACTGGGGA
CAAGGCCAAGATTCCTAGGCTCGCAAAACATGTCTGTCTGGCTGCTTTCCACTCCTTTGGCCAGTTTCCACTTCCTCTTGTGTTTATTTGAGAATTTTAGGGG
CGGGGTTTCACTATTAACTGCCACTGGCTGGCTGCCCAGTCATGCACTTTCCTTCCTGAGGTCATGGTTTGGCTACATTCCATGGGTTAGCAGCTCCTCCCTG
TGGCCTTTTTTTTTATAATATATAAGAGGCCGAGGCCGCCTCGGCCTCCACCCTTTCTCAGAAGTAGTAAGGGTGTGGAGGCTTTTTCTGAGGCCTAGCAAA
AATATTTGGGGAAATCCCTATTCTTTTGCAATTTTTGCAAAAATGGATAAAGTGCTTAACAGGGAAGAATCCATGGAGCTCATGGACCTTTTAGGCCTTGAA
AGAGCTGCCTGGGGAAACCTTCCCTTAATGAGAAAAGCCTATTTAAGGAAGTGTAAGGAATTTCACCCTGACAAAGGGGGCGACGAGGATAAAATGAAG

AGAATGAATACTTTATATAAAAAAATGGAGCAGGATGTAAAGGTAGCTCATCAGCCTGATTTTGGAACCTGGAGTAGCTCAGAGGTTTGTGCTGATTTTCC
TCTTTGCCCAGATACCCTGTACTGCAAGGATTGGCCTATTTGTTCCAAAAAGCCTTCTGTGCACTGCCCTTGCATGCTATGTCAGCTTAGATTAAGGCATTTA
AATAGAAAATTTTTAAGAAAAGAGCCCTTGGTTTGGATAGATTGCTACTGCATTGACTGCTTCACACAGTGGTTTGGCTTAGACCTAACTGAAGAAACTCTG
CAATGGTGGGTCCAAATAATTGGAGAAACTCCCTTCAGAGATCTAAAGCTTTAAGGTAACTAACTTATATTTAGATAAATAAGAAAATATTTAAAGGCCCTA
AGTAATTATTTTTTTTATAGGTGCCAACCTATGGAACAGAAGAGTGGGAGTCCTGGTGGAGCTCCTTTAATGAAAAATGGGATGAAGATTTATTTTGCCAT
GAAGATATGTTTGCCAGTGATGAAGAAGCAACAGCAGATTCTCAACACTCCACACCACCCAAGAAAAAGAGAAAGGTAGAAGACCCTAAAGACTTTCCTTC
TGATCTCTGTCTGGCTGCTTTCCACTCCTTTGGCCAGTTTCCACTTCCTCTTGTGTTTATTTGAGAATTTTAGGGGCGGGGTTTCACTATTAACTGCCACTGGC
TGGCTGCCCAGTCATGCACTTTCCTTCCTGAGGTCATGGTTTGGCTACATTCCATGGGTTAGCAGCTCCTCCCTGTGGCCTTTTTTTTTATAATATATAAGAG
GCCGAGGCCGCCTCGGCCTCCACCCTTTCTCAGAAGTAGTAAGGGTGTGGAGGCTTTTTCTGAGGCCTAGCAAAAATATTTGGGGAAATCCCTATTCTTTTG
CAATTTTTGCAAAAATGGATAAAGTGCTTAACAGGGAAGAATCCATGGAGCTCATGGACCTTTTAGGCCTTGAAAGAGCTGCCTGGGGAAACCTTCCCTTA
ATGAGAAAAGCCTATTTAAGGAAGTGTAAGGAATTTCACCCTGACAAAGGGGGCGACGAGGATAAAATGAAGAGAATGAATACTTTATATAAAAAAATG
AAGCAGGATGTAAAGGTAGCTCATCAGCCTGATTTTGGAACCTGGAGTAGCTCAGAGGTTTGTGCTGATTTTCCTCTTTGCCCAGATACCCTGTACTGCAAG
GATTGGCCTATTTGTTCCAAAAAGCCTTCTGTGCACTGCCCTTGCATGCTATGTCAGCTTAGATTAAGGCATTTAAATAGAAAATTTTTAAGAAAAGAGCCCT
TGGTTTGGATAGATTGCTACTGCATTGACTGCTTCACACAGTGGTTTGGCTTAGACCTAACTGAAGAAACTCTGCAATGGTGGGTCCAAATAATTGGAGAA
ACTCCCTTCAGAGATCTAAAGCTTTAAGGTAACTAACTTATATTTAGATAAATAAGAAAATATTTAAAGGCCCTAAGTAATTATTTTTAAATATATTTAAGAA
AATCCTTACAAAACTCAGAGTTCTTACTTGAAAAAAGAATTTTACAAAGTGGAATGACCTTGTTGCTACTGCTAATTTGGTTTAGGCCTGTAGCTGATTTTGC
AACAGATATACAATCTAGAATTGTTGAATGGAAGGAAAGGCTGGATTCTGAGATAAGTATGTATACTTTTTCAAGGATGAAATATAATATATGCATGGGGA
AATGTATTCTTGATATCACAAGAGAAGAAGATTCAGAAACTGAAGACTCTGGACATGGATCAAGCACTGAATCCCAATCACAATGCTCTTCCCAAGTCTGA
GATACTTCAGCCCCTGCTGAAGATTCCCAAAGGTCAGACCCCCATAGTCAAGAGTTGCATTTGTGTAAAGGCTTTCAATGTTTTAAAAGGCCTAAAACACCA
CCCCCAAAATAACACAAGCTTAAAAGTGGCTTATACAAAAGCAGCATTTATTAAATGTATATGTACAATAAAAGCACCTGTTTAAAGCATTTTGGTTTGCAA
TTGTCCTTGTTTATCAATATATCTTATCATATCTGGGTCCCCTGGAAGTCTTTCTGTGCCATCAAACACCCTAACCTCTTCTACCTGGGATTCCATACCATACAT
AGGCTGCCCATCCACTCTCTGGGTTCTCCTGTTTATAAGGTCACTCAGCAAAAAGGAAATTGGGTAAGGATTTTTTACAGATCTTTTTCTCAGGCGAATCTTA
AAATATCTTGCAAGGCCTCTCCACTGTTGTGTTCCAGAGCTGTTAGTAAACAGGCCACAAATATCAGCAGCTGAAACATACAGGCTATCAGCTTTACAAAGA
GGCCCCACACCCTGTTCATCTAGCAACACTGTGGTAGCTGTGTTGGTCACATGAAGTACTGGGGGAACATTTTCCCCTCCTGTGAAAGTCCCAAAATACCTA
GTATTTTCATTTCTACTGGGATCAGGAACCCAGCACTCAACTGGATAAGCATTGTTTTTGTCCAAATAGGCCTTATGGTCAGTATTCATTACCTGGGACTGG
GCTGTTGGGTTTTTTGGGGTTATAGTACCATCTGGGTACTTGGTCCTGTAATTCATTAGCACTCCCTGCATTTCCAAGGGGTCTCCACCAACAGCAAAGAAG
TGGAAATTACTGCCTTGAATAGGTTTACCTCCACCATGCTCATGCACTTTTTGTGACCCTGCATGAAGGTTAAGCATGCTAGTTATTCCAATGACCTCTGTTT
GTACAGTTACAGCCTCCCACATCAGTAGATTTCCACAGGTTAGGTCCTCATTTAAATTGGGGAGGGGAATTCTTGCTGTGCTGTAACAGGGAAGCATTTTTC
TTTCTGGGCTATCACTGCTAAAGTCATTTTCAGCACTTAGCTTTAGACTAAAGCCCCTAAGGTTTTCATCTGGATCCCCCATTTCTGGGTTTAGGAAGCATTCT
ACCTCTGTTATAGCATCTACCCCAGTTTTAACTTCTAGAACTTCTACTCCTCCTTTTATTAGTAGTTTTGGCACTTGCACGGGTTCCTTTGGCTTTTTGGGAGCT
GCCCCTGGACACTCTCCTTTTCTTTTGGTTGGGGCCATCTTCATATGCTTCAAGAGCAGGTGTTACAGTCCCGTACAGGCCTAGAAGTAAAGGCAACATCCA
TTGAGGAGCAGTTCTTTGATTAGCACCTCCTGGGGCAATAGTTTTTTCTATAAACTCTCCTGAATGTACAGTTTGTTGATTTCTTAAGTCCATTCTTTGTGTAA
CTTCTTCTATACTGTCAGCATCATCTATACTATAAGTATGGCCAAAATGTACACGGGTACCTTCCCTTTCAGCCACTTGTCTAACCATTGAGGGCCTAATAGG
GGAAAGATCAGAATAATATTCTTGAATATAATTATAAAAGTTTATAGGGGCATTTACAATTGTCCAGGTAGTTTCCTCCAAAAATCTAGCCAAGGAGTCTCT
AAAAAATCTTTCTGTTCTTCTTTGCAATTCTTGTGAGGTTATAGCAGGTATATCATCCCTAATAACATGCCACAAAGCCTGGGAAATAGTAGCAAACAAGGA
AGGACCCCAATGCCTAGGATCAAGGTATTGAATATTATTTACAAAAGTATTTACACCAGGAAACAAAATATCATAGTACTCATCTGGGTTAAACAATTCCAA
AGCCATGCCTGATTGCTGATAGAGGCCTACAGTGGAAACTTTGTGATCCCAATCACTAAAAAACCTATACCCTACTTGAGCCAAGGAACTAATACCAGTAAC
AGTTTGAATTAAAGCAGCAAACCCAGCAATAGCCCCAGGAGCACCAGCAATTACAGCATATGTTTGAGGAGTTAGGCCTATAGCAGCTATAGCCTCTGAG
GTACTTGTTATGCCCTCTACAGTAGCAAGGGATGCAATTTGAACTTCTATAGCAGCAGCAGCCTCCCCAGCAGCAATTTCAGCCACTGAAAATCCTGTGGCA
GCAGCAGCCTCAGATACACTGGCAACTAGGTCCCCCAAAAGTGCTAGAGCAGCACCCATCAACCTGGAAATACAAAAAAAAAGGGATTACTTACCTAGGA
GTCTTTTACAGAGTCTTTTACAGCAGGTAAAGCAGTGGTACTTTTGTTTTTCCCGTCTACACTGTCTTCACCTCTACAAAATTCCAGCAAAAGCTCTAAAATAA
AAATAAAAATCCTCTGAGCTCTTTTTTTTGTTCCAGTCCAGGTTTTACTAACTTTCACAGAAGCTTGTCGTGACAGCTGGCGCAGAACCATGGCCTTTGTCCA
GTTTAACTGGGGACAAGGCCAAGATTCCTAGGCTCGCAAAACATGTCTGTCTGGCTGCTTTCCACTCCTTTGGCCAGTTTCCACTTCCTCTTGTGTTTATTTG
AGAATTTTAGGGGCGGGGTTTCACTATTAACTGCCACTGGCTGGCTGCCCAGTCATGCACTTTCCTTCCTGAGGTCATGGTTTGGCTACATTCCATGGGTTA
GCAGCTCCTCCCTGTGGCCTTTTTTTTTATAATATATAAGAGGCCGAGGCCGCCTCGGCCTCCACCCTTTCTCAGAAGTAGTAAGGGTGTGGAGGCTTTTTCT
GAGGCCTAGCAAAAATATTTGGGGAAATCCCTATTCTTTTGCAATTTTTGCAAAAATGGATAAAGTGCTTAACAGGGAAGAATCCATGGAGCTCATGGACC
TTTTAGGCCTTGAAAGAGCTGCCTGGGGAAACCTTCCCTTAATGAGAAAAGCCTATTTAAGGAAGTGTAAGGAATTTCACCCTGACAAAGGGGGCGACGA
GGATAAAATGAAGAGAATGAATACTTTATATAAAAAAATGGAGCAGGATGTAAAGGTAGCTCATCAGCCTGATTTTGGAACCTGGAGTAGCTCAGAGGTT
TGTGCTGATTTTCCTCTTTGCCCAGATACCCTGTACTGCAAGGATTGGCCTATTTGTTCCAAAAAGCCTTCTGTGCACTGCCCTTGCATGCTATGTCAGCTTA
GATTAAGGCATTTAAATAGAAAATTTTTAAGAAAAGAGCCCTTGGTTTGGATAGATTGCTACTGCATTGACTGCTTCACACAGTGGTTTGGCTTAGACCTAA
CTGAAGAAACTCTGCAATGGTGGGTCCAAATAATTGGAGAAACTCCCTTCAGAGATCTAAAGCTTTAAGGTAACTAACTTATATTTAGATAAATAAGAAAA
TATTTAAAGGCCCTAAGTAATTATTTTTTTTATAGGTGCCAACCTATGGAACAGAAGAGTGGGAGTCCTGGTGGAGCTCCTTTAATGAAAAATGGGATGAA
GATTTATTTTGCCATGAAGATATGTTTGCCAGTGATGAAGAAGCAACAGCAGATTCTCAACACTCCACACCACCCAAGAAAAAGAGAAAGGTAGAAGACCC
TAAAGACTTTCCTTCTGATCTCTGTCTGGCTGCTTTCCACTCCTTTGGCCAGTTTCCACTTCCTCTTGTGTTTATTTGAGAATTTTAGGGGCGGGGTTTCACTAT
TAACTGCCACTGGCTGGCTGCCCAGTCATGCACTTTCCTTCCTGAGGTCATGGTTTGGCTACATTCCATGGGTTAGCAGCTCCTCCCTGTGGCCTTTTTTTTTA
TAATATATAAGAGGCCGAGGCCGCCTCGGCCTCCACCCTTTCTCAGAAGTAGTAAGGGTGTGGAGGCTTTTTCTGAGGCCTAGCAAAAATATTTGGGGAAA
TCCCTATTCTTTTGCAATTTTTGCAAAAATGGATAAAGTGCTTAACAGGGAAGAATCCATGGAGCTCATGGACCTTTTAGGCCTTGAAAGAGCTGCCTGGG
GAAACCTTCCCTTAATGAGAAAAGCCTATTTAAGGAAGTGTAAGGAATTTCACCCTGACAAAGGGGGCGACGAGGATAAAATGAAGAGAATGAATACTTT
ATATAAAAAAATGAAGCAGGATGTAAAGGTAGCTCATCAGCCTGATTTTGGAACCTGGAGTAGCTCAGAGGTTTGTGCTGATTTTCCTCTTTGCCCAGATA
CCCTGTACTGCAAGGATTGGCCTATTTGTTCCAAAAAGCCTTCTGTGCACTGCCCTTGCATGCTATGTCAGCTTAGATTAAGGCATTTAAATAGAAAATTTTT
AAGAAAAGAGCCCTTGGTTTGGATAGATTGCTACTGCATTGACTGCTTCACACAGTGGTTTGGCTTAGACCTAACTGAAGAAACTCTGCAATGGTGGGTCC
AAATAATTGGAGAAACTCCCTTCAGAGATCTAAAGCTTTAAGGTAACTAACTTATATTTAGATAAATAAGAAAATATTTAAAGGCCCTAAGTAATTATTTTTA
AATATATTTAAGAAAATCCTTACAAAACTCAGAGTTCTTACTTGAAAAAAGAATTTTACAAAGTGGAATGACCTTGTTGCTACTGCTAATTTGGTTTAGGCCT
GTAGCTGATTTTGCAACAGATATACAATCTAGAATTGTTGAATGGAAGGAAAGGCTGGATTCTGAGATAAGTATGTATACTTTTTCAAGGATGAAATATAA
TATATGCATGGGGAAATGTATTCTTGATATCACAAGAGAAGAAGATTCAGAAACTGAAGACTCTGGACATGGATCAAGCACTGAATCCCAATCACAATGCT
CTTCCCAAGTCTGAGATACTTCAGCCCCTGCTGAAGATTCCCAAAGGTCAGACCCCCATAGTCAAGAGTTGCATTTGTGTAAAGGCTTTCAATGTTTTAAAA
GGCCTAAAACACCACCCCCAAAATAACACAAGCTTAAAAGTGGCTTATACAAAAGCAGCATTTATTAAATGTATATGTACAATAAAAGCACCTGTTTAAAGC

ATTTTGGTTTGCAATTGTCCTTGTTTATCAATATATCTTATCATATCTGGGTCCCCTGGAAGTCTTTCTGTGCCATCAAACACCCTAACCTCTTCTACCTGGGAT
TCCATACCATACATAGGCTGCCCATCCACTCTCTGGGTTCTCCTGTTTATAAGGTCACTCAGCAAAAAGGAAATTGGGTAAGGATTTTTTACAGATCTTTTTC
TCAGGCGAATCTTAAAATATCTTGCAAGGCCTCTCCACTGTTGTGTTCCAGAGCTGTTAGTAAACAGGCCACAAATATCAGCAGCTGAAACATACAGGCTAT
CAGCTTTACAAAGAGGCCCCACACCCTGTTCATCTAGCAACACTGTGGTAGCTGTGTTGGTCACATGAAGTACTGGGGGAACATTTTCCCCTCCTGTGAAAG
TCCCAAAATACCTAGTATTTTCATTTCTACTGGGATCAGGAACCCAGCACTCAACTGGATAAGCATTGTTTTTGTCCAAATAGGCCTTATGGTCAGTATTCAT
TACCTGGGACTGGGCTGTTGGGTTTTTTGGGGTTATAGTACCATCTGGGTACTTGGTCCTGTAATTCATTAGCACTCCCTGCATTTCCAAGGGGTCTCCACC
AACAGCAAAGAAGTGGAAATTACTGCCTTGAATAGGTTTACCTCCACCATGCTCATGCACTTTTTGTGACCCTGCATGAAGGTTAAGCATGCTAGTTATTCC
AATGACCTCTGTTTGTACAGTTACAGCCTCCCACATCAGTAGATTTCCACAGGTTAGGTCCTCATTTAAATTGGGGAGGGGAATTCTTGCTGTGCTGTAACA
GGGAAGCATTTTTCTTTCTGGGCTATCACTGCTAAAGTCATTTTCAGCACTTAGCTTTAGACTAAAGCCCCTAAGGTTTTCATCTGGATCCCCCATTTCTGGG
TTTAGGAAGCATTCTACCTCTGTTATAGCATCTACCCCAGTTTTAACTTCTAGAACTTCTACTCCTCCTTTTATTAGTAGTTTTGGCACTTGCACGGGTTCCTTT
GGCTTTTTGGGAGCTGCCCCTGGACACTCTCCTTTTCTTTTGGTTGGGGCCATCTTCATATGCTTCAAGAGCAGGTGTTACAGTCCCGTACAGGCCTAGAAG
TAAAGGCAACATCCATTGAGGAGCAGTTCTTTGATTAGCACCTCCTGGGGCAATAGTTTTTTCTATAAACTCTCCTGAATGTACAGTTTGTTGATTTCTTAAG
TCCATTCTTTGTGTAACTTCTTCTATACTGTCAGCATCATCTATACTATAAGTATGGCCAAAATGTACACGGGTACCTTCCCTTTCAGCCACTTGTCTAACCATT
GAGGGCCTAATAGGGGAAAGATCAGAATAATATTCTTGAATATAATTATAAAAGTTTATAGGGGCATTTACAATTGTCCAGGTAGTTTCCTCCAAAAATCTA
GCCAAGGAGTCTCTAAAAAATCTTTCTGTTCTTCTTTGCAATTCTTGTGAGGTTATAGCAGGTATATCATCCCTAATAACATGCCACAAAGCCTGGGAAATA
GTAGCAAACAAGGAAGGACCCCAATGCCTAGGATCAAGGTATTGAATATTATTTACAAAAGTATTTACACCAGGAAACAAAATATCATAGTACTCATCTGG
GTTAAACAATTCCAAAGCCATGCCTGATTGCTGATAGAGGCCTACAGTGGAAACTTTGTGATCCCAATCACTAAAAAACCTATACCCTACTTGAGCCAAGGA
ACTAATACCAGTAACAGTTTGAATTAAAGCAGCAAACCCAGCAATAGCCCCAGGAGCACCAGCAATTACAGCATATGTTTGAGGAGTTAGGCCTATAGCAG
CTATAGCCTCTGAGGTACTTGTTATGCCCTCTACAGTAGCAAGGGATGCAATTTGAACTTCTATAGCAGCAGCAGCCTCCCCAGCAGCAATTTCAGCCACTG
AAAATCCTGTGGCAGCAGCAGCCTCAGATACACTGGCAACTAGGTCCCCCAAAAGTGCTAGAGCAGCACCCATCAACCTGGAAATACAAAAAAAAAGGGA
TTACTTACCTAGGAGTCTTTTACAGAGTCTTTTACAGCAGGTAAAGCAGTGGTACTTTTGTTTTTCCCGTCTACACTGTCTTCACCTCTACAAAATTCCAGCAA
AAGCTCTAAAATAAAAATAAAAATCCTCTGAGCTCTTTTTTTTGTTCCAGTCCAGGTTTTACTAACTTTCACAGAAGCTTGTCGTGACAGCTGGCGCAGAACC
ATGGCCTTTGTCCAGTTTAACTGGGGACAAGGCCAAGATTCCTAGGCTCGCAAAACATGTCTGTCTGGCTGCTTTCCACTCCTTTGGCCAGTTTCCACTTCCT
CTTGTGTTTATTTGAGAATTTTAGGGGCGGGGTTTCACTATTAACTGCCACTGGCTGGCTGCCCAGTCATGCACTTTCCTTCCTGAGGTCATGGTTTGGCTAC
ATTCCATGGGTTAGCAGCTCCTCCCTGTGGCCTTTTTTTTTATAATATATAAGAGGCCGAGGCCGCCTCGGCCTCCACCCTTTCTCAGAAGTAGTAAGGGTG
TGGAGGCTTTTTCTGAGGCCTAGCAAAAATATTTGGGGAAATCCCTATTCTTTTGCAATTTTTGCAAAAATGGATAAAGTGCTTAACAGGGAAGAATCCATG
GAGCTCATGGACCTTTTAGGCCTTGAAAGAGCTGCCTGGGGAAACCTTCCCTTAATGAGAAAAGCCTATTTAAGGAAGTGTAAGGAATTTCACCCTGACAA
AGGGGGCGACGAGGATAAAATGAAGAGAATGAATACTTTATATAAAAAAATGGAGCAGGATGTAAAGGTAGCTCATCAGCCTGATTTTGGAACCTGGAG
TAGCTCAGAGGTTTGTGCTGATTTTCCTCTTTGCCCAGATACCCTGTACTGCAAGGATTGGCCTATTTGTTCCAAAAAGCCTTCTGTGCACTGCCCTTGCATG
CTATGTCAGCTTAGATTAAGGCATTTAAATAGAAAATTTTTAAGAAAAGAGCCCTTGGTTTGGATAGATTGCTACTGCATTGACTGCTTCACACAGTGGTTT
GGCTTAGACCTAACTGAAGAAACTCTGCAATGGTGGGTCCAAATAATTGGAGAAACTCCCTTCAGAGATCTAAAGCTTTAAGGTAACTAACTTATATTTAGA
TAAATAAGAAAATATTTAAAGGCCCTAAGTAATTATTTTTTTTATAGGTGCCAACCTATGGAACAGAAGAGTGGGAGTCCTGGTGGAGCTCCTTTAATGAA
AAATGGGATGAAGATTTATTTTGCCATGAAGATATGTTTGCCAGTGATGAAGAAGCAACAGCAGATTCTCAACACTCCACACCACCCAAGAAAAAGAGAA
AGGTAGAAGACCCTAAAGACTTTCCTTCTGATCTCTGTCTGGCTGCTTTCCACTGACCTTTGGCCAGTTTCCACGTTCCTCTTGTAGTTTATTCTGATGAATTT
TAGGGGCGGGGTTTCACTATTAACTGCCACTGGCTGGCTGCCCAGTCATGCACTTTCCTTCCTGAGGTCATGGTTTGGCTACATTCCATGGGTTAGCAGCTC
CTCCCTGTGGCCTTTTTTTTTATAATATATAAGAGGCCGAGGCCGCCTTCGGCCTCCACCCTTTCTCAGAAGTAGTAAGGGTGTGGAGGCTTTTTCTGAGGC
CTAGCAAAAATATTTGGGGAAATCCCTATTCTTTTGCAATTTTTGCAAAAATGGATAAAGTGCTTAACAGGGAAGAATCCATGGAGCTCATGGACCTTTTAG
GCCTTGAAAGAGCTGCCTGGGGAAACCTTCCCTTAATGAGAAAAGCCTATTTAAGGAAGTGTAAGGAATTTCACCCTGACAAAGGGGGCGACGAGGATAA
AATGAAGAGAATGAATACTTTATATAAAAAAATGAAGCAGGATGTAAAGGTAGCTCATCAGCCTGATTTTGGAACCTGGAGTAGCTCAGAGGTTTGTGCT
GATTTTCCTCTTTGCCCAGATACCCTGTACTGCAAGGATTGGCCTATTTGTTCCAAAAAGCCTTCTGTGCACTGCCCTTGCATGCTATGTCAGCTTAGATTAA
GGCATTTAAATAGAAAATTTTTAAGAAAAGAGCCCTTGGTTTGGATAGATTGCTACTGCATTGACTGCTTCACACAGTGGTTTGGCTTAGACCTAACTGAAG
AAACTCTGCAATGGTGGGTCCAAATAATTGGAGAAACTCCCTTCAGAGATCTAAAGCTTTAAGGTAACTAACTTATATTTAGATAAATAAGAAAATATTTAA
AGGCCCTAAGTAATTATTTTTAAATATATTTAAGAAAATCCTTACAAAACTCAGAGTTCTTACTTGAAAAAAGAATTTTACAAAGTGGAATGACCTTGTTGCT
ACTGCTAATTTGGTTTAGGCCTGTAGCTGATTTTGCAACAGATATACAATCTAGAATTGTTGAATGGAAGGAAAGGCTGGATTCTGAGATAAGTATGTATA
CTTTTTCAAGGATGAAATATAATATATGCATGGGGAAATGTATTCTTGATATCACAAGAGAAGAAGATTCAGAAACTGAAGACTCTGGACATGGATCAAGC
ACTGAATCCCAATCACAATGCTCTTCCCAAGTCTGAGATACTTCAGCCCCTGCTGAAGATTCCCAAAGGTCAGACCCCCATAGTCAAGAGTTGCATTTGTGT
AAAGGCTTTCAATGTTTTAAAAGGCCTAAAACACCACCCCCAAAATAACACAAGCTTAAAAGTGGCTTATACAAAAGCAGCATTTATTAAATGTATATGTAC
AATAAAAGCACCTGTTTAAAGCATTTTGGTTTGCAATTGTCCTTGTTTATCAATATATCTTATCATATCTGGGTCCCCTGGAAGTCTTTCTGTGCCATCAAACA
CCCTAACCTCTTCTACCTGGGATTCCATACCATACATAGGCTGCCCATCCACTCTCTGGGTTCTCCTGTTTATAAGGTCACTCAGCAAAAAGGAAATTGGGTA
AGGATTTTTTACAGATCTTTTTCTCAGGCGAATCTTAAAATATCTTGCAAGGCCTCTCCACTGTTGTGTTCCAGAGCTGTTAGTAAACAGGCCACAAATATCA
GCAGCTGAAACATACAGGCTATCAGCTTTACAAAGAGGCCCCACACCCTGTTCATCTAGCAACACTGTGGTAGCTGTGTTGGTCACATGAAGTACTGGGGG
AACATTTTCCCCTCCTGTGAAAGTCCCAAAATACCTAGTATTTTCATTTCTACTGGGATCAGGAACCCAGCACTCAACTGGATAAGCATTGTTTTTGTCCAAA
TAGGCCTTATGGTCAGTATTCATTACCTGGGACTGGGCTGTTGGGTTTTTTGGGGTTATAGTACCATCTGGGTACTTGGTCCTGTAATTCATTAGCACTCCCT
GCATTTCCAAGGGGTCTCCACCAACAGCAAAGAAGTGGAAATTACTGCCTTGAATAGGTTTACCTCCACCATGCTCATGCACTTTTTGTGACCCTGCATGAA
GGTTAAGCATGCTAGTTATTCCAATGACCTCTGTTTGTACAGTTACAGCCTCCCACATCAGTAGATTTCCACAGGTTAGGTCCTCATTTAAATTGGGGAGGG
GAATTCTTGCTGTGCTGTAACAGGGAAGCATTTTTCTTTCTGGGCTATCACTGCTAAAGTCATTTTCAGCACTTAGCTTTAGACTAAAGCCCCTAAGGTTTTC
ATCTGGATCCCCCATTTCTGGGTTTAGGAAGCATTCTACCTCTGTTATAGCATCTACCCCAGTTTTAACTTCTAGAACTTCTACTCCTCCTTTTATTAGTAGTTT
TGGCACTTGCACGGGTTCCTTTGGCTTTTTGGGAGCTGCCCCTGGACACTCTCCTTTTCTTTTGGTTGGGGCCATCTTCATATGCTTCAAGAGCAGGTGTTAC
AGTCCCGTACAGGCCTAGAAGTAAAGGCAACATCCATTGAGGAGCAGTTCTTTGATTAGCACCTCCTGGGGCAATAGTTTTTTCTATAAACTCTCCTGAATG
TACAGTTTGTTGATTTCTTAAGTCCATTCTTTGTGTAACTTCTTCTATACTGTCAGCATCATCTATACTATAAGTATGGCCAAAATGTACACGGGTACCTTCCC
TTTCAGCCACTTGTCTAACCATTGAGGGCCTAATAGGGGAAAGATCAGAATAATATTCTTGAATATAATTATAAAAGTTTATAGGGGCATTTACAATTGTCC
AGGTAGTTTCCTCCAAAAATCTAGCCAAGGAGTCTCTAAAAAATCTTTCTGTTCTTCTTTGCAATTCTTGTGAGGTTATAGCAGGTATATCATCCCTAATAAC
ATGCCACAAAGCCTGGGAAATAGTAGCAAACAAGGAAGGACCCCAATGCCTAGGATCAAGGTATTGAATATTATTTACAAAAGTATTTACACCAGGAAAC
AAAATATCATAGTACTCATCTGGGTTAAACAATTCCAAAGCCATGCCTGATTGCTGATAGAGGCCTACAGTGGAAACTTTGTGATCCCAATCACTAAAAAAC
CTATACCCTACTTGAGCCAAGGAACTAATACCAGTAACAGTTTGAATTAAAGCAGCAAACCCAGCAATAGCCCCAGGAGCACCAGCAATTACAGCATATGT
TTGAGGAGTTAGGCCTATAGCAGCTATAGCCTCTGAGGTACTTGTTATGCCCTCTACAGTAGCAAGGGATGCAATTTGAACTTCTATAGCAGCAGCAGCCT
CCCCAGCAGCAATTTCAGCCACTGAAAATCCTGTGGCAGCAGCAGCCTCAGATACACTGGCAACTAGGTCCCCCAAAAGTGCTAGAGCAGCACCCATCAAC

CTGGAAATACAAAAAAAAAGGGATTACTTACCTAGGAGTCTTTTACAGAGTCTTTTACAGCAGGTAAAGCAGTGGTACTTTTGTTTTTCCCGTCTACACTGT
CTTCACCTCTACAAAATTCCAGCAAAAGCTCTAAAATAAAAATAAAAATCCTCTGAGCTCTTTTTTTTGTTCCAGTCCAGGTTTTACTAACTTTCACAGAAGCT
TGTCGTGACAGCTGGCGCAGAACCATGGCCTTTGTCCAGTTTAACTGGGGACAAGGCCAAGATTCCTAGGCTCGCAAAACATGTCTGTCTGGCTGCTTTCC
ACTCCTTTGGCCAGTTTCCACTTCCTCTTGTGTTTATTTGAGAATTTTAGGGGCGGGGTTTCACTATTAACTGCCACTGGCTGGCTGCCCAGTCATGCACTTTC
CTTCCTGAGGTCATGGTTTGGCTACATTCCATGGGTTAGCAGCTCCTCCCTGTGGCCTTTTTTTTTATAATATATAAGAGGCCGAGGCCGCCTCGGCCTCCAC
CCTTTCTCAGAAGTAGTAAGGGTGTGGAGGCTTTTTCTGAGGCCTAGCAAAAATATTTGGGGAAATCCCTATTCTTTTGCAATTTTTGCAAAAATGGATAAA
GTGCTTAACAGGGAAGAATCCATGGAGCTCATGGACCTTTTAGGCCTTGAAAGAGCTGCCTGGGGAAACCTTCCCTTAATGAGAAAAGCCTATTTAAGGAA
GTGTAAGGAATTTCACCCTGACAAAGGGGGCGACGAGGATAAAATGAAGAGAATGAATACTTTATATAAAAAAATGGAGCAGGATGTAAAGGTAGCTCA
TCAGCCTGATTTTGGAACCTGGAGTAGCTCAGAGGTTTGTGCTGATTTTCCTCTTTGCCCAGATACCCTGTACTGCAAGGATTGGCCTATTTGTTCCAAAAA
GCCTTCTGTGCACTGCCCTTGCATGCTATGTCAGCTTAGATTAAGGCATTTAAATAGAAAATTTTTAAGAAAAGAGCCCTTGGTTTGGATAGATTGCTACTG
CATTGACTGCTTCACACAGTGGTTTGGCTTAGACCTAACTGAAGAAACTCTGCAATGGTGGGTCCAAATAATTGGAGAAACTCCCTTCAGAGATCTAAAGC
TTTAAGGTAACTAACTTATATTTAGATAAATAAGAAAATATTTAAAGGCCCTAAGTAATTATTTTTTTTATAGGTGCCAACCTATGGAACAGAAGAGTGGGA
GTCCTGGTGGAGCTCCTTTAATGAAAAATGGGATGAAGATTTATTTTGCCATGAAGATATGTTTGCCAGTGATGAAGAAGCAACAGCAGATTCTCAACACT
CCACACCACCCAAGAAAAAGAGAAAGGTAGAAGACCCTAAAGACTTTCCTTCTGATCTCTGTCTGGCTGCTTTCCACTCCTTTGGCCAGTTTCCACTTCCTCT
TGTGTTTATTTGAGAATTTTAGGGGCGGGGTTTCACTATTAACTGCCACTGGCTGGCTGCCCAGTCATGCACTTTCCTTCCTGAGGTCATGGTTTGGCTACAT
TCCATGGGTTAGCAGCTCCTCCCTGTGGCCTTTTTTTTTATAATATATAAGAGGCCGAGGCCGCCTCGGCCTCCACCCTTTCTCAGAAGTAGTAAGGGTGTG
GAGGCTTTTTCTGAGGCCTAGCAAAAATATTTGGGGAAATCCCTATTCTTTTGCAATTTTTGCAAAAATGGATAAAGTGCTTAACAGGGAAGAATCCATGG
AGCTCATGGACCTTTTAGGCCTTGAAAGAGCTGCCTGGGGAAACCTTCCCTTAATGAGAAAAGCCTATTTAAGGAAGTGTAAGGAATTTCACCCTGACAAA
GGGGGCGACGAGGATAAAATGAAGAGAATGAATACTTTATATAAAAAAATGAAGCAGGATGTAAAGGTAGCTCATCAGCCTGATTTTGGAACCTGGAGT
AGCTCAGAGGTTTGTGCTGATTTTCCTCTTTGCCCAGATACCCTGTACTGCAAGGATTGGCCTATTTGTTCCAAAAAGCCTTCTGTGCACTGCCCTTGCATGC
TATGTCAGCTTAGATTAAGGCATTTAAATAGAAAATTTTTAAGAAAAGAGCCCTTGGTTTGGATAGATTGCTACTGCATTGACTGCTTCACACAGTGGTTTG
GCTTAGACCTAACTGAAGAAACTCTGCAATGGTGGGTCCAAATAATTGGAGAAACTCCCTTCAGAGATCTAAAGCTTTAAGGTAACTAACTTATATTTAGAT
AAATAAGAAAATATTTAAAGGCCCTAAGTAATTATTTTTAAATATATTTAAGAAAATCCTTACAAAACTCAGAGTTCTTACTTGAAAAAAGAATTTTACAAAG
TGGAATGACCTTGTTGCTACTGCTAATTTGGTTTAGGCCTGTAGCTGATTTTGCAACAGATATACAATCTAGAATTGTTGAATGGAAGGAAAGGCTGGATTC
TGAGATAAGTATGTATACTTTTTCAAGGATGAAATATAATATATGCATGGGGAAATGTATTCTTGATATCACAAGAGAAGAAGATTCAGAAACTGAAGACT
CTGGACATGGATCAAGCACTGAATCCCAATCACAATGCTCTTCCCAAGTCTGAGATACTTCAGCCCCTGCTGAAGATTCCCAAAGGTCAGACCCCCATAGTC
AAGAGTTGCATTTGTGTAAAGGCTTTCAATGTTTTAAAAGGCCTAAAACACCACCCCCAAAATAACACAAGCTTAAAAGTGGCTTATACAAAAGCAGCATTT
ATTAAATGTATATGTACAATAAAAGCACCTGTTTAAAGCATTTTGGTTTGCAATTGTCCTTGTTTATCAATATATCTTATCATATCTGGGTCCCCTGGAAGTCT
TTCTGTGCCATCAAACACCCTAACCTCTTCTACCTGGGATTCCATACCATACATAGGCTGCCCATCCACTCTCTGGGTTCTCCTGTTTATAAGGTCACTCAGCA
AAAAGGAAATTGGGTAAGGATTTTTTACAGATCTTTTTCTCAGGCGAATCTTAAAATATCTTGCAAGGCCTCTCCACTGTTGTGTTCCAGAGCTGTTAGTAA
ACAGGCCACAAATATCAGCAGCTGAAACATACAGGCTATCAGCTTTACAAAGAGGCCCCACACCCTGTTCATCTAGCAACACTGTGGTAGCTGTGTTGGTC
ACATGAAGTACTGGGGGAACATTTTCCCCTCCTGTGAAAGTCCCAAAATACCTAGTATTTTCATTTCTACTGGGATCAGGAACCCAGCACTCAACTGGATAA
GCATTGTTTTTGTCCAAATAGGCCTTATGGTCAGTATTCATTACCTGGGACTGGGCTGTTGGGTTTTTTGGGGTTATAGTACCATCTGGGTACTTGGTCCTGT
AATTCATTAGCACTCCCTGCATTTCCAAGGGGTCTCCACCAACAGCAAAGAAGTGGAAATTACTGCCTTGAATAGGTTTACCTCCACCATGCTCATGCACTTT
TTGTGACCCTGCATGAAGGTTAAGCATGCTAGTTATTCCAATGACCTCTGTTTGTACAGTTACAGCCTCCCACATCAGTAGATTTCCACAGGTTAGGTCCTCA
TTTAAATTGGGGAGGGGAATTCTTGCTGTGCTGTAACAGGGAAGCATTTTTCTTTCTGGGCTATCACTGCTAAAGTCATTTTCAGCACTTAGCTTTAGACTA
AAGCCCCTAAGGTTTTCATCTGGATCCCCCATTTCTGGGTTTAGGAAGCATTCTACCTCTGTTATAGCATCTACCCCAGTTTTAACTTCTAGAACTTCTACTCC
TCCTTTTATTAGTAGTTTTGGCACTTGCACGGGTTCCTTTGGCTTTTTGGGAGCTGCCCCTGGACACTCTCCTTTTCTTTTGGTTGGGGCCATCTTCATATGCT
TCAAGAGCAGGTGTTACAGTCCCGTACAGGCCTAGAAGTAAAGGCAACATCCATTGAGGAGCAGTTCTTTGATTAGCACCTCCTGGGGCAATAGTTTTTTC
TATAAACTCTCCTGAATGTACAGTTTGTTGATTTCTTAAGTCCATTCTTTGTGTAACTTCTTCTATACTGTCAGCATCATCTATACTATAAGTATGGCCAAAAT
GTACACGGGTACCTTCCCTTTCAGCCACTTGTCTAACCATTGAGGGCCTAATAGGGGAAAGATCAGAATAATATTCTTGAATATAATTATAAAAGTTTATAG
GGGCATTTACAATTGTCCAGGTAGTTTCCTCCAAAAATCTAGCCAAGGAGTCTCTAAAAAAATCTTTCTGTTCTTCTTTGCAATTCTTGTGAGGTTATAGCAGG
TATATCATCCCTAATAACATGCCACAAAGCCTGGGAAATAGTAGCAAACAAGGAAGGACCCCAATGCCTAGGATCAAGGTATTGAATATTATTTACAAAAG
TATTTACACCAGGAAACAAAATATCATAGTACTCATCTGGGTTAAACAATTCCAAAGCCATGCCTGATTGCTGATAGAGGCCTACAGTGGAAACTTTGTGAT
CCCAATCACTAAAAAACCTATACCCTACTTGAGCCAAGGAACTAATACCAGTAACAGTTTGAATTAAAGCAGCAAACCCAGCAATAGCCCCAGGAGCACCA
GCAATTACAGCATATGTTTGAGGAGTTAGGCCTATAGCAGCTATAGCCTCTGAGGTACTTGTTATGCCCTCTACAGTAGCAAGGGATGCAATTTGAACTTCT
ATAGCAGCAGCAGCCTCCCCAGCAGCAATTTCAGCCACTGAAAATCCTGTGGCAGCAGCAGCCTCAGATACACTGGCAACTAGGTCCCCCAAAAGTGCTAG
AGCAGCACCCATCAACCTGGAAATACAAAAAAAAAGGGATTACTTACCTAGGAGTCTTTTACAGAGTCTTTTACAGCAGGTAAAGCAGTGGTACTTTTGTTT
TTCCCGTCTACACTGTCTTCACCTCTACAAAATTCCAGCAAAAGCTCTAAAATAAAAATAAAAATCCTCTGAGCTCTTTTTTTTGTTCCAGTCCAGGTTTTACT
AACTTTCACAGAAGCTTGTCGTGACAGCTGGCGCAGAACCATGGCCTTTGTCCAGTTTAACTGGGGACAAGGCCAAGATTCCTAGGCTCGCAAAACATGTC
TGTCTGGCTGCTTTCCACTCCTTTGGCCAGTTTCCACTTCCTCTTGTGTTTATTTGAGAATTTTAGGGGCGGGGTTTCACTATTAACTGCCACTGGCTGGCTGC
CCAGTCATGCACTTTCCTTCCTGAGGTCATGGTTTGGCTACATTCCATGGGTTAGCAGCTCCTCCCTGTGGCCTTTTTTTTTATAATATATAAGAGGCCGAGG
CCGCCTCGGCCTCCACCCTTTCTCAGAAGTAGTAAGGGTGTGGAGGCTTTTTCTGAGGCCTAGCAAAAATATTTGGGGAAATCCCTATTCTTTTGCAATTTTT
GCAAAAATGGATAAAGTGCTTAACAGGGAAGAATCCATGGAGCTCATGGACCTTTTAGGCCTTGAAAGAGCTGCCTGGGGAAACCTTCCCTTAATGAGAA
AAGCCTATTTAAGGAAGTGTAAGGAATTTCACCCTGACAAAGGGGGCGACGAGGATAAAATGAAGAGAATGAATACTTTATATAAAAAAATGGAGCAGG
ATGTAAAGGTAGCTCATCAGCCTGATTTTGGAACCTGGAGTAGCTCAGAGGTTTGTGCTGATTTTCCTCTTTGCCCAGATACCCTGTACTGCAAGGATTGGC
CTATTTGTTCCAAAAAGCCTTCTGTGCACTGCCCTTGCATGCTATGTCAGCTTAGATTAAGGCATTTAAATAGAAAATTTTTAAGAAAAGAGCCCTTGGTTTG
GATAGATTGCTACTGCATTGACTGCTTCACACAGTGGTTTGGCTTAGACCTAACTGAAGAAACTCTGCAATGGTGGGTCCAAATAATTGGAGAAACTCCCTT
CAGAGATCTAAAGCTTTAAGGTAACTAACTTATATTTAGATAAATAAGAAAATATTTAAAGGCCCTAAGTAATTATTTTTTTTATAGGTGCCAACCTATGGAA
CAGAAGAGTGGGAGTCCTGGTGGAGCTCCTTTAATGAAAAATGGGATGAAGATTTATTTTGCCATGAAGATATGTTTGCCAGTGATGAAGAAGCAACAGC
AGATTCTCAACACTCCACACCACCCAAGAAAAAGAGAAAGGTAGAAGACCCTAAAGACTTTCCTTCTGATCTCTGTCTGGCTGCTTTCCACTCCTTTGGCCA
GTTTCCACTTCCTCTTGTGTTTATTTGAGAATTTTAGGGGCGGGGTTTCACTATTAACTGCCACTGGCTGGCTGCCCAGTCATGCACTTTCCTTCCTGAGGTC
ATGGTTTGGCTACATTCCATGGGTTAGCAGCTCCTCCCTGTGGCCTTTTTTTTTATAATATATAAGAGGCCGAGGCCGCCTCGGCCTCCACCCTTTCTCAGAA
GTAGTAAGGGTGTGGAGGCTTTTTCTGAGGCCTAGCAAAAATATTTGGGGAAATCCCTATTCTTTTGCAATTTTTGCAAAAATGGATAAAGTGCTTAACAG
GGAAGAATCCATGGAGCTCATGGACCTTTTAGGCCTTGAAAGAGCTGCCTGGGGAAACCTTCCCTTAATGAGAAAAGCCTATTTAAGGAAGTGTAAGGAA
TTTCACCCTGACAAAGGGGGCGACGAGGATAAAATGAAGAGAATGAATACTTTATATAAAAAAATGAAGCAGGATGTAAAGGTAGCTCATCAGCCTGATT
TTGGAACCTGGAGTAGCTCAGAGGTTTGTGCTGATTTTCCTCTTTGCCCAGATACCCTGTACTGCAAGGATTGGCCTATTTGTTCCAAAAAGCCTTCTGTGCA

CTGCCCTTGCATGCTATGTCAGCTTAGATTAAGGCATTTAAATAGAAAATTTTTAAGAAAAGAGCCCTTGGTTTGGATAGATTGCTACTGCATTGACTGCTTC
ACACAGTGGTTTGGCTTAGACCTAACTGAAGAAACTCTGCAATGGTGGGTCCAAATAATTGGAGAAACTCCCTTCAGAGATCTAAAGCTTTAAGGTAACTA
ACTTATATTTAGATAAATAAGAAAATATTTAAAGGCCCTAAGTAATTATTTTTAAATATATTTAAGAAAATCCTTACAAAACTCAGAGTTCTTACTTGAAAAA
AGAATTTTACAAAGTGGAATGACCTTGTTGCTACTGCTAATTTGGTTTAGGCCTGTAGCTGATTTTGCAACAGATATACAATCTAGAATTGTTGAATGGAAG
GAAAGGCTGGATTCTGAGATAAGTATGTATACTTTTTCAAGGATGAAATATAATATATGCATGGGGAAATGTATTCTTGATATCACAAGAGAAGAAGATTC
AGAAACTGAAGACTCTGGACATGGATCAAGCACTGAATCCCAATCACAATGCTCTTCCCAAGTCTGAGATACTTCAGCCCCTGCTGAAGATTCCCAAAGGTC
AGACCCCCATAGTCAAGAGTTGCATTTGTGTAAAGGCTTTCAATGTTTTAAAAGGCCTAAAACACCACCCCCAAAATAACACAAGCTTAAAAGTGGCTTATA
CAAAAGCAGCATTTATTAAATGTATATGTACAATAAAAGCACCTGTTTAAAGCATTTTGGTTTGCAATTGTCCTTGTTTATCAATATATCTTATCATATCTGGG
TCCCCTGGAAGTCTTTCTGTGCCATCAAACACCCTAACCTCTTCTACCTGGGATTCCATACCATACATAGGCTGCCCATCCACTCTCTGGGTTCTCCTGTTTAT
AAGGTCACTCAGCAAAAAGGAAATTGGGTAAGGATTTTTTACAGATCTTTTTCTCAGGCGAATCTTAAAATATCTTGCAAGGCCTCTCCACTGTTGTGTTCC
AGAGCTGTTAGTAAACAGGCCACAAATATCAGCAGCTGAAACATACAGGCTATCAGCTTTACAAAGAGGCCCCACACCCTGTTCATCTAGCAACACTGTGG
TAGCTGTGTTGGTCACATGAAGTACTGGGGGAACATTTTCCCCTCCTGTGAAAGTCCCAAAATACCTAGTATTTTCATTTCTACTGGGATCAGGAACCCAGC
ACTCAACTGGATAAGCATTGTTTTTGTCCAAATAGGCCTTATGGTCAGTATTCATTACCTGGGACTGGGCTGTTGGGTTTTTTGGGGTTATAGTACCATCTG
GGTACTTGGTCCTGTAATTCATTAGCACTCCCTGCATTTCCAAGGGGTCTCCACCAACAGCAAAGAAGTGGAAATTACTGCCTTGAATAGGTTTACCTCCAC
CATGCTCATGCACTTTTTGTGACCCTGCATGAAGGTTAAGCATGCTAGTTATTCCAATGACCTCTGTTTGTACAGTTACAGCCTCCCACATCAGTAGATTTCC
ACAGGTTAGGTCCTCATTTAAATTGGGGAGGGGAATTCTTGCTGTGCTGTAACAGGGAAGCATTTTTCTTTCTGGGCTATCACTGCTAAAGTCATTTTCAGC
ACTTAGCTTTAGACTAAAGCCCCTAAGGTTTTCATCTGGATCCCCCATTTCTGGGTTTAGGAAGCATTCTACCTCTGTTATAGCATCTACCCCAGTTTTAACTT
CTAGAACTTCTACTCCTCCTTTTATTAGTAGTTTTGGCACTTGCACGGGTTCCTTTGGCTTTTTGGGAGCTGCCCCTGGACACTCTCCTTTTCTTTTGGTTGGG
GCCATCTTCATATGCTTCAAGAGCAGGTGTTACAGTCCCGTACAGGCCTAGAAGTAAAGGCAACATCCATTGAGGAGCAGTTCTTTGATTAGCACCTCCTG
GGGCAATAGTTTTTTCTATAAACTCTCCTGAATGTACAGTTTGTTGATTTCTTAAGTCCATTCTTTGTGTAACTTCTTCTATACTGTCAGCATCATCTATACTAT
AAGTATGGCCAAAATGTACACGGGTACCTTCCCTTTCAGCCACTTGTCTAACCATTGAGGGCCTAATAGGGGAAAGATCAGAATAATATTCTTGAATATAAT
TATAAAAGTTTATAGGGGCATTTACAATTGTCCAGGTAGTTTCCTCCAAAAATCTAGCCAAGGAGTCTCTAAAAAATCTTTCTGTTCTTCTTTGCAATTCTTGT
GAGGTTATAGCAGGTATATCATCCCTAATAACATGCCACAAAGCCTGGGAAATAGTAGCAAACAAGGAAGGACCCCAATGCCTAGGATCAAGGTATTGAA
TATTATTTACAAAAGTATTTACACCAGGAAACAAAATATCATAGTACTCATCTGGGTTAAACAATTCCAAAGCCATGCCTGATTGCTGATAGAGGCCTACAG
TGGAAACTTTGTGATCCCAATCACTAAAAAACCTATACCCTACTTGAGCCAAGGAACTAATACCAGTAACAGTTTGAATTAAAGCAGCAAACCCAGCAATA
GCCCCAGGAGCACCAGCAATTACAGCATATGTTTGAGGAGTTAGGCCTATAGCAGCTATAGCCTCTGAGGTACTTGTTATGCCCTCTACAGTAGCAAGGGA
TGCAATTTGAACTTCTATAGCAGCAGCAGCCTCCCCAGCAGCAATTTCAGCCACTGAAAATCCTGTGGCAGCAGCAGCCTCAGATACACTGGCAACTAGGT
CCCCCAAAAGTGCTAGAGCAGCACCCATCAACCTGGAAATACAAAAAAAAAGGGATTACTTACCTAGGAGTCTTTTACAGAGTCTTTTACAGCAGGTAAAG
CAGTGGTACTTTTGTTTTTCCCGTCTACACTGTCTTCACCTCTACAAAATTCCAGCAAAAGCTCTAAAATAAAAATAAAAATCCTCTGAGCTCTTTTTTTTGTTC
CAGTCCAGGTTTTACTAACTTTCACAGAAGCTTGTCGTGACAGCTGGCGCAGAACCATGGCCTTTGTCCAGTTTAACTGGGGACAAGGCCAAGATTCCTAG
GCTCGCAAAACATGTCTGTCTGGCTGCTTTCCACTCCTTTGGCCAGTTTCCACTTCCTCTTGTGTTTATTTGAGAATTTTAGGGGCGGGGTTTCACTATTAACT
GCCACTGGCTGGCTGCCCAGTCATGCACTTTCCTTCCTGAGGTCATGGTTTGGCTACATTCCATGGGTTAGCAGCTCCTCCCTGTGGCCTTTTTTTTTATAATA
TATAAGAGGCCGAGGCCGCCTCGGCCTCCACCCTTTCTCAGAAGTAGTAAGGGTGTGGAGGCTTTTTCTGAGGCCTAGCAAAAATATTTGGGGAAATCCCT
ATTCTTTTGCAATTTTTGCAAAAATGGATAAAGTGCTTAACAGGGAAGAATCCATGGAGCTCATGGACCTTTTAGGCCTTGAAAGAGCTGCCTGGGGAAAC
CTTCCCTTAATGAGAAAAGCCTATTTAAGGAAGTGTAAGGAATTTCACCCTGACAAAGGGGGCGACGAGGATAAAATGAAGAGAATGAATACTTTATATA
AAAAAATGGAGCAGGATGTAAAGGTAGCTCATCAGCCTGATTTTGGAACCTGGAGTAGCTCAGAGGTTTGTGCTGATTTTCCTCTTTGCCCAGATACCCTG
TACTGCAAGGATTGGCCTATTTGTTCCAAAAAGCCTTCTGTGCACTGCCCTTGCATGCTATGTCAGCTTAGATTAAGGCATTTAAATAGAAAATTTTTAAGAA
AAGAGCCCTTGGTTTGGATAGATTGCTACTGCATTGACTGCTTCACACAGTGGTTTGGCTTAGACCTAACTGAAGAAACTCTGCAATGGTGGGTCCAAATA
ATTGGAGAAACTCCCTTCAGAGATCTAAAGCTTTAAGGTAACTAACTTATATTTAGATAAATAAGAAAATATTTAAAGGCCCTAAGTAATTATTTTTTTTATA
GGTGCCAACCTATGGAACAGAAGAGTGGGAGTCCTGGTGGAGCTCCTTTAATGAAAAATGGGATGAAGATTTATTTTGCCATGAAGATATGTTTGCCAGT
GATGAAGAAGCAACAGCAGATTCTCAACACTCCACACCACCCAAGAAAAAGAGAAAGGTAGAAGACCCTAAAGACTTTCCTTCTGATCTCTGTCTGGCTGC
TTTCCACTCCTTTGGCCAGTTTCCACTTCCTCTTGTGTTTATTTGAGAATTTTAGGGGCGGGGTTTCACTATTAACTGCCACTGGCTGGCTGCCCAGTCATGCA
CTTTCCTTCCTGAGGTCATGGTTTGGCTACATTCCATGGGTTAGCAGCTCCTCCCTGTGGCCTTTTTTTTTATAATATATAAGAGGCCGAGGCCGCCTCGGCC
TCCACCCTTTCTCAGAAGTAGTAAGGGTGTGGAGGCTTTTTCTGAGGCCTAGCAAAAATATTTGGGGAAATCCCTATTCTTTTGCAATTTTTGCAAAAATGG
ATAAAGTGCTTAACAGGGAAGAATCCATGGAGCTCATGGACCTTTTAGGCCTTGAAAGAGCTGCCTGGGGAAACCTTCCCTTAATGAGAAAAGCCTATTTA
AGGAAGTGTAAGGAATTTCACCCTGACAAAGGGGGCGACGAGGATAAAATGAAGAGAATGAATACTTTATATAAAAAAATGAAGCAGGATGTAAAGGTA
GCTCATCAGCCTGATTTTGGAACCTGGAGTAGCTCAGAGGTTTGTGCTGATTTTCCTCTTTGCCCAGATACCCTGTACTGCAAGGATTGGCCTATTTGTTCCA
AAAAGCCTTCTGTGCACTGCCCTTGCATGCTATGTCAGCTTAGATTAAGGCATTTAAATAGAAAATTTTTAAGAAAAGAGCCCTTGGTTTGGATAGATTGCT
ACTGCATTGACTGCTTCACACAGTGGTTTGGCTTAGACCTAACTGAAGAAACTCTGCAATGGTGGGTCCAAATAATTGGAGAAACTCCCTTCAGAGATCTA
AAGCTTTAAGGTAACTAACTTATATTTAGATAAATAAGAAAATATTTAAAGGCCCTAAGTAATTATTTTTAAATATATTTAAGAAAATCCTTACAAAACTCAG
AGTTCTTACTTGAAAAAAGAATTTTACAAAGTGGAATGACCTTGTTGCTACTGCTAATTTGGTTTAGGCCTGTAGCTGATTTTGCAACAGATATACAATCTAG
AATTGTTGAATGGAAGGAAAGGCTGGATTCTGAGATAAGTATGTATACTTTTTCAAGGATGAAATATAATATATGCATGGGGAAATGTATTCTTGATATCA
CAAGAGAAGAAGATTCAGAAACTGAAGACTCTGGACATGGATCAAGCACTGAATCCCAATCACAATGCTCTTCCCAAGTCTGAGATACTTCAGCCCCTGCT
GAAGATTCCCAAAGGTCAGACCCCCATAGTCAAGAGTTGCATTTGTGTAAAGGCTTTCAATGTTTTAAAAGGCCTAAAACACCACCCCCAAAATAACACAA
GCTTAAAAGTGGCTTATACAAAAGCAGCATTTATTAAATGTATATGTACAATAAAAGCACCTGTTTAAAGCATTTTGGTTTGCAATTGTCCTTGTTTATCAAT
ATATCTTATCATATCTGGGTCCCCTGGAAGTCTTTCTGTGCCATCAAACACCCTAACCTCTTCTACCTGGGATTCCATACCATACATAGGCTGCCCATCCACTC
TCTGGGTTCTCCTGTTTATAAGGTCACTCAGCAAAAAGGAAATTGGGTAAGGATTTTTTACAGATCTTTTTCTCAGGCGAATCTTAAAATATCTTGCAAGGCC
TCTCCACTGTTGTGTTCCAGAGCTGTTAGTAAACAGGCCACAAATATCAGCAGCTGAAACATACAGGCTATCAGCTTTACAAAGAGGCCCCACACCCTGTTC
ATCTAGCAACACTGTGGTAGCTGTGTTGGTCACATGAAGTACTGGGGGAACATTTTCCCCTCCTGTGAAAGTCCCAAAATACCTAGTATTTTCATTTCTACTG
GGATCAGGAACCCAGCACTCAACTGGATAAGCATTGTTTTTGTCCAAATAGGCCTTATGGTCAGTATTCATTACCTGGGACTGGGCTGTTGGGTTTTTTGGG
GTTATAGTACCATCTGGGTACTTGGTCCTGTAATTCATTAGCACTCCCTGCATTTCCAAGGGGTCTCCACCAACAGCAAAGAAGTGGAAATTACTGCCTTGA
ATAGGTTTACCTCCACCATGCTCATGCACTTTTTGTGACCCTGCATGAAGGTTAAGCATGCTAGTTATTCCAATGACCTCTGTTTGTACAGTTACAGCCTCCC
ACATCAGTAGATTTCCACAGGTTAGGTCCTCATTTAAATTGGGGAGGGGAATTCTTGCTGTGCTGTAACAGGGAAGCATTTTTCTTTCTGGGCTATCACTGC
TAAAGTCATTTTCAGCACTTAGCTTTAGACTAAAGCCCCTAAGGTTTTCATCTGGATCCCCCATTTCTGGGTTTAGGAAGCATTCTACCTCTGTTATAGCATCT
ACCCCAGTTTTAACTTCTAGAACTTCTACTCCTCCTTTTATTAGTAGTTTTGGCACTTGCACGGGTTCCTTTGGCTTTTTGGGAGCTGCCCCTGGACACTCTCC
TTTTCTTTTGGTTGGGGCCATCTTCATATGCTTCAAGAGCAGGTGTTACAGTCCCGTACAGGCCTAGAAGTAAAGGCAACATCCATTGAGGAGCAGTTCTTT
GATTAGCACCTCCTGGGGCAATAGTTTTTTCTATAAACTCTCCTGAATGTACAGTTTGTTGATTTCTTAAGTCCATTCTTTGTGTAACTTCTTCTATACTGTCAG

CATCATCTATACTATAAGTATGGCCAAAATGTACACGGGTACCTTCCCTTTCAGCCACTTGTCTAACCATTGAGGGCCTAATAGGGGAAAGATCAGAATAAT
ATTCTTGAATATAATTATAAAAGTTTATAGGGGCATTTACAATTGTCCAGGTAGTTTCCTCCAAAAATCTAGCCAAGGAGTCTCTAAAAAATCTTTCTGTTCT
TCTTTGCAATTCTTGTGAGGTTATAGCAGGTATATCATCCCTAATAACATGCCACAAAGCCTGGGAAATAGTAGCAAACAAGGAAGGACCCCAATGCCTAG
GATCAAGGTATTGAATATTATTTACAAAAGTATTTACACCAGGAAACAAAATATCATAGTACTCATCTGGGTTAAACAATTCCAAAGCCATGCCTGATTGCT
GATAGAGGCCTACAGTGGAAACTTTGTGATCCCAATCACTAAAAAACCTATACCCTACTTGAGCCAAGGAACTAATACCAGTAACAGTTTGAATTAAAGCA
GCAAACCCAGCAATAGCCCCAGGAGCACCAGCAATTACAGCATATGTTTGAGGAGTTAGGCCTATAGCAGCTATAGCCTCTGAGGTACTTGTTATGCCCTC
TACAGTAGCAAGGGATGCAATTTGAACTTCTATAGCAGCAGCAGCCTCCCCAGCAGCAATTTCAGCCACTGAAAATCCTGTGGCAGCAGCAGCCTCAGATA
CACTGGCAACTAGGTCCCCCAAAAGTGCTAGAGCAGCACCCATCAACCTGGAAATACAAAAAAAAAGGGATTACTTACCTAGGAGTCTTTTACAGAGTCTT
TTACAGCAGGTAAAGCAGTGGTACTTTTGTTTTTCCCGTCTACACTGTCTTCACCTCTACAAAATTCCAGCAAAAGCTCTAAAATAAAAATAAAAATCCTCTG
AGCTCTTTTTTTTGTTCCAGTCCAGGTTTTACTAACTTTCACAGAAGCTTGTCGTGACAGCTGGCGCAGAACCATGGCCTTTGTCCAGTTTAACTGGGGACAA
GGCCAAGATTCCTAGGCTCGCAAAACATGTCTGTCTGGCTGCTTTCCACTCCTTTGGCCAGTTTCCACTTCCTCTTGTGTTTATTTGAGAATTTTAGGGGCGG
GGTTTCACTATTAACTGCCACTGGCTGGCTGCCCAGTCATGCACTTTCCTTCCTGAGGTCATGGTTTGGCTACATTCCATGGGTTAGCAGCTCCTCCCTGTGG
CCTTTTTTTTTATAATATATAAGAGGCCGAGGCCGCCTCGGCCTCCACCCTTTCTCAGAAGTAGTAAGGGTGTGGAGGCTTTTTCTGAGGCCTAGCAAAAAT
ATTTGGGGAAATCCCTATTCTTTTGCAATTTTTGCAAAAATGGATAAAGTGCTTAACAGGGAAGAATCCATGGAGCTCATGGACCTTTTAGGCCTTGAAAGA
GCTGCCTGGGGAAACCTTCCCTTAATGAGAAAAGCCTATTTAAGGAAGTGTAAGGAATTTCACCCTGACAAAGGGGGCGACGAGGATAAAATGAAGAGA
ATGAATACTTTATATAAAAAAATGGAGCAGGATGTAAAGGTAGCTCATCAGCCTGATTTTGGAACCTGGAGTAGCTCAGAGGTTTGTGCTGATTTTCCTCTT
TGCCCAGATACCCTGTACTGCAAGGATTGGCCTATTTGTTCCAAAAAGCCTTCTGTGCACTGCCCTTGCATGCTATGTCAGCTTAGATTAAGGCATTTAAATA
GAAAATTTTTAAGAAAAGAGCCCTTGGTTTGGATAGATTGCTACTGCATTGACTGCTTCACACAGTGGTTTGGCTTAGACCTAACTGAAGAAACTCTGCAAT
GGTGGGTCCAAATAATTGGAGAAACTCCCTTCAGAGATCTAAAGCTTTAAGGTAACTAACTTATATTTAGATAAATAAGAAAATATTTAAAGGCCCTAAGTA
ATTATTTTTTTTATAGGTGCCAACCTATGGAACAGAAGAGTGGGAGTCCTGGTGGAGCTCCTTTAATGAAAAATGGGATGAAGATTTATTTTGCCATGAAG
ATATGTTTGCCAGTGATGAAGAAGCAACAGCAGATTCTCAACACTCCACACCACCCAAGAAAAAGAGAAAGGTAGAAGACCCTAAAGACTTTCCTTCTGAT
CTCTGTCTGGCTGCTTTCCACTCCTTTGGCCAGTTTCCACTTCCTCTTGTGTTTATTTGAGAATTTTAGGGGCGGGGTTTCACTATTAACTGCCACTGGCTGGC
TGCCCAGTCATGCACTTTCCTTCCTGAGGTCATGGTTTGGCTACATTCCATGGGTTAGCAGCTCCTCCCTGTGGCCTTTTTTTTTATAATATATAAGAGGCCG
AGGCCGCCTCGGCCTCCACCCTTTCTCAGAAGTAGTAAGGGTGTGGAGGCTTTTTCTGAGGCCTAGCAAAAATATTTGGGGAAATCCCTATTCTTTTGCAAT
TTTTGCAAAAATGGATAAAGTGCTTAACAGGGAAGAATCCATGGAGCTCATGGACCTTTTAGGCCTTGAAAGAGCTGCCTGGGGAAACCTTCCCTTAATGA
GAAAAGCCTATTTAAGGAAGTGTAAGGAATTTCACCCTGACAAAGGGGGCGACGAGGATAAAATGAAGAGAATGAATACTTTATATAAAAAAATGAAGC
AGGATGTAAAGGTAGCTCATCAGCCTGATTTTGGAACCTGGAGTAGCTCAGAGGTTTGTGCTGATTTTCCTCTTTGCCCAGATACCCTGTACTGCAAGGATT
GGCCTATTTGTTCCAAAAAGCCTTCTGTGCACTGCCCTTGCATGCTATGTCAGCTTAGATTAAGGCATTTAAATAGAAAATTTTTAAGAAAAGAGCCCTTGG
TTTGGATAGATTGCTACTGCATTGACTGCTTCACACAGTGGTTTGGCTTAGACCTAACTGAAGAAACTCTGCAATGGTGGGTCCAAATAATTGGAGAAACTC
CCTTCAGAGATCTAAAGCTTTAAGGTAACTAACTTATATTTAGATAAATAAGAAAATATTTAAAGGCCCTAAGTAATTATTTTTAAATATATTTAAGAAAATC
CTTACAAAACTCAGAGTTCTTACTTGAAAAAAGAATTTTACAAAGTGGAATGACCTTGTTGCTACTGCTAATTTGGTTTAGGCCTGTAGCTGATTTTGCAACA
GATATACAATCTAGAATTGTTGAATGGAAGGAAAGGCTGGATTCTGAGATAAGTATGTATACTTTTTCAAGGATGAAATATAATATATGCATGGGGAAATG
TATTCTTGATATCACAAGAGAAGAAGATTCAGAAACTGAAGACTCTGGACATGGATCAAGCACTGAATCCCAATCACAATGCTCTTCCCAAGTCTGAGATA
CTTCAGCCCCTGCTGAAGATTCCCAAAGGTCAGACCCCCATAGTCAAGAGTTGCATTTGTGTAAAGGCTTTCAATGTTTTAAAAGGCCTAAAACACCACCCC
CAAAATAACACAAGCTTAAAAGTGGCTTATACAAAAGCAGCATTTATTAAATGTATATGTACAATAAAAGCACCTGTTTAAAGCATTTTGGTTTGCAATTGT
CCTTGTTTATCAATATATCTTATCATATCTGGGTCCCCTGGAAGTCTTTCTGTGCCATCAAACACCCTAACCTCTTCTACCTGGGATTCCATACCATACATAGG
CTGCCCATCCACTCTCTGGGTTCTCCTGTTTATAAGGTCACTCAGCAAAAAGGAAATTGGGTAAGGATTTTTTACAGATCTTTTTCTCAGGCGAATCTTAAAA
TATCTTGCAAGGCCTCTCCACTGTTGTGTTCCAGAGCTGTTAGTAAACAGGCCACAAATATCAGCAGCTGAAACATACAGGCTATCAGCTTTACAAAGAGGC
CCCACACCCTGTTCATCTAGCAACACTGTGGTAGCTGTGTTGGTCACATGAAGTACTGGGGGAACATTTTCCCCTCCTGTGAAAGTCCCAAAATACCTAGTA
TTTTCATTTCTACTGGGATCAGGAACCCAGCACTCAACTGGATAAGCATTGTTTTTGTCCAAATAGGCCTTATGGTCAGTATTCATTACCTGGGACTGGGCTG
TTGGGTTTTTTGGGGTTATAGTACCATCTGGGTACTTGGTCCTGTAATTCATTAGCACTCCCTGCATTTCCAAGGGGTCTCCACCAACAGCAAAGAAGTGGA
AATTACTGCCTTGAATAGGTTTACCTCCACCATGCTCATGCACTTTTTGTGACCCTGCATGAAGGTTAAGCATGCTAGTTATTCCAATGACCTCTGTTTGTACA
GTTACAGCCTCCCACATCAGTAGATTTCCACAGGTTAGGTCCTCATTTAAATTGGGGAGGGGAATTCTTGCTGTGCTGTAACAGGGAAGCATTTTTCTTTCT
GGGCTATCACTGCTAAAGTCATTTTCAGCACTTAGCTTTAGACTAAAGCCCCTAAGGTTTTCATCTGGATCCCCCATTTCTGGGTTTAGGAAGCATTCTACCT
CTGTTATAGCATCTACCCCAGTTTTAACTTCTAGAACTTCTACTCCTCCTTTTATTAGTAGTTTTGGCACTTGCACGGGTTCCTTTGGCTTTTTGGGAGCTGCCC
CTGGACACTCTCCTTTTCTTTTGGTTGGGGCCATCTTCATATGCTTCAAGAGCAGGTGTTACAGTCCCGTACAGGCCTAGAAGTAAAGGCAACATCCATTGA
GGAGCAGTTCTTTGATTAGCACCTCCTGGGGCAATAGTTTTTTCTATAAACTCTCCTGAATGTACAGTTTGTTGATTTCTTAAGTCCATTCTTTGTGTAACTTC
TTCTATACTGTCAGCATCATCTATACTATAAGTATGGCCAAAATGTACACGGGTACCTTCCCTTTCAGCCACTTGTCTAACCATTGAGGGCCTAATAGGGGA
AAGATCAGAATAATATTCTTGAATATAATTATAAAAGTTTATAGGGGCATTTACAATTGTCCAGGTAGTTTCCTCCAAAAATCTAGCCAAGGAGTCTCTAAA
AAATCTTTCTGTTCTTCTTTGCAATTCTTGTGAGGTTATAGCAGGTATATCATCCCTAATAACATGCCACAAAGCCTGGGAAATAGTAGCAAACAAGGAAGG
ACCCCAATGCCTAGGATCAAGGTATTGAATATTATTTACAAAAGTATTTACACCAGGAAACAAAATATCATAGTACTCATCTGGGTTAAACAATTCCAAAGC
CATGCCTGATTGCTGATAGAGGCCTACAGTGGAAACTTTGTGATCCCAATCACTAAAAAACCTATACCCTACTTGAGCCAAGGAACTAATACCAGTAACAGT
TTGAATTAAAGCAGCAAACCCAGCAATAGCCCCAGGAGCACCAGCAATTACAGCATATGTTTGAGGAGTTAGGCCTATAGCAGCTATAGCCTCTGAGGTAC
TTGTTATGCCCTCTACAGTAGCAAGGGATGCAATTTGAACTTCTATAGCAGCAGCAGCCTCCCCAGCAGCAATTTCAGCCACTGAAAATCCTGTGGCAGCA
GCAGCCTCAGATACACTGGCAACTAGGTCCCCCAAAAGTGCTAGAGCAGCACCCATCAACCTGGAAATACAAAAAAAAAGGGATTACTTACCTAGGAGTC
TTTTACAGAGTCTTTTACAGCAGGTAAAGCAGTGGTACTTTTGTTTTTCCCGTCTACACTGTCTTCACCTCTACAAAATTCCAGCAAAAGCTCTAAAATAAAA
ATAAAAATCCTCTGAGCTCTTTTTTTTGTTCCAGTCCAGGTTTTACTAACTTTCACAGAAGCTTGTCGTGACAGCTGGCGCAGAACCATGGCCTTTGTCCAGT
TTAACTGGGGACAAGGCCAAGATTCCTAGGCTCGCAAAACATGTCTGTCTGGCTGCTTTCCACTCCTTTGGCCAGTTTCCACTTCCTCTTGTGTTTATTTGAG
AATTTTAGGGGCGGGGTTTCACTATTAACTGCCACTGGCTGGCTGCCCAGTCATGCACTTTCCTTCCTGAGGTCATGGTTTGGCTACATTCCATGGGTTAGC
AGCTCCTCCCTGTGGCCTTTTTTTTTATAATATATAAGAGGCCGAGGCCGCCTCGGCCTCCACCCTTTCTCAGAAGTAGTAAGGGTGTGGAGGCTTTTTCTG
AGGCCTAGCAAAAATATTTGGGGAAATCCCTATTCTTTTGCAATTTTTGCAAAAATGGATAAAGTGCTTAACAGGGAAGAATCCATGGAGCTCATGGACCT
TTTAGGCCTTGAAAGAGCTGCCTGGGGAAACCTTCCCTTAATGAGAAAAGCCTATTTAAGGAAGTGTAAGGAATTTCACCCTGACAAAGGGGGCGACGAG
GATAAAATGAAGAGAATGAATACTTTATATAAAAAAATGGAGCAGGATGTAAAGGTAGCTCATCAGCCTGATTTTGGAACCTGGAGTAGCTCAGAGGTTT
GTGCTGATTTTCCTCTTTGCCCAGATACCCTGTACTGCAAGGATTGGCCTATTTGTTCCAAAAAGCCTTCTGTGCACTGCCCTTGCATGCTATGTCAGCTTAG
ATTAAGGCATTTAAATAGAAAATTTTTAAGAAAAGAGCCCTTGGTTTGGATAGATTGCTACTGCATTGACTGCTTCACACAGTGGTTTGGCTTAGACCTAAC
TGAAGAAACTCTGCAATGGTGGGTCCAAATAATTGGAGAAACTCCCTTCAGAGATCTAAAGCTTTAAGGTAACTAACTTATATTTAGATAAATAAGAAAAT
ATTTAAAGGCCCTAAGTAATTATTTTTTTTATAGGTGCCAACCTATGGAACAGAAGAGTGGGAGTCCTGGTGGAGCTCCTTTAATGAAAAATGGGATGAAG

ATTTATTTTGCCATGAAGATATGTTTGCCAGTGATGAAGAAGCAACAGCAGATTCTCAACACTCCACACCACCCAAGAAAAAGAGAAAGGTAGAAGACCCT
AAAGACTTTCCTTCTGATCTCTGTCTGGCTGCTTTCCACTCCTTTGGCCAGTTTCCACTTCCTCTTGTGTTTATTTGAGAATTTTAGGGGCGGGGTTTCACTATT
AACTGCCACTGGCTGGCTGCCCAGTCATGCACTTTCCTTCCTGAGGTCATGGTTTGGCTACATTCCATGGGTTAGCAGCTCCTCCCTGTGGCCTTTTTTTTTAT
AATATATAAGAGGCCGAGGCCGCCTCGGCCTCCACCCTTTCTCAGAAGTAGTAAGGGTGTGGAGGCTTTTTCTGAGGCCTAGCAAAAATATTTGGGGAAAT
CCCTATTCTTTTGCAATTTTTGCAAAAATGGATAAAGTGCTTAACAGGGAAGAATCCATGGAGCTCATGGACCTTTTAGGCCTTGAAAGAGCTGCCTGGGG
AAACCTTCCCTTAATGAGAAAAGCCTATTTAAGGAAGTGTAAGGAATTTCACCCTGACAAAGGGGGCGACGAGGATAAAATGAAGAGAATGAATACTTTA
TATAAAAAAATGAAGCAGGATGTAAAGGTAGCTCATCAGCCTGATTTTGGAACCTGGAGTAGCTCAGAGGTTTGTGCTGATTTTCCTCTTTGCCCAGATACC
CTGTACTGCAAGGATTGGCCTATTTGTTCCAAAAAGCCTTCTGTGCACTGCCCTTGCATGCTATGTCAGCTTAGATTAAGGCATTTAAATAGAAAATTTTTAA
GAAAAGAGCCCTTGGTTTGGATAGATTGCTACTGCATTGACTGCTTCACACAGTGGTTTGGCTTAGACCTAACTGAAGAAACTCTGCAATGGTGGGTCCAA
ATAATTGGAGAAACTCCCTTCAGAGATCTAAAGCTTTAAGGTAACTAACTTATATTTAGATAAATAAGAAAATATTTAAAGGCCCTAAGTAATTATTTTTAAA
TATATTTAAGAAAATCCTTACAAAACTCAGAGTTCTTACTTGAAAAAAGAATTTTACAAAGTGGAATGACCTTGTTGCTACTGCTAATTTGGTTTAGGCCTGT
AGCTGATTTTGCAACAGATATACAATCTAGAATTGTTGAATGGAAGGAAAGGCTGGATTCTGAGATAAGTATGTATACTTTTTCAAGGATGAAATATAATA
TATGCATGGGGAAATGTATTCTTGATATCACAAGAGAAGAAGATTCAGAAACTGAAGACTCTGGACATGGATCAAGCACTGAATCCCAATCACAATGCTCT
TCCCAAGTCTGAGATACTTCAGCCCCTGCTGAAGATTCCCAAAGGTCAGACCCCCATAGTCAAGAGTTGCATTTGTGTAAAGGCTTTCAATGTTTTAAAAGG
CCTAAAACACCACCCCCAAAATAACACAAGCTTAAAAGTGGCTTATACAAAAGCAGCATTTATTAAATGTATATGTACAATAAAAGCACCTGTTTAAAGCAT
TTTGGTTTGCAATTGTCCTTGTTTATCAATATATCTTATCATATCTGGGTCCCCTGGAAGTCTTTCTGTGCCATCAAACACCCTAACCTCTTCTACCTGGGATTC
CATACCATACATAGGCTGCCCATCCACTCTCTGGGTTCTCCTGTTTATAAGGTCACTCAGCAAAAAGGAAATTGGGTAAGGATTTTTTACAGATCTTTTTCTC
AGGCGAATCTTAAAATATCTTGCAAGGCCTCTCCACTGTTGTGTTCCAGAGCTGTTAGTAAACAGGCCACAAATATCAGCAGCTGAAACATACAGGCTATCA
GCTTTACAAAGAGGCCCCACACCCTGTTCATCTAGCAACACTGTGGTAGCTGTGTTGGTCACATGAAGTACTGGGGGAACATTTTCCCCTCCTGTGAAAGTC
CCAAAATACCTAGTATTTTCATTTCTACTGGGATCAGGAACCCAGCACTCAACTGGATAAGCATTGTTTTTGTCCAAATAGGCCTTATGGTCAGTATTCATTA
CCTGGGACTGGGCTGTTGGGTTTTTTGGGGTTATAGTACCATCTGGGTACTTGGTCCTGTAATTCATTAGCACTCCCTGCATTTCCAAGGGGTCTCCACCAA
CAGCAAAGAAGTGGAAATTACTGCCTTGAATAGGTTTACCTCCACCATGCTCATGCACTTTTTGTGACCCTGCATGAAGGTTAAGCATGCTAGTTATTCCAA
TGACCTCTGTTTGTACAGTTACAGCCTCCCACATCAGTAGATTTCCACAGGTTAGGTCCTCATTTAAATTGGGGAGGGGAATTCTTGCTGTGCTGTAACAGG
GAAGCATTTTTCTTTCTGGGCTATCACTGCTAAAGTCATTTTCAGCACTTAGCTTTAGACTAAAGCCCCTAAGGTTTTCATCTGGATCCCCCATTTCTGGGTTT
AGGAAGCATTCTACCTCTGTTATAGCATCTACCCCAGTTTTAACTTCTAGAACTTCTACTCCTCCTTTTATTAGTAGTTTTGGCACTTGCACGGGTTCCTTTGG
CTTTTTGGGAGCTGCCCCTGGACACTCTCCTTTTCTTTTGGTTGGGGCCATCTTCATATGCTTCAAGAGCAGGTGTTACAGTCCCGTACAGGCCTAGAAGTA
AAGGCAACATCCATTGAGGAGCAGTTCTTTGATTAGCACCTCCTGGGGCAATAGTTTTTTCTATAAACTCTCCTGAATGTACAGTTTGTTGATTTCTTAAGTC
CATTCTTTGTGTAACTTCTTCTATACTGTCAGCATCATCTATACTATAAGTATGGCCAAAATGTACACGGGTACCTTCCCTTTCAGCCACTTGTCTAACCATTG
AGGGCCTAATAGGGGAAAGATCAGAATAATATTCTTGAATATAATTATAAAAGTTTATAGGGGCATTTACAATTGTCCAGGTAGTTTCCTCCAAAAATCTAG
CCAAGGAGTCTCTAAAAAATCTTTCTGTTCTTCTTTGCAATTCTTGTGAGGTTATAGCAGGTATATCATCCCTAATAACATGCCACAAAGCCTGGGAAATAGT
AGCAAACAAGGAAGGACCCCAATGCCTAGGATCAAGGTATTGAATATTATTTACAAAAGTATTTACACCAGGAAACAAAATATCATAGTACTCATCTGGGT
TAAACAATTCCAAAGCCATGCCTGATTGCTGATAGAGGCCTACAGTGGAAACTTTGTGATCCCAATCACTAAAAAACCTATACCCTACTTGAGCCAAGGAAC
TAATACCAGTAACAGTTTGAATTAAAGCAGCAAACCCAGCAATAGCCCCAGGAGCACCAGCAATTACAGCATATGTTTGAGGAGTTAGGCCTATAGCAGCT
ATAGCCTCTGAGGTACTTGTTATGCCCTCTACAGTAGCAAGGGATGCAATTTGAACTTCTATAGCAGCAGCAGCCTCCCCAGCAGCAATTTCAGCCACTGAA
AATCCTGTGGCAGCAGCAGCCTCAGATACACTGGCAACTAGGTCCCCCAAAAGTGCTAGAGCAGCACCCATCAACCTGGAAATACAAAAAAAAAGGGATT
ACTTACCTAGGAGTCTTTTACAGAGTCTTTTACAGCAGGTAAAGCAGTGGTACTTTTGTTTTTCCCGTCTACACTGTCTTCACCTCTACAAAATTCCAGCAAAA
GCTCTAAAATAAAAATAAAAATCCTCTGAGCTCTTTTTTTTGTTCCAGTCCAGGTTTTACTAACTTTCACAGAAGCTTGTCGTGACAGCTGGCGCAGAACCAT
GGCCTTTGTCCAGTTTAACTGGGGACAAGGCCAAGATTCCTAGGCTCGCAAAACATGTCTGTCTGGCTGCTTTCCACTCCTTTGGCCAGTTTCCACTTCCTCT
TGTGTTTATTTGAGAATTTTAGGGGCGGGGTTTCACTATTAACTGCCACTGGCTGGCTGCCCAGTCATGCACTTTCCTTCCTGAGGTCATGGTTTGGCTACAT
TCCATGGGTTAGCAGCTCCTCCCTGTGGCCTTTTTTTTTATAATATATAAGAGGCCGAGGCCGCCTCGGCCTCCACCCTTTCTCAGAAGTAGTAAGGGTGTG
GAGGCTTTTTCTGAGGCCTAGCAAAAATATTTGGGGAAATCCCTATTCTTTTGCAATTTTTGCAAAAATGGATAAAGTGCTTAACAGGGAAGAATCCATGG
AGCTCATGGACCTTTTAGGCCTTGAAAGAGCTGCCTGGGGAAACCTTCCCTTAATGAGAAAAGCCTATTTAAGGAAGTGTAAGGAATTTCACCCTGACAAA
GGGGGCGACGAGGATAAAATGAAGAGAATGAATACTTTATATAAAAAAATGGAGCAGGATGTAAAGGTAGCTCATCAGCCTGATTTTGGAACCTGGAGT
AGCTCAGAGGTTTGTGCTGATTTTCCTCTTTGCCCAGATACCCTGTACTGCAAGGATTGGCCTATTTGTTCCAAAAAGCCTTCTGTGCACTGCCCTTGCATGC
TATGTCAGCTTAGATTAAGGCATTTAAATAGAAAATTTTTAAGAAAAGAGCCCTTGGTTTGGATAGATTGCTACTGCATTGACTGCTTCACACAGTGGTTTG
GCTTAGACCTAACTGAAGAAACTCTGCAATGGTGGGTCCAAATAATTGGAGAAACTCCCTTCAGAGATCTAAAGCTTTAAGGTAACTAACTTATATTTAGAT
AAATAAGAAAATATTTAAAGGCCCTAAGTAATTATTTTTTTTATAGGTGCCAACCTATGGAACAGAAGAGTGGGAGTCCTGGTGGAGCTCCTTTAATGAAA
AATGGGATGAAGATTTATTTTGCCATGAAGATATGTTTGCCAGTGATGAAGAAGCAACAGCAGATTCTCAACACTCCACACCACCCAAGAAAAAGAGAAA
GGTAGAAGACCCTAAAGACTTTCCTTCTGATCTCTGTCTGGCTGCTTTCCACTCCTTTGGCCAGTTTCCACTTCCTCTTGTGTTTATTTGAGAATTTTAGGGGC
GGGGTTTCACTATTAACTGCCACTGGCTGGCTGCCCAG

DNA sequence of contig 5 as shown in figure 3C)

ATGTATTGCTGGGATGGTGCCAATCACTGACCTTTATCAGAAAAGAAAAATGCCCTAGAATCTCCCATGCGGCACCTGCCTATGCCTCACTGGCATTAAACT
GTGTGCTAAGTCCCACAGTCTCTTCTAGGCATAAAAAAGGAAAAAGAGGCATCAAATCTCAGAGCCTCCCAACTGGAGAGGACAGGGGAGGAGGAGCGA
GGTCAGGTGCTGAGACCTGGAGTGCGCAGCTCCTGTCAAGGTGTCTGTGCTGTTTTATGCAAAAGCATAATTCATGTTTTAAAATCAGTGTGTACAATTAA
GGAAATACACTCACAGAGCTCCTTGCTGTGTGTGTGGATGGTGGGGTGGTCAGGTTCGATTGTACAAAGCTGAGATCCACAAGTGAGCAGAAACATGAGA
TTAAAAAGATGTACAGAGGCCGATTAATTTTAGGTTGTTGGCGTGAGTCATTGCAGCCTAGGGTAGTATCTCATAAATGTGCCCTTTCCCAGGGAATGTTGT
TTTATAAGGAGATTTGTGAACTGATGCACAAAATGTACCAAAAGTCACTGCAAATCAAAGAAAAGCAGCGGCAGAAGAAGTTATTCAGGTCATCTGAGTC
GGGGAGGTGGAAATCATTTCGAACGAGATATTAGAGAAACTGGCACCTTTTGGGGCCTGCAGAAGTCTGTGAAGGACAGAAACGTTGCAAACGTGGATA
TCCCACTTTCAGAGTGAAGTGGAGTGAAGTCACGCGGGAGCCAGGCCTTGGGTGGAGAGTTTATGTTTGTTGTCCCACCTGCTGCTCCCTGAAGTCTTGGG
AGGTGTTTCCCTTGTACCAATGAGGGGCTCAGGGCGCACAGGAGCTCAGTCAGTGCAGCACGTTCGCCGGACGCGGTGAAATATCCCGACCTCTGCTCCA
TCTTGATGGCTAACCCCAAGGACACTTGTTTAAGAAAGAAATGTTTGTGTGGTGGCCTCCACAAACAGGGTGCTGTGTGTAATAGCCACACACAACTGCCT

CCCGGACCAACACAGAGATCCTGTGGACATGAGTGGGAACAGCGAGGCAGCCACACGTTTCCATGCACAATGTGTGCCGTTTCTTTTCCTTTTTTTTTTTTTT
TTTTTTTTGAGACAAGGTCTCTCTCTGTCTCCCAGGCTGGAGTGCAGCGGCGCAGTCTCGGCTCACTGCAACCTCATGTGTGTGTTTGTTTTGTTTTGTTTTG
TTTTTGTCAGTGAAAATAGGCTATGAGCACTCCATGACACTTCTAACCTTCACGAAGTTTCCTTAAAATACTCGCCTTCTTTTTAAAGAAGGAGGGGTTATGG
TTTTCGAAAAAAGTCACAAGCAAACCATAGAAATGATGATTTGCAGGCCAGGTGTGGTGGCTCAGCACTTTGGGAGTCCAAGGAAGGCGGATCACGAGGT
CAGGAGATCGAGACCATCCTGGCCAACATGGTGAAACCCCATCTCTACTAAAAATACAAAAAATTAGCCAGGTGTGGTGGTGGGCGCCTGTAATCCCAGCT
ACTCGGGAGGCTGAGGCAGGAGAATGGCGTAAACCCGGGAGGCGGAGCTGGCAGTGAGCTGGGATCTGCGCCACTGCACTCCATCCAGCCTGGGCGAC
AGAGCGAGACTCCGTCTCAAAAAAAAGGAAAAAAAAAAATCAGATGATTTTCTGGGTTTCAAAATATCCAGCGTCTGGAGCACTCTATCCCCTGGAAAAAT
CTCCTGTCCCCTCTGCTTCCGTGTGGCCGTGGGCGTGGACTGGTTTGTTCATGACACTGATGAATCCTCATTCCTCCTGAATAAGGAGGACGCTTTGTATCTT
TGTGTCTTCGTATGAATAACCTAAGAGCGTTCCTGAAGCCTCGCCTGGCGCTTCTGAGTTGCACGTCTTCTTAGATTTCAGGCTGGCTTGCGGCTTTCCACG
GAGTCTTATCTCTCAGGCCAAAGAGGAGAGTGGGCTCGGGCAGAGCGGCTTCTGCTCTGCATCCGCTGCCCACGCTGAGCCGGGAAAGTTCAGCATCCTC
GCTTCAAATAGAGGCTTTCAAAGGCATTAAAAGAAAAGTCCAGGAAAAGAAAAGGAAACAAATATTGCGCTATTACAAAGAGTCTTTAAGCAAGACATGC
TTATTAGACAGACTTTATTCTCTCTTGCACGTTCAAAGACATCTGATTAAACCATCCAAGGGACATTGACAAAGGCTTTGTCTGTCTGGGCAGGATAGATAA
ACTGTTTCTTGGCAGATTTTGATCATCTCTCAGGCTTGACACCCAGGGAGAAACTCCAATAGCATGTCGTGTGAGGCAGAAGTGTCAGCCACACAGAATCC
AGACCACAGCTTCCTCGGCTACTGTCTGAGGCTCGTCTGCAGGGCAGCTGCAGGTTGGGGCTTGGTGCTGCTGCTGGTGCTTAGAGTGTGTGGGACGATT
TGGTGTGATGCCAGGGACAATCATAGAGACATCGATAAAAACATGGCATAAAGCAAATCTTTTTAAGGAATTAAAAATGTTTTAATTCTTCAGATTTTTCTA
GAAATACAGTTTCAGGTCGACAGAGAAACACTTTTCCCAGCACCCAGGGAGCCTCTGTGTCCCGCTTGTCTGTGTGTACTTGTGTGGTTTTCAGAACTGTCC
TGGGGACCGACCAGTGCAACCAGGACTCTGAGAAACACAGGTTTCCAACACTCAGCCTTTCTGCATTGACCTCGCCATTGTTTTCCCCCTGGCAGCGAATAA
CACGTTGGATCTCTACAGGACTTCTTATCAGGAGGGCTCAACTGACTTACACAGATACCATCTCTCAGATTTTGACAGGCTTCTCCATTTGATATCATCGAAT
AGATGGATATTTTGGGGCAAAAGCGAGTAAAGAACATGCTACACATTATTTTTTCCCACTGCTGATTTATCCACTGGTTTCATTTCCAGTGACGTTAAACATT
CACATTTCTGCACAATTTACTCCTACAGTGGATTGTTCACATCGCGTCCTCATATCCCCACTTTCTTAATTTCGTTATTTTTCCTAGTGCTTTTCATTTCTCATTA
GTTCGGTTTTGTTGAGTCCTACTGATGTAGTCATCACTATTTCTAAATTAAGGAGTCAAGTGATTAAATCGGGCCCATCGTGGTCATCCAAAATAATCTCTCC
ATCTCAAGGCGGTTCATTTAATTGCATCCGCAAAAAATCCCTTTTGCCGCGGAAGGTAACAGATCCACGGGTTCCAGGGATGAGGACATCTTTGTGGAGCC
TTTATTCTGTCGATCACAATAGACTCGAATTACTAATATTTTGTGCATGAGGAACCAAGTCACAGAGAAGTTCAGTATTTCGCTCAAAGCTCCACAGATGGA
TTTCTGTGAACCCAGGCGGGTGACCTCCCCAGAGCCCGCCTTGATAATCACTATTCTATGCTCTAATGGAAAGAAATACCTGACAAGTTTTAAAAGATAGAA
TTGAGTTGTGCCAAATGGTTTGACGAAATACTAACTCTGGAAAGGTCAGGACTGGGTAGCGGTGGCTCACGCCTGTAATCCCAGCACTTTGGGAGGCCGA
GGCGGGTGGATCACCTGAGGTCAGGAGTTCGAGACCAGCCTGGCCAACATGGTGAAACCCCGTCTCTACTAAAAATACAAAAATTAGCCGGGCGTGGTG
GCGGGTGCCTGTAGTCCCAGCTACTCGGGAGGCTGAGGCAGGAGAATCGCTTGAACCCGGGAGGCGGAGGTTGCAGTGAGCCGAGATCGCGCCACTGC
ACTCCAGCCTGGGCGACAGAGCGAGACTCCGTCTCAAAAAAAAAAAAAAAAAAAAAAAAAACTAACTGGATAGGTCAGGAGGTTCTCCAGAAGGAGAACAA
ATGAGTGGACACAAGCTGGTGCAGGAATGGACTGAGGGTCCATGGCCAGGAGGAGCCCCAGGGAGGAAGTGGGCAGATCTGCAGTGGGGGCGTCAAT
ACCGGCCGACCCTCCTAGTGATGCTTCGACTTAAAAGAGGACGCATCAGCCTCCTGCGGGGAGCATGGGGGGATGGTGGCTCTCAGAGCAAAACGTGGG
AGGCATGGAGTACAGGACCCTAAGTCATTTCATGGGGTGGGAGGAAAAACTGTGTGTTTTTGTGTGTGAGAGTGTCTGAGTGTGAGTGTGTGTTTATGAG
TGTGGGTGAGTGTTGTGAATGTGTGTGATTGTGTATGTGAGTGTGAGTGGTGTGTGATTGTGTGTGTATGTGAGTGTGAGTGGTGTGTGAATGTCTGTGT
ATGCGAGTGTGAGTGGTGTGTGATTGTGTGTGTATGGAGTGTGAGTGGTGTGTGATTGTGTGTGTATGTGAGTGTGAGTGGTGTGTGATTGTGTGTGTAT
GTGAGTGCGAGTGGTGTGTGATTGTGTGTGTATGTGAGTGTGAGTGGTGTGTGATTGTGTGTGTATGTGAGTGTGAGTGGTGTGTGATTGTGTGTGTATG
TGAGTGTGAATGGTGTGCGATTGTGTGAATGTTAGCGTGTGAATGTGAATGACTGTGTGGGACTGTGGGTGAGTGGTGTGCATGTGATTGTATGAGTGTG
TGTATGTGTGAGTGGTGTATATGAGTGTGTTTGTACACACTGTGCTGGGTTTGCTGATTCTGCGCGTCTGGCTGGCATCAGATGAGGAAAGGCCGTTCAGG
GCCTGGAGGGAACTGGGGCATGTGCTGAGGTGATCTGACTTACTCTTGCGGGTTAGAGGTAACAAACCAGGTAACTGGCTCACAACTGTGATTCGTTTGC
ATGCTTGAAGTTAACTCAGTATTTTAACTTCATTTAATTAACTTTCAACTTGTAAAAACTCTAGATATTTTAATTTAAAAATTCACATTACTAGTATTTCTTGAG
AAATAAAAAAAAAAAGAATGTGGCCATTGTAGGTCCAGATGTTTCCTGGACAGTACTTGACAGGTGTTGCGTAGCTGCCTCCTTCTTTGGGCTGAAGAAACG
CTCCTGGCTTGCACGGTTTTCTTCCTCCACACTGTCCCCCCAGCAGTGAGGCCAAGGGTTACTTGCTAGTTGCCATTGAACTTAGGCTGTTGTTTTTACACAC
CCTGTTCATCTAGCAACACTGTGGTAGCTGTGTTGGTCACATGAAGTACTGGGGGAACATTTTCCCCTCCTGTGAAAGTCCCAAAATACCTAGTATTTTCATT
TCTACTGGGATCAGGAACCCAGCACTCAACTGGATAAGCATTGTTTTTGTCCAAATAGGCCTTATGGTCAGTATTCATTACCTGGGACTGGGCTGTTGGGTT
TTTTGGGGTTATAGTACCATCTGGGTACTTGGTCCTGTAATTCATTAGCACTCCCTGCATTTCCAAGGGGTCTCCACCAACAGCAAAGAAGTGGAAATTACT
GCCTTGAATAGGTTTACCTCCACCATGCTCATGCACTTTTTGTGACCCTGCATGAAGGTTAAGCATGCTAGTTATTCCAATGACCTCTGTTTGTACAGTTACA
GCCTCCCACATCAGTAGATTTCCACAGGTTAGGTCCTCATTTAAATTGGGGAGGGGAATTCTTGCTGTGCTGTAACAGGGAAGCATTTTTCTTTCTGGGCTA
TCACTGCTAAAGTCATTTTCAGCACTTAGCTTTAGACTAAAGCCCCTAAGGTTTTCATCTGGATCCCCCATTTCTGGGTTTAGGAAGCATTCTACCTCTGTTAT
AGCATCTACCCCAGTTTTAACTTCTAGAACTTCTACTCCTCCTTTTATTAGTAGTTTTGGCACTTGCACGGGTTCCTTTGGCTTTTTGGGAGCTGCCCCTGGAC
ACTCTCCTTTTCTTTTGGTTGGGGCCATCTTCATATGCTTCAAGAGCAGGTGTTACAGTCCCGTACAGGCCTAGAAGTAAAGGCAACATCCATTGAGGAGCA
GTTCTTTGATTAGCACCTCCTGGGGCAATAGTTTTTTCTATAAACTCTCCTGAATGTACAGTTTGTTGATTTCTTAAGTCCATTCTTTGTGTAACTTCTTCTATA
CTGTCAGCATCATCTATACTATAAGTATGGCCAAAATGTACACGGGTACCTTCCCTTTCAGCCACTTGTCTAACCATTGAGGGCCTAATAGGGGAAAGATCA
GAATAATATTCTTGAATATAATTATAAAAGTTTATAGGGGCATTTACAATTGTCCAGGTAGTTTCCTCCAAAAATCTAGCCAAGGAGTCTCTAAAAAATCTTT
CTGTTCTTCTTTGCAATTCTTGTGAGGTTATAGCAGGTATATCATCCCTAATAGTATGTCATAATGCTCTGGTACATTTTATCCATCAAGTCAGGCCCCCTTTG
TCTAGGGTGAAATTCCTTGACACATTCCTTACAATAGGCTATTTACATCATTAAGCGGAAGGTTTCCCCAGGCAGCTCTTTCAAGGCCTAAAAGGTCCATGA
GCCTCCATGGATTCTGATCCCTCGTTAAGCACTTTATCCATTTTTGCAAAAATTGCAAAAGAATAGGGATTTCCCCAAATATTTTTGCTAGGCCTCAGAAAAA
GCCTCCACACCCTTACTACTTCTGAGAAAGGGTGGAGGCCGAGGCGGCCTCGGCCTCTTATATATTATAAAAAAAAAGGCCACAGGGAGGAGCTGCTAAC
CCATGGAATGTAGCCAAACCATGACCTCAGGAAGGAAAGTGCATGACTGGGCAGCCAGCCAGTGGCAGTTAATAGTGAAACCCCGCACGGCCTAATATGT
CTCAGATTAACATAAGAGGACAGTGGCCAAGACTGGCCATAGGCGTCGGAAAGCATGCCAGACAGACATGCTTTGCGACGCCTTTGGCCAGTTTCCACTT
GCCTCTTGTGTCTAGTTGAGAATTCTAGGGGCAAGGGTTTCACTATTAACTGCCACTGGCTGGCTGCCCAGTCATGCACTTTCCTTCCTGAGGTCATGGTTT
GGCTACATTCCATGGGTTAGCAGCTCCTCCCTGTGGCCTTTTTTTTTATAATATATAAGAGGCCGAGGCCGCCTCGGCCTCCACCCTTTCTCAGAAGTAGTAA
GGGTGTGGAGGCTTTTTCTGAGGCCTAGCAAAAATATTTGGGGAAATCCCTATTCTTTTGCAATTTTTGCAAAAATGGATAAAGTGCTTAACAGGGAAGAA
TCCATGGAGCTCATGGACCTTTTAGGCCTTGAAAGAGCTGCCTGGGGAAACCTTCCCTTAATGAGAAAAGCCTATTTAAGGAAGTGTAAGGAATTTCACCC
TGACAAAGGGGGCGACGAGGATAAAATGAAGAGAATGAATACTTTATATAAAAAAATGGAGCAGGATGTAAAGGTAGCTCATCAGCCTGATTTTGGAAC
CTGGAGTAGCTCAGAGGTTTGTGCTGATTTTCCTCTTTGCCCAGATACCCTGTACTGCAAGGATTGGCCTATTTGTTCCAAAAAGCCTTCTGTGCACTGCCCT
TGCATGCTATGTCAGCTTAGATTAAGGCATTTAAATAGAAAATTTTTAAGAAAAGAGCCCTTGGTTTGGATAGATTGCTACTGCATTGACTGCTTCACACAG
TGGTTTGGCTTAGACCTAACTGAAGAAACTCTGCAATGGTGGGTCCAAATAATTGGAGAAACTCCCTTCAGAGATCTAAAGCTTTAAGGTAACTAACTTATA
TTTAGATAAATAAGAAAATATTTAAAGGCCCTAAGTAATTATTTTTTTTATAGGTGCCAACCTATGGAACAGAAGAGTGGGAGTCCTGGTGGAGCTCCTTTA
ATGAAAAATGGGATGAAGATTTATTTTGCCATGAAGATATGTTTGCCAGTGATGAAGAAGCAACAGCAGATTCTCAACACTCCACACCACCCAAGAAAAAG

AGAAAGGTAGAAGACCCTAAAGACTTTCCTTCTGATCTACACCAGTTTCTTAGTCAAGCTGTATTTAGTAATAGAACCCTTGCCTGCTTTGCTGTGTATACTA
CTAAAGAAAAAGCTCAAATTCTGTATAAAAAACTTATGGAAAAATATTCTGTAACTTTTATTAGTAGACACATGTGTGCTGGGCATAATATTATATTCTTTTT
AACTCCACATAGACATAGAGTTTCTGCAATTAATAATTTCTGTCAAAAGCTGTGTACCTTTAGTTTTTTAATTTGTAAGGGTGTTAATAAGGAATACTTACTA
TATAGTGCCTTAACTAGAGATCCATACCATACAATAGAAGAAAGCATTCAAGGGGGCTTAAAGGAGCATGATTTTAACCCAGAAGAGCCTGAAGAAACAA
AGCAGGTGTCTTGGAAATTAATTACTGAGTATGCAGTAGAGACAAAGTGTGAGGATGTGTTCTTATTATTGGGTATGTATTTAGAATTTCAATACAATGTA
GAAGAGTGTAAAAAGTGTCAGAAAAAAGACCAACCTTATCACTTTAAGTATCATGAAAAGCATTTTGCAAATGCTACTATTTTTGCAGAAAGTAAAAATCA
AAAAAGTATTTGTCAGCAAGCAGTAGATACAGTTTTAGCTAAAAAAAGAGTAGATACCCTTCATATGACCAGAGAAGAAATGCTAACAGAAAGATTTAATC
ATATATTAGATAAAATGGATTTAATATTTGGAGCTCATGGAAATGCTGTACTGGAACAATATATGGCAGGTGTTGCTTGGCTGCACTGTTTGCTACCTAAAA
TGGATTCTGTTATATTTGATTTTTTGCACTGTATTGTTTTTAATGTACCTAAAAGAAGATACTGGTTATTTAAAGGTCCCATTGATAGTGGAAAAACAACACT
AGCTGCAGGGTTATTAGATTTGTGTGGAGGTAAAGCCTTAAATGTAAACCTACCCATGGAAAGGCTAACCTTTGAACTAGGTGTAGCTATAGATCAGTACA
TGGTTGTTTTTGAAGATGTAAAAGGAACTGGGGCTGAATCAAAGGATCTGCCTTCAGGACATGGAATAAACAATTTAGACAGTTTGAGAGATTATTTAGAT
GGAAGTGTTAAGGTTAATTTAGAAAAGAAACATTTAAACAAAAGAACCCAAATATTTCCACCAGGCCTGGTTACAATGAATGAGTATCCTGTTCCTAAAAC
CCTGCAGGCTAGATTTGTAAGACAGATAGATTTTAGACCCAAAATATATTTAAGAAAATCCTTACAAAACTCAGAGTTCTTACTTGAAAAAAGAATTTTACA
AAGTGGAATGACCTTGTTGCTACTGCTAATTTGGTTTAGGCCTGTAGCTGATTTTGCAACAGATATACAATCTAGAATTGTTGAATGGAAGGAAAGGCTGG
ATTCTGAGATAAGTATGTATACTTTTTCAAGGATGAAATATAATATATGCATGGGGAAATGTATTCTTGATATCACAAGAGAAGAAGATTCAGAAACTGAA
GACTCTGGACATGGATCAAGCACTGAATCCCAATCACAATGCTCTTCCCAAGTCTCAGATACTTCAGCCCCTGCTGAAGATTCCCAAAGGTCAGACCCCCAT
AGTCAAGAGTTGCATTTGTGTAAAGGCTTTCAATGTTTTAAAAGGCCTAAAACACCACCCCCAAAATAACACAAGCTTAAAAGTGGCTTATACAAAAGCAG
CATTTATTAAATGTATATGTACAATAAAAGCACCTGTTTAAAGCATTTTGGTTTGCAATTGTCCTTGTTTATCAATATATCTTATCATATCTGGGTCCCCTGGA
AGTCTTTCTGTGCCATCAAACACCCTAACCTCTTCTACCTGGGATTCCATACCATACATAGGCTGCCCATCCACTCTCTGGGTTCTCCTGTTTATAAGGTCACT
CAGCAAAAAGGAAATTGGGTAAGGATTTTTTACAGATCTTTTTCTCAGGCGAATCTTAAAATATCTTGCAAGGCCTCTCCACTGTTGTGTTCCAGAGCTGTT
AGTAAACAGGCCACAAATATCAGCAGCTGAAACATACAGGCTATCAGCTTTACAAAGAGGCCCCACACCCTGTTCATCTAGCAACACTGTGGTAGCTGTGT
TGGTCACATGAAGTACTGGGGGAACATTTTCCCCTCCTGTGAAAGTCCCAAAATACCTAGTATTTTCATTTCTACTGGGATCAGGAACCCAGCACTCAACTG
GATAAGCATTGTTTTTGTCCAAATAGGCCTTATGGTCAGTATTCATTACCTGGGACTGGGCTGTTGGGTTTTTTGGGGTTATAGTACCATCTGGGTACTTGG
TCCTGTAATTCATTAGCACTCCCTGCATTTCCAAGGGGTCTCCACCAACAGCAAAGAAGTGGAAATTACTGCCTTGAATAGGTTTACCTCCACCATGCTCATG
CACTTTTTGTGACCCTGCATGAAGGTTAAGCATGCTAGTTATTCCAATGACCTCTGTTTGTACAGTTACAGCCTCCCACATCAGTAGATTTCCACAGGTTAGG
TCCTCATTTAAATTGGGGAGGGGAATTCTTGCTGTGCTGTAACAGGGAAGCATTTTTCTTTCTGGGCTATCACTGCTAAAGTCATTTTCAGCACTTAGCTTTA
GACTAAAGCCCCTAAGGTTTTCATCTGGATCCCCCATTTCTGGGTTTAGGAAGCATTCTACCTCTGTTATAGCATCTACCCCAGTTTTAACTTCTAGAACTTCT
ACTCCTCCTTTTATTAGTAGTTTTGGCACTTGCACGGGTTCCTTTGGCTTTTTGGGAGCTGCCCCTGGACACTCTCCTTTTCTTTTGGTTGGGGCCATCTTCAT
ATGCTTCAAGAGCAGGTGTTACAGTCCCGTACAGGCCTAGAAGTAAAGGCAACATCCATTGAGGAGCAGTTCTTTGATTAGCACCTCCTGGGGCAATAGTT
TTTTCTATAAACTCTCCTGAATGTACAGTTTGTTGATTTCTTAAGTCCATTCTTTGTGTAACTTCTTTTATACTGTCAGCATCATCTATACTATAAGTATGGCCA
AAATGTACACGGGTACCTTCCCTTTCAGCCACTTGTCTAACCATTGAGGGCCTAATAGGGGAAAGATCAGAATAATATTCTTGAATATAATTATAAAAGTTT
ATAGGGGCATTTACAATTGTCCAGGTAGTTTATCCATTTTTGCAAAAATTGCAAAAGAATAGGGATTTCCCCAAATATTTTTGCTAGGCCTCAGAAAAAGCC
TCCACACCCTTACTACTTCTGAGAAAGGGTGGAGGCCGAGGCGGCCTCGGCCTCTTATATATTATAAAAAAAAAGGCCACAGGGAGGAGCTGCTAACCCA
TGGAATGTAGCCAAACCATGACCTCAGGAAGGAAAGTGCATGACTGGGCAGCCAGCCAGTGGCAGTTAATAGTGAAACCCCGCCCCTAAAATTCTCAAAT
AAACACAAGAGGAAGTGGAAACTGGCCAAAGGAGTGGAAAGCAGCCAGACAGACATGTTTTGCGAGCCTAGGAATCTTGGCCTTGTCCCCAGTTAAACT
GGACAAAGGCCTGCCACTGGCTGGCTGCCCAGTCATGCACTTTCCTTCCTGAGGTCATGGTTTGGCTACATTCCATGGGTTAGCAGCTCCTCCCTGTGGCCT
TTTTTTTTATAATATATAAGAGGCCGAGGCCGCCTCGGCCTCCACCCTTTCTCAGAAGTAGTAAGGGTGTGGAGGCTTTTTCTGAGGCCTAGCAAAAATATT
TGGGGAAATCCCTATTCTTTTGCAATTTTTGCAAAAATGGATAAAGTGCTTAACAGGGAAGAATCCATGGAGCTCATGGACCTTTTAGGCCTTGAAAGAGC
TGCCTGGGGAAACCTTCCCTTAATGAGAAAAGCCTATTTAAGGAAGTGTAAGGAATTTCACCCTGACAAAGGGGGCGACGAGGATAAAATGAAGAGAAT
GAATACTTTATATAAAAAAATGGAGCAGGATGTAAAGGTAGCTCATCAGCCTGATTTTGGAACCTGGAGTAGCTCAGAGGTTTGTGCTGATTTTCCTCTTTG
CCCAGATACCCTGTACTGCAAGGATTGGCCTATTTGTTCCAAAAAGCCTTCTGTGCACTGCCCTTGCATGCTATGTCAGCTTAGATTAAGGCATTTAAATAGA
AAATTTTTAAGAAAAGAGCCCTTGGTTTGGATAGATTGCTACTGCATTGACTGCTTCACACAGTGGTTTGGCTTAGACCTAACTGAAGAAACTCTGCAATGG
TGGGTCCAAATAATTGGAGAAACTCCCTTCAGAGATCTAAAGCTTTAAGGTAACTAACTTATATTTAGATAAATAAGAAAATATTTAAAGGCCCTAAGTAAT
TATTTTTTTTATAGGTGCCAACCTATGGAACAGAAGAGTGGGAGTCCTGGTGGAGCTCCTTTAATGAAAAATGGGATGAAGATTTATTTTGCCATGAAGAT
ATGTTTGCCAGTGATGAAGAAGCAACAGCAGATTCTCAACACTCCACACCACCCAAGAAAAAGAGAAAGGTAGAAGACCCTAAAGACTTTCCTTCTGATCT
ACACCAGTTTCTTAGTCAAGCTGTATTTAGTAATAGAACCCTTGCCTGCTTTGCTGTGTATACTACTAAAGAAAAAGCTCAAATTCTGTATAAAAAACTTATG
GAAAAATATTCTGTAACTTTTATTAGTAGACACATGTGTGCTGGGCATAATATTATATTCTTTTTAACTCCACATAGACATAGAGTTTCTGCAATTAATAATTT
CTGTCAAAAGCTGTGTACCTTTAGTTTTTTAATTTGTAAGGGTGTTAATAAGGAATACTTACTATATAGTGCCTTAACTAGAGATCCATACCATACAATAGAA
GAAAGCATTCAAGGGGGCTTAAAGGAGCATGATTTTAACCCAGAAGAGCCTGAAGAAACAAAGCAGGTGTCTTGGAAATTAATTACTGAGTATGCAGTAG
AGACAAAGTGTGAGGATGTGTTCTTATTATTGGGTATGTATTTAGAATTTCAATACAATGTAGAAGAGTGTAAAAAGTGTCAGAAAAAAGACCAACCTTAT
CACTTTAAGTATCATGAAAAGCATTTTGCAAATGCTACTATTTTTGCAGAAAGTAAAAATCAAAAAAGTATTTGTCAGCAAGCAGTAGATACAGTTTTAGCT
AAAAAAAGAGTAGATACCCTTCATATGACCAGAGAAGAAATGCTAACAGAAAGATTTAATCATATATTAGATAAAATGGATTTAATATTTGGAGCTCATGG
AAATGCTGTACTGGAACAATATATGGCAGGTGTTGCTTGGCTGCACTGTTTGCTACCTAAAATGGATTCTGTTATATTTGATTTTTTGCACTGTATTGTTTTTA
ATGTACCTAAAAGAAGATACTGGTTATTTAAAGGTCCCATTGATAGTGGAAAAACAACACTAGCTGCAGGGTTATTAGATTTGTGTGGAGGTAAAGCCTTA
AATGTAAACCTACCCATGGAAAGGCTAACCTTTGAACTAGGTGTAGCTATAGATCAGTACATGGTTGTTTTTGAAGATGTAAAAGGAACTGGGGCTGAATC
AAAGGATCTGCCTTCAGGACATGGAATAAACAATTTAGACAGTTTGAGAGATTATTTAGATGGAAGTGTTAAGGTTAATTTAGAAAAGAAACATTTAAACA
AAAGAACCCAAATATTTCCACCAGGCCTGGTTACAATGAATGAGTATCCTGTTCCTAAAACCCTGCAGGCTAGATTTGTAAGACAGATAGATTTTAGACCCA
AAATATATTTAAGAAAATCCTTACAAAACTCAGAGTTCTTACTTGAAAAAAGAATTTTACAAAGTGGAATGACCTTGTTGCTACTGCTAATTTGGTTTAGGCC
TGTAGCTGATTTTGCAACAGATATACAATCTAGAATTGTTGAATGGAAGGAAAGGCTGGATTCTGAGATAAGTATGTATACTTTTTCAAGGATGAAATATA
ATATATGCATGGGGAAATGTATTCTTGATATCACAAGAGAAGAAGATTCAGAAACTGAAGACTCTGGACATGGATCAAGCACTGAATCCCAATCACAATGC
TCTTCCCAAGTCTCAGATACTTCAGCCCCTGCTGAAGATTCCCAAAGGTCAGACCCCCATAGTCAAGAGTTGCATTTGTGTAAAGGCTTTCAATGTTTTAAAA
GGCCTAAAACACCACCCCCAAAATAACACAAGCTTAAAAGTGGCTTATACAAAAGCAGCATTTATTAAATGTATATGTACAATAAAAGCACCTGTTTAAAGC
ATTTTGGTTTGCAATTGTCCTTGTTTATCAATATATCTTATCATATCTGGGTCCCCTGGAAGTCTTTCTGTGCCATCAAACACCCTAACCTCTTCTACCTGGGAT
TCCATACCATACATAGGCTGCCCATCCACTCTCTGGGTTCTCCTGTTTATAAGGTCACTCAGCAAAAAGGAAATTGGGTAAGGATTTTTTACAGATCTTTTTC
TCAGGCGAATCTTAAAATATCTTGCAAGGCCTCTCCACTGTTGTGTTCCAGAGCTGTTAGTAAACAGGCCACAAATATCAGCAGCTGAAACATACAGGCTAT
CAGCTTTACAAAGAGGCCCCACACCCTGTTCATCTAGCAACACTGTGGTAGCTGTGTTGGTCACATGAAGTACTGGGGGAACATTTTCCCCTCCTGTGAAAG
TCCCAAAATACCTAGTATTTTCATTTCTACTGGGATCAGGAACCCAGCACTCAACTGGATAAGCATTGTTTTTGTCCAAATAGGCCTTATGGTCAGTATTCAT

TACCTGGGACTGGGCTGTTGGGTTTTTTGGGGTTATAGTACCATCTGGGTACTTGGTCCTGTAATTCATTAGCACTCCCTGCATTTCCAAGGGGTCTCCACC
AACAGCAAAGAAGTGGAAATTACTGCCTTGAATAGGTTTACCTCCACCATGCTCATGCACTTTTTGTGACCCTGCATGAAGGTTAAGCATGCTAGTTATTCC
AATGACCTCTGTTTGTACAGTTACAGCCTCCCACATCAGTAGATTTCCACAGGTTAGGTCCTCATTTAAATTGGGGAGGGGAATTCTTGCTGTGCTGTAACA
GGGAAGCATTTTTCTTTCTGGGCTATCACTGCTAAAGTCATTTTCAGCACTTAGCTTTAGACTAAAGCCCCTAAGGTTTTCATCTGGATCCCCCATTTCTGGG
TTTAGGAAGCATTCTACCTCTGTTATAGCATCTACCCCAGTTTTAACTTCTAGAACTTCTACTCCTCCTTTTATTAGTAGTTTTGGCACTTGCACGGGTTCCTTT
GGCTTTTTGGGAGCTGCCCCTGGACACTCTCCTTTTCTTTTGGTTGGGGCCATCTTCATATGCTTCAAGAGCAGGTGTTACAGTCCCGTACAGGCCTAGAAG
TAAAGGCAACATCCATTGAGGAGCAGTTCTTTGATTAGCACCTCCTGGGGCAATAGTTTTTTCTATAAACTCTCCTGAATGTACAGTTTGTTGATTTCTTAAG
TCCATTCTTTGTGTAACTTCTTCTATACTGTCAGCATCATCTATACTATAAGTATGGCCAAAATGTACACGGGTACCTTCCCTTTCAGCCACTTGTCTAACCATT
GAGGGCCTAATAGGGGAAAGATCAGAATAATATTCTTGAATATAATTATAAAAGTTTATAGGGGCATTTACAATTGTCCAGGTAGTTTCCTCCAAAAATCTA
GCCAAGGAGTCTCTAAAAAATCTTTCTGTTCTTCTTTGCAATTCTTGTGAGGTTATAGCAGGTATATCATCCCTAAAGTATTCATTCTCTTCATTTTATCCTCGT
CGCCCCCTTTGTCAGGGTGAAATTCCTTACACTTCCTTAAATAGGCTTTTCTCATTAAGGGAAGGTTTCCCCAGGCAGCTCTTTCAAGGCCTAAAAGGTCCAT
GAGCTCCATGGATTCTATCCCTGTTAAGCACTTTATCCATTTTTGCAAAAATTGCAAAAGAATAGGGATTTCCCCAAATATTTTTGCTAGGCCTCAGAAAAAG
CCTCCACACCCTTACTACTTCAGAGAAAGGGTGGAGGCCGAGGCGGCCTCGGCCTCTTATATATTATAAAAAAAAAGGCCACAGGGAGGAGCTGCTAACC
CATGGAATGTAGCCAAACCATGACCTCAGGAAGGAAAGTGCATGACTGGGCAGCCAGCCAGTGGCAGTTAATAGTGAAACTGCGCCCATGGCCTTTGTCC
AGTTTAACTGGGGACAAGGCCAAGATTCCTAGGCTCGCAAAACATGTCTGTCTGGCTGCTTTCCACTCCTTTGGCCAGTTTCCACTTCCTCTTGTGTTTATTT
GAGAATTTTAGGGGCGGGGTTTCACTATTAACTGCCACTGGCTGGCTGCCCAGTCATGCACTTTCCTTCCTGAGGTCATGGTTTGGCTACATTCCATGGGTT
AGCAGCTCCTCCCTGTGGCCTTTTTTTTTATAATATATAAGAGGCCGAGGCCGCCTCGGCCTCCACCCTTTCTCAGAAGTAGTAAGGGTGTGGAGGCTTTTT
CTGAGGCCTAGCAAAAATATTTGGGGAAATCCCTATTCTTTTGCAATTTTTGCAAAAATGGATAAAGTGCTTAACAGGGAAGAATCCATGGAGCTCATGGA
CCTTTTAGGCCTTGAAAGAGCTGCCTGGGGAAACCTTCCCTTAATGAGAAAAGCCTATTTAAGGAAGTGTAAGGAATTTCACCCTGACAAAGGGGGCGAC
GAGGATAAAATGAAGAGAATGAATACTTTATATAAAAAAATGGAGCAGGATGTAAAGGTAGCTCATCAGCCTGATTTTGGAACCTGGAGTAGCTCAGAGG
TTTGTGCTGATTTTCCTCTTTGCCCAGATACCCTGTACTGCAAGGATTGGCCTATTTGTTCCAAAAAGCCTTCTGTGCACTGCCCTTGCATGCTATGTCAGCTT
AGATTAAGGCATTTAAATAGAAAATTTTTAAGAAAAGAGCCCTTGGTTTGGATAGATTGCTACTGCATTGACTGCTTCACACAGTGGTTTGGCTTAGACCTA
ACTGAAGAAACTCTGCAATGGTGGGTCCAAATAATTGGAGAAACTCCCTTCAGAGATCTAAAGCTTTAAGGTAACTAACTTATATTTAGATAAATAAGAAA
ATATTTAAAGGCCCTAAGTAATTATTTTTTTTATAGGTGCCAACCTATGGAACAGAAGAGTGGGAGTCCTGGTGGAGCTCCTTTAATGAAAAATGGGATGA
AGATTTATTTTGCCATGAAGATATGTTTGCCAGTGATGAAGAAGCAACAGCAGATTCTCAACACTCCACACCACCCAAGAAAAAGAGAAAGGTAGAAGAC
CCTAAAGACTTTCCTTCTGATCTACACCAGTTTCTTAGTCAAGCTGTATTTAGTAATAGAACCCTTGCCTGCTTTGCTGTGTATACTACTAAAGAAAAAGCTCA
AATTCTGTATAAAAAACTTATGGAAAAATATTCTGTAACTTTTATTAGTAGACACATGTGTGCTGGGCATAATATTATATTCTTTTTAACTCCACATAGACATA
GAGTTTCTGCAATTAATAATTTCTGTCAAAAGCTGTGTACCTTTAGTTTTTTAATTTGTAAGGGTGTTAATAAGGAATACTTACTATATAGTGCCTTAACTAG
AGATCCATACCATACAATAGAAGAAAGCATTCAAGGGGGCTTAAAGGAGCATGATTTTAACCCAGAAGAGCCTGAAGAAACAAAGCAGGTGTCTTGGAAA
TTAATTACTGAGTATGCAGTAGAGACAAAGTGTGAGGATGTGTTCTTATTATTGGGTATGTATTTAGAATTTCAATACAATGTAGAAGAGTGTAAAAAGTG
TCAGAAAAAAAGACCAACCTTATCACTTTAAGTATCATGAAAAGCATTTTGCAAATGCTACTATTTTTGCAGAAAGTAAAAATCAAAAAAGTATTTGTCAGCA
AGCAGTAGATACAGTTTTAGCTAAAAAAAGAGTAGATACCCTTCATATGACCAGAGAAGAAATGCTAACAGAAAGATTTAATCATATATTAGATAAAATGG
ATTTAATATTTGGAGCTCATGGAAATGCTGTACTGGAACAATATATGGCAGGTGTTGCTTGGCTGCACTGTTTGCTACCTAAAATGGATTCTGTTATATTTG
ATTTTTTGCACTGTATTGTTTTTAATGTACCTAAAAGAAGATACTGGTTATTTAAAGGTCCCATTGATAGTGGAAAAACAACACTAGCTGCAGGGTTATTAG
ATTTGTGTGGAGGTAAAGCCTTAAATGTAAACCTACCCATGGAAAGGCTAACCTTTGAACTAGGTGTAGCTATAGATCAGTACATGGTTGTTTTTGAAGAT
GTAAAAGGAACTGGGGCTGAATCAAAGGATCTGCCTTCAGGACATGGAATAAACAATTTAGACAGTTTGAGAGATTATTTAGATGGAAGTGTTAAGGTTA
ATTTAGAAAAGAAACATTTAAACAAAAGAACCCAAATATTTCCACCAGGCCTGGTTACAATGAATGAGTATCCTGTTCCTAAAACCCTGCAGGCTAGATTTG
TAAGACAGATAGATTTTAGACCCAAAATATATTTAAGAAAATCCTTACAAAACTCAGAGTTCTTACTTGAAAAAAGAATTTTACAAAGTGGAATGACCTTGT
TGCTACTGCTAATTTGGTTTAGGCCTGTAGCTGATTTTGCAACAGATATACAATCTAGAATTGTTGAATGGAAGGAAAGGCTGGATTCTGAGATAAGTATG
TATACTTTTTCAAGGATGAAATATAATATATGCATGGGGAAATGTATTCTTGATATCACAAGAGAAGAAGATTCAGAAACTGAAGACTCTGGACATGGATC
AAGCACTGAATCCCAATCACAATGCTCTTCCCAAGTCTCAGATACTTCAGCCCCTGCTGAAGATTCCCAAAGGTCAGACCCCCATAGTCAAGAGTTGCATTT
GTGTAAAGGCTTTCAATGTTTTAAAAGGCCTAAAACACCACCCCCAAAATAACACAAGCTTAAAAGTGGCTTATACAAAAGCAGCATTTATTAAATGTATAT
GTACAATAAAAGCACCTGTTTAAAGCATTTTGGTTTGCAATTGTCCTTGTTTATCAATATATCTTATCATATCTGGGTCCCCTGGAAGTCTTTCTGTGCCATCA
AACACCCTAACCTCTTCTACCTGGGATTCCATACCATACATAGGCTGCCCATCCACTCTCTGGGTTCTCCTGTTTATAAGGTCACTCAGCAAAAAGGAAATTG
GGTAAGGATTTTTTACAGATCTTTTTCTCAGGCGAATCTTAAAATATCTTGCAAGGCCTCTCCACTGTTGTGTTCCAGAGCTGTTAGTAAACAGGCCACAAAT
ATCAGCAGCTGAAACATACAGGCTATCAGCTTTACAAAGAGGCCCCACACCCTGTTCATCTAGCAACACTGTGGTAGCTGTGTTGGTCACATGAAGTACTG
GGGGAACATTTTCCCCTCCTGTGAAAGTCCCAAAATACCTAGTATTTTCATTTCTACTGGGATCAGGAACCCAGCACTCAACTGGATAAGCATTGTTTTTGTC
CAAATAGGCCTTATGGTCAGTATTCATTACCTGGGACTGGGCTGTTGGGTTTTTTGGGGTTATAGTACCATCTGGGTACTTGGTCCTGTAATTCATTAGCAC
TCCCTGCATTTCCAAGGGGTCTCCACCAACAGCAAAGAAGTGGAAATTACTGCCTTGAATAGGTTTACCTCCACCATGCTCATGCACTTTTTGTGACCCTGCA
TGAAGGTTAAGCATGCTAGTTATTCCAATGACCTCTGTTTGTACAGTTACAGCCTCCCACATCAGTAGATTTCCACAGGTTAGGTCCTCATTTAAATTGGGGA
GGGGAATTCTTGCTGTGCTGTAACAGGGAAGCATTTTTCTTTCTGGGCTATCACTGCTAAAGTCATTTTCAGCACTTAGCTTTAGACTAAAGCCCCTAAGGTT
TTCATCTGGATCCCCCATTTCTGGGTTTAGGAAGCATTCTACCTCTGTTATAGCATCTACCCCAGTTTTAACTTCTAGAACTTCTACTCCTCCTTTTATTAGTAG
TTTTGGCACTTGCACGGGTTCCTTTGGCTTTTTGGGAGCTGCCCCTGGACACTCTCCTTTTCTTTTGGTTGGGGCCATCTTCATATGCTTCAAGAGCAGGTGT
TACAGTCCCGTACAGGCCTAGAAGTAAAGGCAACATCCATTGAGGAGCAGTTCTTTGATTAGCACCTCCTGGGGCAATAGTTTTTTCTATAAACTCTCCTGA
ATGTACAGTTTGTTGATTTCTTAAGTCCATTCTTTGTGTAACTTCTTCTATACTGTCAGCATCATCTATACTATAAGTATGGCCAAAATGTACACGGGTACCTT
CCCTTTCAGCCACTTGTCTAACCATTGAGGGCCTAATAGGGGAAAGATCAGAATAATATTCTTGAATATAATTATAAAAGTTTATAGGGGCATTTACAATTG
TCCAGGTAGTTTCCTCCAAAAATCTAGCCAAGGAGTCTCTAAAAAATCTTTCTGTTCTTCTTTGCAATTCTTGTGAGGTTATAGCAGGTATATCATCCCTAATA
ACATGCCACAAAGCCTGGGAAATAGTAGCAAACAAGGAAGGACCCCAATGCCTAGGATCAAGGTATTGAATATTATTTACAAAAGTATTTACACCAGGAA
ACAAAATATCATAGTACTCATCTGGGTTAAACAATTCCAAAGCCATGCCTGATTGCTGATAGAGGCCTACAGTGGAAACTTTGTGATCCCAATCACTAAAAA
ACCTATACCCTACTTGAGCCAAGGAACTAATACCAGTAACAGTTTGAATTAAAGCAGCAAACCCAGCAATAGCCCCAGGAGCACCAGCAATTACAGCATAT
GTTTGAGGAGTTAGGCCTATAGCAGCTATAGCCTCTGAGGTACTTGTTATGCCCTCTACAGTAGCAAGGGATGCAATTTGAACTTCTATAGCAGCAGCAGC
CTCCCCAGCAGCAATTTCAGCCACTGAAAATCCTGTGGCAGCAGCAGCCTCAGATACACTGGCAACTAGGTCCCCCAAAAGTGCTAGAGCAGCACCCATCA
ACCTGGAAATACAAAAAAAAAGGGATTACTTACCTAGGAGTCTTTTACAGAGTCTTTTACAGCAGGTAAAGCAGTGGTACTTTTGTTTTTCCCGTCTACACT
GTCTTCACCTCTACAAAATTCCAGCAAAAGCTCTAAAATAAAAATAAAAATCCTCTGAGCTCTTTTTTTGTTCCAGTCCAGGTTTTACTAACTTTCACAGAAGC
TTGTCGTGACAGCTGGCGCAGAACCATGGCCTTTGTCCAGTTTAACTGGGGACAAGGCCAAGATTCCTAGGCTCGCAAAACATGTCTGTCTGGCTGCTTTC
CACTCCTTTGGCCAGTTTCCACTTCCTCTTGTGTTTATTTGAGAATTTTAGGGGCGGGGTTTCACTATTAACTGCCACTGGCTGGCTGCCCAGTCATGCACTTT
CCTTCCTGAGGTCATGGTTTGGCTACATTCCATGGGTTAGCAGCTCCTCCCTGTGGCCTTTTTTTTTATAATATATAAGAGGCCGAGGCCGCCTCGGCCTCCA

CCCTTTCTCAGAAGTAGTAAGGGTGTGGAGGCTTTTTCTGAGGCCTAGCAAAAATATTTGGGGAAATCCCTATTCTTTTGCAATTTTTGCAAAAATGGATAA
AGTGCTTAACAGGGAAGAATCCATGGAGCTCATGGACCTTTTAGGCCTTGAAAGAGCTGCCTGGGGAAACCTTCCCTTAATGAGAAAAGCCTATTTAAGGA
AGTGTAAGGAATTTCACCCTGACAAAGGGGGCGACGAGGATAAAATGAAGAGAATGAATACTTTATATAAAAAAATGGAGCAGGATGTAAAGGTAGCTC
ATCAGCCTGATTTTGGAACCTGGAGTAGCTCAGAGGTTTGTGCTGATTTTCCTCTTTGCCCAGATACCCTGTACTGCAAGGATTGGCCTATTTGTTCCAAAAA
GCCTTCTGTGCACTGCCCTTGCATGCTATGTCAGCTTAGATTAAGGCATTTAAATAGAAAATTTTTAAGAAAAGAGCCCTTGGTTTGGATAGATTGCTACTG
CATTGACTGCTTCACACAGTGGTTTGGCTTAGACCTAACTGAAGAAACTCTGCAATGGTGGGTCCAAATAATTGGAGAAACTCCCTTCAGAGATCTAAAGC
TTTAAGGTAACTAACTTATATTTAGATAAATAAGAAAATATTTAAAGGCCCTAAGTAATTATTTTTTTTATAGGTGCCAACCTATGGAACAGAAGAGTGGGA
GTCCTGGTGGAGCTCCTTTAATGAAAAATGGGATGAAGATTTATTTTGCCATGAAGATATGTTTGCCAGTGATGAAGAAGCAACAGCAGATTCTCAACACT
CCACACCACCCAAGAAAAAGAGAAAGGTAGAAGACCCTAAAGACTTTCCTTCTGATCTACACCAGTTTCTTAGTCAAGCTGTATTTAGTAATAGAACCCTTG
CCTGCTTTGCTGTGTATACTACTAAAGAAAAAGCTCAAATTCTGTATAAAAAACTTATGGAAAAATATTCTGTAACTTTTATTAGTAGACACATGTGTGCTGG
GCATAATATTATATTCTTTTTAACTCCACATAGACATAGAGTTTCTGCAATTAATAATTTCTGTCAAAAGCTGTGTACCTTTAGTTTTTTAATTTGTAAGGGTG
TTAATAAGGAATACTTACTATATAGTGCCTTAACTAGAGATCCATACCATACAATAGAAGAAAGCATTCAAGGGGGCTTAAAGGAGCATGATTTTAACCCA
GAAGAGCCTGAAGAAACAAAGCAGGTGTCTTGGAAATTAATTACTGAGTATGCAGTAGAGACAAAGTGTGAGGATGTGTTCTTATTATTGGGTATGTATTT
AGAATTTCAATACAATGTAGAAGAGTGTAAAAAGTGTCAGAAAAAAGACCATGGACCTTATGCACCTTTAAGTATCATGAAAGAGCATGTTTGGCAAATGC
TACTATTTTTGCAGATAAGTAAAAATCAAAAAAGTATTTGTCAGCAAGCAGTAAGATACAGTTTTACGCTAAAAAAGGAGTAGATACCCTTCATATGACCAG
AGAAGAAATGCTAACAGAAAGATTTAATCATATATTAGATAAAATGGATTTAATATTTGGAGCTCATGGAAATGCTGTACTGGAACAATATAGTGGCAGGT
GTTGCTTGGCTGCACTGTTTGCTACCTAAAATGGATTCTGTTATATTCTGATTTTTTGCACTGTATTGTTTTTAATGTACCTAAAAGAAGATACTGGTTATTTA
CAAGGTCCCATTGATAGTGGAAAAACAACACTAGCTGCAGGGTTATTAGCATTTGTGTGGAGGTAAAGCCTTAAATGTAAACCTAGCCCATGGAAAGGCT
AACCTTTGAACTAGGTGTAGCTATAGCATCAGTACAGTGGTTTGTTTTTGAAGATGTAACTGAAGAAACTCTGCAATGGTGGGTCCAAATAATTGGAGAAA
CTCCCTTCAGAGATCTAAAGCTTTAAGGTAACTAACTTATATTTAGATAAATAAGAAAATATTTAAAGGCCCTAAGTAATTATTTTTAAATATATTTAAGAAA
ATCCTTACAAAACTCAGAGTTCTTACTTGAAAAAAGAATTTTACAAAGTGGAATGACCTTGTTGCTACTGCTAATTTGGTTTAGGCCTGTAGCTGATTTTGCA
ACAGATATACAATCTAGAATTGTTGAATGGAAGGAAAGGCTGGATTCTGAGATAAGTATGTATACTTTTTCAAGGATGAAATATAATATATGCATGGGGAA
ATGTATTCTTGATATCACAAGAGAAGAAGATTCAGAAACTGAAGACTCTGGACATGGATCAAGCACTGAATCCCAATCACAATGCTCTTCCCAAGTCTGAG
ATACTTCAGCCCCTGCTGAAGATTCCCAAAGGTCAGACCCCCATAGTCAAGAGTTGCATTTGTGTAAAGGCTTTCAATGTTTTAAAAGGCCTAAAACACCAC
CCCCAAAATAACACAAGCTTAAAAGTGGCTTATACAAAAGCAGCATTTATTAAATGTATATGTACAATAAAAGCACCTGTTTAAAGCATTTTGGTTTGCAAT
TGTCCTTGTTTATCAATATATCTTATCATATCTGGGTCCCCTGGAAGTCTTTCTGTGCCATCAAACACCCTAACCTCTTCTACCTGGGATTCCATACCATACATA
GGCTGCCCATCCACTCTCTGGGTTCTCCTGTTTATAAGGTCACTCAGCAAAAAGGAAATTGGGTAAGGATTTTTTACAGATCTTTTTCTCAGGCGAATCTTAA
AATATCTTGCAAGGCCTCTCCACTGTTGTGTTCCAGAGCTGTTAGTAAACAGGCCACAAATATCAGCAGCTGAAACATACAGGCTATCAGCTTTACAAAGAG
GCCCCACACCCTGTTCATCTAGCAACACTGTGGTAGCTGTGTTGGTCACATGAAGTACTGGGGGAACATTTTCCCCTCCTGTGAAAGTCCCAAAATACCTAG
TATTTTCATTTCTACTGGGATCAGGAACCCAGCACTCAACTGGATAAGCATTGTTTTTGTCCAAATAGGCCTTATGGTCAGTATTCATTACCTGGGACTGGGC
TGTTGGGTTTTTTGGGGTTATAGTACCATCTGGGTACTTGGTCCTGTAATTCATTAGCACTCCCTGCATTTCCAAGGGGTCTCCACCAACAGCAAAGAAGTG
GAAATTACTGCCTTGAATAGGTTTACCTCCACCATGCTCATGCACTTTTTGTGACCCTGCATGAAGGTTAAGCATGCTAGTTATTCCAATGACCTCTGTTTGT
ACAGTTACAGCCTCCCACATCAGTAGATTTCCACAGGTTAGGTCCTCATTTAAATTGGGGAGGGGAATTCTTGCTGTGCTGTAACAGGGAAGCATTTTTCTT
TCTGGGCTATCACTGCTAAAGTCATTTTCAGCACTTAGCTTTAGACTAAAGCCCCTAAGGTTTTCATCTGGATCCCCCATTTCTGGGTTTAGGAAGCATTCTA
CCTCTGTTATAGCATCTACCCCAGTTTTAACTTCTAGAACTTCTACTCCTCCTTTTATTAGTAGTTTTGGCACTTGCACGGGTTCCTTTGGCTTTTTGGGAGCTG
CCCCTGGACACTCTCCTTTTCTTTTGGTTGGGGCCATCTTCATATGCTTCAAGAGCAGGTGTTACAGTCCCGTACAGGCCTAGAAGTAAAGGCAACATCCAT
TGAGGAGCAGTTCTTTGATTAGCACCTCCTGGGGCAATAGTTTTTTCTATAAACTCTCCTGAATGTACAGTTTGTTGATTTCTTAAGTCCATTCTTTGTGTAAC
TTCTTCTATACTGTCAGCATCATCTATACTATAAGTATGGCCAAAATGTACACGGGTACCTTCCCTTTCAGCCACTTGTCTAACCATTGAGGGCCTAATAGGG
GAAAGATCAGAATAATATTCTTGAATATAATTATAAAAGTTTATAGGGGCATTTACAATTGTCCAGGTAGTTTCCTCCAAAAATCTAGCCAAGGAGTCTCTA
AAAAATCTTTCTGTTCTTCTTTGCAATTCTTGTGAGGTTATAGCAGGTATATCATCCCTAAAGTATTCATTCTCTTCATTTTATCCTCGTCGCCCCCTTTGTCAG
GGTGAAATTCCTTACACTTCCTTAAATAGGCTTTTCTCATTAAGGGAAGGTTTCCCCAGGCAGCTCTTTCAAGGCCTAAAAGGTCCATGAGCTCCATGGATT
CTTCCCTGTTAAGCACTTTATCCATTTTTGCAAAAATTGCAAAAGAATAGGGATTTCCCCAAATATTTTTGCTAGGCCTCAGAAAAAGCCTCCACACCCTTAC
TACTTCAGAGAAAGGGTGGAGGCCGAGAGACAATACGAAACTGAACCAAGTAGGTTAGAAATACGTATTGCTGTTTTACACATTCTGTGCCCCCTTACTCT
ATCTTTTTTTCTTTTTGGAGTTAATTATTATTTATTTTGCCTACATTTTAATTATATTATGAAAATGTTATCTTAGTAGTACTC

Sequence for circular contig. #1 as shown in figure 4)

GCCTCGGCCTCTTATATATTATAAAAAAAAAGGCCACAGGGAGGAGCTGCTAACCCATGGAATGTAGCCAAACCATGACCTCAGGAAGGAAAGTGCATGA
CTGGGCAGCCAGCCAGTGGCAGTTAATAGTGAAACCCCGCCCCTAAAATTCTCAAATAAACACAAGAGGAAGTGGAAACTGGCCAAAGGAGTGGAAAGC
AGCCAGACAGACATGTTTTGCGAGCCTAGGAATCTTGGCCTTGTCCCCAGTTAAACTGGACAAAGGCCATGGGGGTTTCACTATTAACTGCCACTGGCTGG
CTGCCCAGTCATGCACTTTCCTTCCTGAGGTCATGGTTTGGCTACATTCCATGGGTTAGCAGCTCCTCCCTGTGGCCTTTTTTTTTATAATATATAAGAGGCC
GAGGCCGCCTCGGCCTCCACCCTTTCTCTGAAGTAGTAAGGGTGTGGAGGCTTTTTCTGAGGCCTAGCAAAAATATTTGGGGAAATCCCTATTCTTTTGCAA
TTTTTGCAAAAATGGATAAAGTGCTTAACAGGGAAGAATCCATGGAGCTCATGGACCTTTTAGGCCTTGAAAGAGCTGCCTGGGGAAACCTTCCCTTAATG
AGAAAAGCCTATTTAAGGAAGTGTAAGGAATTTCACCCTGACAAAGGGGGCGACGAGGATAAAATGAAGAGAATGAATACTTTAGGGATGATATACCTG
CTATAACCTCACAAGAATTGCAAAGAAGAACAGAAAGATTTTTTAGAGACTCCTTGGCTAGATTTTTGGAGGAAACTACCTGGACAATTGTAAATGCCCCTA
TAAACTTTTATAATTATATTCAAGAATATTATTCTGATCTTTCCCCTATTAGGCCCTCAATGGTTAGACAAGTGGCTGAAAGGGAAGGTACCCGTGTACATTT
TGGCCATACTTATAGTATAGATGATGCTGACAGTATAGAAGAAGTTACACAAAGAATGGACTTAAGAAATCAACAAACTGTACATTCAGGAGAGTTTATAG
AAAAAACTATTGCCCCAGGAGGTGCTAATCAAAGAACTGCTCCTCAATGGATGTTGCCTTTACTTCTAGGCCTGTACGGGACTGTAACACCTGCTCTTGAAG
CATATGAAGATGGCCCCAACCAAAAGAAAAGGAGAGTGTCCAGGGGCAGCTCCCAAAAAGCCAAAGGAACCCGTGCAAGTGCCAAAACTACTAATAAAA
GGAGGAGTAGAAGTTCTAGAAGTTAAAACTGGGGTAGATGCTATAACAGAGGTAGAATGCTTCCTAAACCCAGAAATGGGGGATCCAGATGAAAACCTT
AGGGGCTTTAGTCTAAAGCTAAGTGCTGAAAATGACTTTAGCAGTGATAGCCCAGAAAGAAAAATGCTTCCCTGTTACAGCACAGCAAGAATTCCCCTCCC
CAATTTAAATGAGGACCTAACCTGTGGAAATCTACTGATGTGGGAGGCTGTAACTGTACAAACAGAGGTCATTGGAATAACTAGCATGCTTAACCTTCATG
CAGGGTCACAAAAAGTGCATGAGCATGGTGGAGGTAAACCTATTCAAGGCAGTAATTTCCACTTCTTTGCTGTTGGTGGAGACCCCTTGGAAATGCAGGG
AGTGCTAATGAATTACAGGACCAAGTACCCAGATGGTACTATAACCCCAAAAAACCCAACAGCCCAGTCCCAGGTAATGAATACTGACCATAAGGCCTATT
TGGACAAAAACAATGCTTATCCAGTTGAGTGCTGGGTTCCTGATCCCAGTAGAAATGAAAATACTAGGTATTTTGGGACTTTCACAGGAGGGGAAAATGTT

CCCCCAGTACTTCATGTGACCAACACAGCTACCACAGTGTTGCTAGATGAACAGGGTGTGGGGCCTCTTTGTAAAGCTGATAGCCTGTATGTTTCAGCTGCT
GATATTTGTGGCCTGTTTACTAACAGCTCTGGAACACAACAGTGGAGAGGCCTTGCAAGATATTTTAAGATTCGCCTGAGAAAAAGATCTGTAAAAAATCC
TTACCCAATTTCCTTTTTGCTGAGTGACCTTATAAACAGGAGAACCCAGAGAGTGGATGGGCAGCCTATGTATGGTATGGAATCCCAGGTAGAAGAGGTTA
GGGTGTTTGATGGCACAGAAAGACTTCCAGGGGACCCAGATATGATAAGATATATTGATAAACAAGGACAATTGCAAACCAAAATGCTTTAAACAGGTGC
TTTTATTGTACATATACATTTAATAAATGCTGCTTTTGTATAAGCCACTTTTAAGCTTGTGTTATTTTGGGGGTGGTGTTTTAGGCCTTTTAAAACATTGAAAG
CCTTTACACAAATGCAACTCTTGACTATGGGGGTCTGACCTTTGGGAATCTTCAGCAGGGGCTGAAGTATCTGAGACTTGGGAAGAGCATTGTGATTGGGA
TTCAGTGCTTGATCCATGTCCAGAGTCTTCAGTTTCTGAATCTTCTTCTCTTGTGATATCAAGAATACATTTCCCCATGCATATATTATATTTCATCCTTGAAAA
AGTATACATACTTATCTCAGAATCCAGCCTTTCCTTCCATTCAACAATTCTAGATTGTATATCTGTTGCAAAATCAGCTACAGGCCTAAACCAAATTAGCAGT
AGCAACAAGGTCATTCCACTTTGTAAAATTCTTTTTTCAAGTAAGAACTCTGAGTTTTGTAAGGATTTTCTTAAATATATTTTGGGTCTAAAATCTATCTGTCT
TACAAATCTAGCCTGCAGGGTTTTAGGAACAGGATACTCATTCATTGTAACCAGGCCTGGTGGAAATATTTGGGTTCTTTTGTTTAAATGTTTCTTTTCTAAA
TTAACCTTAACACTTCCATCTAAATAATCTCTCAAACTGTCTAAATTGTTTATTCCATGTCCTGAAGGCAGATCCTTTGATTCAGCCCCAGTTCCTTTTACATCT
TCAAAAACAACCATGTACTGATCTATAGCTACACCTAGTTCAAAGGTTAGCCTTTCCATGGGTAGGTTTACATTTAAGGCTTTACCTCCACACAAATCTAATA
ACCCTGCAGCTAGTGTTGTTTTTCCACTATCAATGGGACCTTTAAATAACCAGTATCTTCTTTTAGGTACATTAAAAACAATACAGTGCAAAAAATCAAATAT
AACAGAATCCATTTTAGGTAGCAAACAGTGCAGCCAAGCAACACCTGCCATATATTGTTCCAGTACAGCATTTCCATGAGCTCCAAATATTAAATCCATTTTA
TCTAATATATGATTAAATCTTTCTGTTAGCATTTCTTCTCTGGTCATATGAAGGGTATCTACTCTTTTTTTAGCTAAAACTGTATCTACTGCTTGCTGACAAATA
CTTTTTTGATTTTTACTTTCTGCAAAAATAGTAGCATTTGCAAAATGCTTTTCATGATACTTAAAGTGATAAGGTTGGTCTTTTTTCTGACACTTTTTACACTCT
TCTACATTGTATTGAAATTCTAAATACATACCCAATAATAAGAACACATCCTCACACTTTGTCTCTACTGCATACTCAGTAATTAATTTCCAAGACACCTGCTT
TGTTTCTTCAGGCTCTTCTGGGTTAAAATCATGCTCCTTTAAGCCCCCTTGAATGCTTTCTTCTATTGTATGGTATGGATCTCTAGTTAAGGCACTATATAGTA
AGTATTCCTTATTAACACCCTTACAAATTAAAAAACTAAAGGTACACAGCTTTTGACAGAAATTATTAATTGCAGAAACTCTATGTCTATGTGGAGTTAAAAA
GAATATAATATTATGCCCAGCACACATGTGTCTACTAATAAAAGTTACAGAATATTTTTCCATAAGTTTTTTATACAGAATTTGAGCTTTTTCTTTAGTAGTAT
ACACAGCAAAGCAGGCAAGGGTTCTATTACTAAATACAGCTTGACTAAGAAACTGGTGTAGATCAGAAGGAAAGTCTTTAGGGTCTTCTACCTTTCTCTTTT
TCTTGGGTGGTGTGGAGTGTTGAGAATCTGCTGTTGCTTCTTCATCACTGGCAAACATATCTTCATGGCAAAATAAATCTTCATCCCATTTTTCATTAAAGGA
GCTCCACCAGGACTCCCACTCTTCTGTTCCATAGGTTGGCACCTATAAAAAAAATAATTACTTAGGGCCTTTAAATATTTTCTTATTTATCTAAATATAAGTTA
GTTACCTTAAAGCTTTAGATCTCTGAAGGGAGTTTCTCCAATTATTTGGACCCACCATTGCAGAGTTTCTTCAGTTAGGTCTAAGCCAAACCACTGTGTGAAG
CAGTCAATGCAGTAGCAATCTATCCAAACCAAGGGCTCTTTTCTTAAAAATTTTCTATTTAAATGCCTTAATCTAAGCTGACATAGCATGCAAGGGCAGTGC
ACAGAAGGCTTTTTGGAACAAATAGGCCAATCCTTGCAGTACAGGGTATCTGGGCAAAGAGGAAAATCAGCACAAACCTCTGAGCTACTCCAGGTTCCAA
AATCAGGCTGATGAGCTACCTTTACATCCTGCTCCATTTTTTTATATAAAGTATTCATTCTCTTCATTTTATCCTCGTCGCCCCCTTTGTCAGGGTGAAATTCCT
TACACTTCCTTAAATAGGCTTTTCTCATTAAGGGAAGGTTTCCCCAGGCAGCTCTTTCAAGGCCTAAAAGGTCCATGAGCTCCATGGATTCTTCCCTGTTAAG
CACTTTATCCATTTTTGCAAAAATTGCAAAAGAATAGGGATTTCCCCAAATATTTTTGCTAGGCCTCAGAAAAAGCCTCCACACCCTTACTACTTCTGAGAAA
GGGTGGAGGCCGAGGCG

Sequence for circular contig. #2 as shown in figure 5

CCTCGGCCTCTTATATATTATAAAAAAAAAAGGCCACAGGGAGGAGCTGCTAACCCATGGAATGTAGCCAAACCATGACCTCAGGAAGGAAAGTGCATGAC
TGGGCAGCCAGCCAGTGGCAGTTAATAGTGAAACCCCGCCCCTAAAATTCTCAAATAAACACAAGAGGAAGTGGAAACTGGCCAAAGGAGTGGAAAGCA
GCCAGACAGACATGTTTTGCGAGCCTAGGAATCTTGGCCTTGTCCCCAGTTAAACTGGACAAAGGCCATGGTTCTGCGCCAGCTGTCACGACAAGCTTCTG
TGAAAGTTAGTAAAACCTGGACTGGAACAAAAAAAAGAGCTCAGAGGATTTTTATTTTTATTTTAGAGCTTTTGCTGGAATTTTGTAGAGGTGAAGACAGT
GTAGACGGGAAAAACAAAAGTACCACTGCTTTACCTGCTGTAAAAGACTCTGTAAAAGACTCCTAGGTAAGTAATCCCTTTTTTTTTGTATTTCCAGGTTGA
TGGGTGCTGCTCTAGCACTTTTGGGGGACCTAGTTGCCAGTGTATCTGAGGCTGCTGCTGCCACAGGATTTTCAGTGGCTGAAATTGCTGCTGGGGAGGCT
GCTGCTGCTATAGAAGTTCAAATTGCATCCCTTGCTACTGTAGAGGGCATAACAAGTACCTCAGAGGCTATAGCTGCTATAGGCCTAACTCCTCAAACATAT
GCTGTAATTGCTGGTGCTCCTGGGGCTATTGCTGGGTTTGCTGCTTTAATTCAAACTGTTACTGGTATTAGTTCCTTGGCTCAAGTAGGGTATAGGTTTTTTA
GTGATTGGGATCACAAAGTTTCCACTGTAGGCCTCTATCAGCAATCAGGCATGGCTTTGGAATTGTTTAACCCAGATGAGTACTATGATATTTTGTTTCCTG
GTGTAAATACTTTTGTAAATAATATTCAATACCTTGATCCTAGGCATTGGGGTCCTTCCTTGTTTGCTACTATTTCCCAGGCTTTGTGGCATGTTATTAGGGAT
GATATACCTGCTATAACCTCACAAGAATTGCAAAGAAGAACAGAAAGATTTTTTAGAGACTCCTTGGCTAGATTTTTGGAGGAAACTACCTGGACAATTGT
AAATGCCCCTATAAACTTTTATAATTATATTCAAGAATATTATTCTGATCTTTCCCCTATTAGGCCCTCAATGGTTAGACAAGTGGCTGAAAGGGAAGGTACC
CGTGTACATTTTGGCCATACTTATAGTATAGATGATGCTGACAGTATAGAAGAAGTTACACAAAGAATGGACTTAAGAAATCAACAAACTGTACATTCAGG
AGAGTTTATAGAAAAAACTATTGCCCCAGGAGGTGCTAATCAAAGAACTGCTCCTCAATGGATGTTGCCTTTACTTCTAGGCCTGTACGGGACTGTAACACC
TGCTCTTGAAGCATATGAAGATGGCCCCAACCAAAAGAAAAGGAGAGTGTCCAGGGGCAGCTCCCAAAAAGCCAAAGGAACCCGTGCAAGTGCCAAAAC
TACTAATAAAAGGAGGAGTAGAAGTTCTAGAAGTTAAAACTGGGGTAGATGCTATAACAGAGGTAGAATGCTTCCTAAACCCAGAAATGGGGGATCCAG
ATGAAAACCTTAGGGGCTTTAGTCTAAAGCTAAGTGCTGAAAATGACTTTAGCAGTGATAGCCCAGAAAGAAAAATGCTTCCCTGTTACAGCACAGCAAGA
ATTCCCCTCCCCAATTTAAATGAGGACCTAACCTGTGGAAATCTACTGATGTGGGAGGCTGTAACTGTACAAACAGAGGTCATTGGAATAACTAGCATGCT
TAACCTTCATGCAGGGTCACAAAAAGTGCATGAGCATGGTGGAGGTAAACCTATTCAAGGCAGTAATTTCCACTTCTTTGCTGTTGGTGGAGACCCCTTGG
AAATGCAGGGAGTGCTAATGAATTACAGGACCAAGTACCCAGATGGTACTATAACCCCAAAAAACCCAACAGCCCAGTCCCAGGTAATGAATACTGACCAT
AAGGCCTATTTGGACAAAAACAATGCTTATCCAGTTGAGTGCTGGGTTCCTGATCCCAGTAGAAATGAAAATACTAGGTATTTTGGGACTTTCACAGGAGG
GGAAAATGTTCCCCCAGTACTTCATGTGACCAACACAGCTACCACAGTGTTGCTAGATGAACAGGGTGTGGGGCCTCTTTGTAAAGCTGATAGCCTGTATG
TTTCAGCTGCTGATATTTGTGGCCTGTTTACTAACAGCTCTGGAACACAACAGTGGAGAGGCCTTGCAAGATATTTTAAGATTCGCCTGAGAAAAAGATCTG
TAAAAAATCCTTACCCAATTTCCTTTTTGCTGAGTGACCTTATAAACAGGAGAACCCAGAGAGTGGATGGGCAGCCTATGTATGGTATGGAATCCCAGGTA
GAAGAGGTTAGGGTGTTTGATGGCACAGAAAGACTTCCAGGGGACCCAGATATGATAAGATATATTGATAAACAAGGACAATTGCAAACCAAAATGCTTT
AAACAGGTGCTTTTATTGTACATATACATTTAATAAATGCTGCTTTTGTATAAGCCACTTTTAAGCTTGTGTTATTTTGGGGGTGGTGTTTTAGGCCTTTTAAA
ACATTGAAAGCCTTTACACAAATGCAACTCTTGACTATGGGGGTCTGACCTTTGGGAATCTTCAGCAGGGGCTGAAGTATCTCAGACTTGGGAAGAGCATT
GTGATTGGGATTCAGTGCTTGATCCATGTCCAGAGTCTTCAGTTTCTGAATCTTCTTCTCTTGTGATATCAAGAATACATTTCCCCATGCATATATTATATTTC
ATCCTTGAAAAAGTATACATACTTATCTCAGAATCCAGCCTTTCCTTCCATTCAACAATTCTAGATTGTATATCTGTTGCAAAATCAGCTACAGGCCTAAACCA
AATTAGCAGTAGCAACAAGGTCATTCCACTTTGTAAAATTCTTTTTTCAAGTAAGAACTCTGAGTTTTGTAAGGATTTTCTTAAATATATTTAAAAATAATTAC
TTAGGGCCTTTAAATATTTTCTTATTTATCTAAATATAAGTTAGTTACCTTAAAGCTTTAGATCTCTGAAGGGAGTTTCTCCAATTATTTGGACCCACCATTGC
AGAGTTTCTTCAGTTAGGTCTAAGCCAAACCACTGTGTGAAGCAGTCAATGCAGTAGCAATCTATCCAAACCAAGGGCTCTTTTCTTAAAAATTTTCTATTTA
AATGCCTTAATCTAAGCTGACATAGCATGCAAGGGCAGTGCACAGAAGGCTTTTTGGAACAAATAGGCCAATCCTTGCAGTACAGGGTATCTGGGCAAAG
AGGAAAATCAGCACAAACCTCTGAGCTACTCCAGGTTCCAAAATCAGGCTGATGAGCTACCTTTACATCCTGCTTCATTTTTTTATATAAAGTATTCATTCTCT

TCATTTTATCCTCGTCGCCCCCTTTGTCAGGGTGAAATTCCTTACACTTCCTTAAATAGGCTTTTCTCATTAAGGGAAGGTTTCCCCAGGCAGCTCTTTCAAGG
CCTAAAAGGTCCATGAGCTCCATGGATTCTTCCCTGTTAAGCACTTTATCCATTTTTGCAAAAATTGCAAAAGAATAGGGATTTCCCCAAATATTTTTGCTAG
GCCTCAGAAAAAGCCTCCACACCCTTACTACTTCTGAGAAAGGGTGGAGGCCGAGGCGGCCTCGGCCTCTTATATATTATAAAAAAAAAGGCCACAGGGA
GGAGCTGCTAACCCATGGAATGTAGCCAAACCATGACCTCAGGAAGGAAAGTGCATGACTGGGCAGCCAGCCAGTGGCAGTTAATAGTGAAACCCCGCC
CCTAAAATTCTCAAATAAACACAAGAGGAAGTGGAAACTGGCCAAAGGAGTGGAAAGCAGCCAGACAGAGATCAGAAGGAAAGTCTTTAGGGTCTTCTA
CCTTTCTCTTTTTCTTGGGTGGTGTGGAGTGTTGAGAATCTGCTGTTGCTTCTTCATCACTGGCAAACATATCTTCATGGCAAAATAAATCTTCATCCCATTTT
TCATTAAAGGAGCTCCACCAGGACTCCCACTCTTCTGTTCCATAGGTTGGCACCTATAAAAAAAATAATTACTTAGGGCCTTTAAATATTTTCTTATTTATCTA
AATATAAGTTAGTTACCTTAAAGCTTTAGATCTCTGAAGGGAGTTTCTCCAATTATTTGGACCCACCATTGCAGAGTTTCTTCAGTTAGGTCTAAGCCAAACC
ACTGTGTGAAGCAGTCAATGCAGTAGCAATCTATCCAAACCAAGGGCTCTTTTCTTAAAAATTTTCTATTTAAATGCCTTAATCTAAGCTGACATAGCATGCA
AGGGCAGTGCACAGAAGGCTTTTTGGAACAAATAGGCCAATCCTTGCAGTACAGGGTATCTGGGCAAAGAGGAAAATCAGCACAAACCTCTGAGCTACTC
CAGGTTCCAAAATCAGGCTGATGAGCTACCTTTACATCCTGCTCCATTTTTTTATATAAAGTATTCATTCTCTTCATTTTATCCTCGTCGCCCCCTTTGTCAGGG
TGAAATTCCTTACACTTCCTTAAATAGGCTTTTCTCATTAAGGGAAGGTTTCCCCAGGCAGCTCTTTCAAGGCCTAAAAGGTCCATGAGCTCCATGGATTCTT
CCCTGTTAAGCACTTTATCCATTTTTGCAAAAATTGCAAAAGAATAGGGATTTCCCCAAATATTTTTGCTAGGCCTCAGAAAAAGCCTCCACACCCTTACTAC
TTCTGAGAAAGGGTGGAGGCCGAGGCGG

Sequence for circular contig. #3 as shown in figure 6

GCCTCGGCCTCTTATATATTATAAAAAAAAAGGCCACAGGGAGGAGCTGCTAACCCATGGAATGTAGCCAAACCATGACCTCAGGAAGGAAAGTGCATGA
CTGGGCAGCCAGCCAGTGGCAGTTAATAGTGAAACCCCGCCCCTAAAATTCTCAAATAAACACAAGAGGAAGTGGAAACTGGCCAAAGGAGTGGAAAGC
AGCCAGACAGACATGTTTTGCGAGCCTAGGAATCTTGGCCTTGTCCCCAGTTAAACTGGACAAAGGCCTGCCACTGGCTGGCTGCCCAGTCATGCACTTTC
CTTCCTGAGGTCATGGTTTGGCTACATTCCATGGGTTAGCAGCTCCTCCCTGTGGCCTTTTTTTTTATAATATATAAGAGGCCGAGGCCGCCTCGGCCTCCAC
CCTTTCTCAGAAGTAGTAAGGGTGTGGAGGCTTTTTCTGAGGCCTAGCAAAAATATTTGGGGAAATCCCTATTCTTTTGCAATTTTTGCAAAAATGGATAAA
GTGCTTAACAGGGAAGAATCCATGGAGCTCATGGACCTTTTAGGCCTTGAAAGAGCTGCCTGGGGAAACCTTCCCTTAATGAGAAAAGCCTATTTAAGGAA
GTGTAAGGAATTTCACCCTGACAAAGGGGGCGACGAGGATAAAATGAAGAGAATGAATACTTTATATAAAAAAATGGAGCAGGATGTAAAGGTAGCTCA
TCAGCCTGATTTTGGAACCTGGAGTAGCTCAGAGGTTTGTGCTGATTTTCCTCTTTGCCCAGATACCCTGTACTGCAAGGATTGGCCTATTTGTTCCAAAAA
GCCTTCTGTGCACTGCCCTTGCATGCTATGTCAGCTTAGATTAAGGCATTTAAATAGAAAATTTTTAAGAAAAGAGCCCTTGGTTTGGATAGATTGCTACTG
CATTGACTGCTTCACACAGTGGTTTGGCTTAGACCTAACTGAAGAAACTCTGCAATGGTGGGTCCAAATAATTGGAGAAACTCCCTTCAGAGATCTAAAGC
TTTAAGGTAACTAACTTATATTTAGATAAATAAGAAAATATTTAAAGGCCCTAAGTAATTATTTTTTTTATAGGTGCCAACCTATGGAACAGAAGAGTGGGA
GTCCTGGTGGAGCTCCTTTAATGAAAAATGGGATGAAGATTTATTTTGCCATGAAGATATGTTTGCCAGTGATGAAGAAGCAACAGCAGATTCTCAACACT
CCACACCACCCAAGAAAAAGAGAAAGGTAGAAGACCCTAAAGACTTTCCTTCTGATCTACACCAGTTTCTTAGTCAAGCTGTATTTAGTAATAGAACCCTTG
CCTGCTTTGCTGTGTATACTACTAAAGAAAAAGCTCAAATTCTGTATAAAAAACTTATGGAAAAATATTCTGTAACTTTTATTAGTAGACACATGTGTGCTGG
GCATAATATTATATTCTTTTTAACTCCACATAGACATAGAGTTTCTGCAATTAATAATTTCTGTCAAAAGCTGTGTACCTTTAGTTTTTTAATTTGTAAGGGTG
TTAATAAGGAATACTTACTATATAGTGCCTTAACTAGAGATCCATACCATACAATAGAAGAAAGCATTCAAGGGGGCTTAAAGGAGCATGATTTTAACCCA
GAAGAGCCTGAAGAAACAAAGCAGGTGTCTTGGAAATTAATTACTGAGTATGCAGTAGAGACAAAGTGTGAGGATGTGTTCTTATTATTGGGTATGTATTT
AGAATTTCAATACAATGTAGAAGAGTGTAAAAAGTGTCAGAAAAAAGACCAACCTTATCACTTTAAGTATCATGAAAAGCATTTTGCAAATGCTACTATTTT
TGCAGAAAGTAAAAATCAAAAAAGTATTTGTCAGCAAGCAGTAGATACAGTTTTAGCTAAAAAAAGAGTAGATACCCTTCATATGACCAGAGAAGAAATG
CTAACAGAAAGATTTAATCATATATTAGATAAAATGGATTTAATATTTGGAGCTCATGGAAATGCTGTACTGGAACAATATATGGCAGGTGTTGCTTGGCT
GCACTGTTTGCTACCTAAAATGGATTCTGTTATATTTGATTTTTTGCACTGTATTGTTTTTAATGTACCTAAAAGAAGATACTGGTTATTTAAAGGTCCCATTG
ATAGTGGAAAAACAACACTAGCTGCAGGGTTATTAGATTTGTGTGGAGGTAAAGCCTTAAATGTAAACCTACCCATGGAAAGGCTAACCTTTGAACTAGGT
GTAGCTATAGATCAGTACATGGTTGTTTTTGAAGATGTAAAAGGAACTGGGGCTGAATCAAAGGATCTGCCTTCAGGACATGGAATAAACAATTTAGACA
GTTTGAGAGATTATTTAGATGGAAGTGTTAAGGTTAATTTAGAAAAGAAACATTTAAACAAAAGAACCCAAATATTTCCACCAGGCCTGGTTACAATGAAT
GAGTATCCTGTTCCTAAAACCCTGCAGGCTAGATTTGTAAGACAGATAGATTTTAGACCCAAAATATATTTAAGAAAATCCTTACAAAACTCAGAGTTCTTA
CTTGAAAAAAGAATTTTACAAAGTGGAATGACCTTGTTGCTACTGCTAATTTGGTTTAGGCCTGTAGCTGATTTTGCAACAGATATACAATCTAGAATTGTT
GAATGGAAGGAAAGGCTGGATTCTGAGATAAGTATGTATACTTTTTCAAGGATGAAATATAATATATGCATGGGGAAATGTATTCTTGATATCACAAGAGA
AGAAGATTCAGAAACTGAAGACTCTGGACATGGATCAAGCACTGAATCCCAATCACAATGCTCTTCCCAAGTCTCAGATACTTCAGCCCCTGCTGAAGATTC
CCAAAGGTCAGACCCCCATAGTCAAGAGTTGCATTTGTGTAAAGGCTTTCAATGTTTTAAAAGGCCTAAAACACCACCCCCAAAATAACACAAGCTTAAAA
GTGGCTTATACAAAAGCAGCATTTATTAAATGTATATGTACAATAAAAGCACCTGTTTAAAGCATTTTGGTTTGCAATTGTCCTTGTTTATCAATATATCTTAT
CATATCTGGGTCCCCTGGAAGTCTTTCTGTGCCATCAAACACCCTAACCTCTTCTACCTGGGATTCCATACCATACATAGGCTGCCCATCCACTCTCTGGGTTC
TCCTGTTTATAAGGTCACTCAGCAAAAAGGAAATTGGGTAAGGATTTTTTACAGATCTTTTTCTCAGGCGAATCTTAAAATATCTTGCAAGGCCTCTCCACTG
TTGTGTTCCAGAGCTGTTAGTAAACAGGCCACAAATATCAGCAGCTGAAACATACAGGCTATCAGCTTTACAAAGAGGCCCCACACCCTGTTCATCTAGCAA
CACTGTGGTAGCTGTGTTGGTCACATGAAGTACTGGGGGAACATTTTCCCCTCCTGTGAAAGTCCCAAAATACCTAGTATTTTCATTTCTACTGGGATCAGG
AACCCAGCACTCAACTGGATAAGCATTGTTTTTGTCCAAATAGGCCTTATGGTCAGTATTCATTACCTGGGACTGGGCTGTTGGGTTTTTTGGGGTTATAGT
ACCATCTGGGTACTTGGTCCTGTAATTCATTAGCACTCCCTGCATTTCCAAGGGGTCTCCACCAACAGCAAAGAAGTGGAAATTACTGCCTTGAATAGGTTT
ACCTCCACCATGCTCATGCACTTTTTGTGACCCTGCATGAAGGTTAAGCATGCTAGTTATTCCAATGACCTCTGTTTGTACAGTTACAGCCTCCCACATCAGT
AGATTTCCACAGGTTAGGTCCTCATTTAAATTGGGGAGGGGAATTCTTGCTGTGCTGTAACAGGGAAGCATTTTTCTTTCTGGGCTATCACTGCTAAAGTCA
TTTTCAGCACTTAGCTTTAGACTAAAGCCCCTAAGGTTTTCATCTGGATCCCCCATTTCTGGGTTTAGGAAGCATTCTACCTCTGTTATAGCATCTACCCCAGT
TTTAACTTCTAGAACTTCTACTCCTCCTTTTATTAGTAGTTTTGGCACTTGCACGGGTTCCTTTGGCTTTTTGGGAGCTGCCCCTGGACACTCTCCTTTTCTTTT
GGTTGGGGCCATCTTCATATGCTTCAAGAGCAGGTGTTACAGTCCCGTACAGGCCTAGAAGTAAAGGCAACATCCATTGAGGAGCAGTTCTTTGATTAGCA
CCTCCTGGGGCAATAGTTTTTTCTATAAACTCTCCTGAATGTACAGTTTGTTGATTTCTTAAGTCCATTCTTTGTGTAACTTCTTTTATACTGTCAGCATCATCT
ATACTATAAGTATGGCCAAAATGTACACGGGTACCTTCCCTTTCAGCCACTTGTCTAACCATTGAGGGCCTAATAGGGGAAAGATCAGAATAATATTCTTGA
ATATAATTATAAAAGTTTATAGGGGCATTTACAATTGTCCAGGTAGTTTATCCATTTTTGCAAAAATTGCAAAAGAATAGGGATTTCCCCAAATATTTTTGCT
AGGCCTCAGAAAAAGCCTCCACACCCTTACTACTTCTGAGAA
